# Pandemic preparedness and COVID-19: an exploratory analysis of infection and fatality rates, and contextual factors associated with preparedness in 177 countries, from Jan 1, 2020, to Sept 30, 2021

**DOI:** 10.1016/S0140-6736(22)00172-6

**Published:** 2022-04-16

**Authors:** COVID-19 National Preparedness Collaborators, Thomas J Bollyky, Erin N Hulland, Ryan M Barber, James K Collins, Samantha Kiernan, Mark Moses, David M Pigott, Robert C Reiner Jr, Reed J D Sorensen, Cristiana Abbafati, Christopher Adolph, Adrien Allorant, Joanne O Amlag, Aleksandr Y Aravkin, Bree Bang-Jensen, Austin Carter, Rachel Castellano, Emma Castro, Suman Chakrabarti, Emily Combs, Xiaochen Dai, William James Dangel, Carolyn Dapper, Amanda Deen, Bruce B Duncan, Lucas Earl, Megan Erickson, Samuel B Ewald, Tatiana Fedosseeva, Alize J Ferrari, Abraham D Flaxman, Nancy Fullman, Emmanuela Gakidou, Bayan Galal, John Gallagher, John R Giles, Gaorui Guo, Jiawei He, Monika Helak, Bethany M Huntley, Bulat Idrisov, Casey Johanns, Kate E LeGrand, Ian D Letourneau, Akiaja Lindstrom, Emily Linebarger, Paulo A Lotufo, Rafael Lozano, Beatrice Magistro, Deborah Carvalho Malta, Johan Månsson, Ana M Mantilla Herrera, Fatima Marinho, Alemnesh H Mirkuzie, Ali H Mokdad, Lorenzo Monasta, Paulami Naik, Shuhei Nomura, James Kevin O'Halloran, Christopher M Odell, Latera Tesfaye Olana, Samuel M Ostroff, Maja Pasovic, Valeria Maria de Azeredo Passos, Louise Penberthy, Grace Reinke, Damian Francesco Santomauro, Maria Inês Schmidt, Aleksei Sholokhov, Emma Spurlock, Christopher E Troeger, Elena Varavikova, Anh T Vo, Theo Vos, Rebecca Walcott, Ally Walker, Simon D Wigley, Charles Shey Wiysonge, Nahom Alemseged Worku, Yifan Wu, Sarah Wulf Hanson, Peng Zheng, Simon I Hay, Christopher J L Murray, Joseph L Dieleman

## Abstract

**Background:**

National rates of COVID-19 infection and fatality have varied dramatically since the onset of the pandemic. Understanding the conditions associated with this cross-country variation is essential to guiding investment in more effective preparedness and response for future pandemics.

**Methods:**

Daily SARS-CoV-2 infections and COVID-19 deaths for 177 countries and territories and 181 subnational locations were extracted from the Institute for Health Metrics and Evaluation's modelling database. Cumulative infection rate and infection-fatality ratio (IFR) were estimated and standardised for environmental, demographic, biological, and economic factors. For infections, we included factors associated with environmental seasonality (measured as the relative risk of pneumonia), population density, gross domestic product (GDP) per capita, proportion of the population living below 100 m, and a proxy for previous exposure to other betacoronaviruses. For IFR, factors were age distribution of the population, mean body-mass index (BMI), exposure to air pollution, smoking rates, the proxy for previous exposure to other betacoronaviruses, population density, age-standardised prevalence of chronic obstructive pulmonary disease and cancer, and GDP per capita. These were standardised using indirect age standardisation and multivariate linear models. Standardised national cumulative infection rates and IFRs were tested for associations with 12 pandemic preparedness indices, seven health-care capacity indicators, and ten other demographic, social, and political conditions using linear regression. To investigate pathways by which important factors might affect infections with SARS-CoV-2, we also assessed the relationship between interpersonal and governmental trust and corruption and changes in mobility patterns and COVID-19 vaccination rates.

**Findings:**

The factors that explained the most variation in cumulative rates of SARS-CoV-2 infection between Jan 1, 2020, and Sept 30, 2021, included the proportion of the population living below 100 m (5·4% [4·0–7·9] of variation), GDP per capita (4·2% [1·8–6·6] of variation), and the proportion of infections attributable to seasonality (2·1% [95% uncertainty interval 1·7–2·7] of variation). Most cross-country variation in cumulative infection rates could not be explained. The factors that explained the most variation in COVID-19 IFR over the same period were the age profile of the country (46·7% [18·4–67·6] of variation), GDP per capita (3·1% [0·3–8·6] of variation), and national mean BMI (1·1% [0·2–2·6] of variation). 44·4% (29·2–61·7) of cross-national variation in IFR could not be explained. Pandemic-preparedness indices, which aim to measure health security capacity, were not meaningfully associated with standardised infection rates or IFRs. Measures of trust in the government and interpersonal trust, as well as less government corruption, had larger, statistically significant associations with lower standardised infection rates. High levels of government and interpersonal trust, as well as less government corruption, were also associated with higher COVID-19 vaccine coverage among middle-income and high-income countries where vaccine availability was more widespread, and lower corruption was associated with greater reductions in mobility. If these modelled associations were to be causal, an increase in trust of governments such that all countries had societies that attained at least the amount of trust in government or interpersonal trust measured in Denmark, which is in the 75th percentile across these spectrums, might have reduced global infections by 12·9% (5·7–17·8) for government trust and 40·3% (24·3–51·4) for interpersonal trust. Similarly, if all countries had a national BMI equal to or less than that of the 25th percentile, our analysis suggests global standardised IFR would be reduced by 11·1%.

**Interpretation:**

Efforts to improve pandemic preparedness and response for the next pandemic might benefit from greater investment in risk communication and community engagement strategies to boost the confidence that individuals have in public health guidance. Our results suggest that increasing health promotion for key modifiable risks is associated with a reduction of fatalities in such a scenario.

**Funding:**

Bill & Melinda Gates Foundation, J Stanton, T Gillespie, J and E Nordstrom, and Bloomberg Philanthropies.

**Research in context**

**Evidence before this study**

Responsive policies such as physical distancing and mask mandates were important in shaping outcomes during the COVID-19 pandemic. Yet, the conditions associated with cross-country variation in infection and fatality rates during the COVID-19 pandemic are not well understood. In the aftermath of the 2013–16 Ebola epidemic in west Africa, WHO launched a voluntary Joint External Evaluation (JEE) process to track adoption of core capacities required under the 2005 International Health Regulations and to assess national capacity to prevent, detect, and respond to disease with potential for pandemic spread. WHO's April 2021 interim assessment did not find JEE scores from the 100 countries that had conducted voluntary assessments to be correlated with COVID-19 outcomes, although such metrics were designed as benchmarking exercises for National Action Plans rather than cross-country comparators. Preliminary analysis of COVID-19 outcomes in relation to other health-system capacity indices, such as the Global Health Security Index and the index of effective coverage of universal health coverage produced by the Global Burden of Diseases, Injuries, and Risk Factors Study (GBD) have also been found not to be predictive of COVID-19 outcomes. Other exploratory research on COVID-19 outcomes has had a regional focus or has focused on a small number of country experiences.

**Added value of this study**

We analysed measures of pandemic preparedness. 12 indicators of preparedness and response and seven indicators of health-system capacity were considered, in addition to ten other demographic, social, and political conditions that previous research suggests might be relevant. Associations with both incidence and mortality from SARS-CoV-2 infections were investigated. We controlled for demographic, biological, economic, and environmental variables associated with COVID-19 outcomes, including population age structure and environmental seasonality, population density, national income, and population health risks, to identify contextual factors subject to policy control. This research considerably expands on the scope of previous research by investigating correlates of pandemic preparedness and mitigation in 177 countries between Jan 1, 2020, and Sept 30, 2021, and includes inputs that have been adjusted for problems associated with under-reporting of COVID-19 outcomes. This expanded scope was possible because of inputs from COVID-19 research produced by the Institute for Health Metrics and Evaluation and mortality and population estimates generated by GBD.

**Implications of all the available evidence**

The existing metrics for health-system capacity and national pandemic preparedness and response have been poor predictors of pandemic outcomes, suggesting other areas might merit greater weight in future preparedness efforts. Not all of the correlates that account for some variation in infections per capita and infection-to-fatality ratios, such as age structure, altitude at which a population lives, and environmental seasonality, are easy for policy makers to control. Yet, other factors are within the policy realm, including preventive health measures focused on population health fundamentals: encouraging healthy bodyweight and reducing smoking might be helpful in averting morbidity and mortality in future pandemic scenarios. Moreover, the level of trust is something that a government can prepare for and earn in a crisis, and our analysis suggests doing so may be crucial to mount a more effective response to future pandemic threats. Large unexplained variation in differences in SARS-CoV-2 infections across countries speaks to the importance of further research in this area.

## Introduction

While the world remains in the grip of COVID-19, crucial efforts are already underway to begin learning from the pandemic response.^1^, ^2^, ^3^ Policy makers have begun developing new global and national pandemic preparedness proposals to ensure the world is better prepared when the next deadly and fast-spreading novel pathogen emerges.^4^, ^5^, ^6^, ^7^, ^8^ Policy responses such as mask mandates and physical-distancing measures have been key to shaping outcomes.^9^, ^10^, ^11^ However, identifying the contextual factors associated with reduced infection and fatality rates is important to guide the long-term path to addressing future threats.

COVID-19 has been called an “epidemiological mystery”.^12^ Reported incidence and mortality from SARS-CoV-2 have not followed the pattern of many other communicable diseases; wealthier countries with more health-care resources have had a greater burden from COVID-19 than have low-income countries with fewer health-care resources. Upper-middle-income and high-income countries have 48% of the global population but 53% of the total estimated excess mortality-adjusted cumulative deaths from COVID-19 as of Sept 30, 2021, despite having much higher COVID-19 vaccination rates since December, 2020, compared with those in low-income and lower-middle-income countries.^13^, ^14^ Also, national cumulative mortality rates have varied dramatically, even among countries within close geographical proximity. Bulgaria, Namibia, and Bolivia have COVID-19 mortality rates greater than 4 deaths per 1000 people, whereas geographical neighbours Turkey, Angola, and Colombia, respectively, have fewer than half as many deaths per capita, with mortality rates at or below 2 per 1000. Moreover, countries that experts believed before the pandemic to be most prepared to mitigate the effects of a pandemic have not been the most successful at doing so.^1^ A preliminary analysis in June, 2020, examined the Global Health Security (GHS) Index, the WHO Joint External Evaluation (JEE), and a measure of universal health coverage and found no connection between those capacity measures and COVID-19 deaths, even when accounting for differences in population age structure.^15^ The report of the Independent Oversight and Advisory Committee for the WHO Health Emergencies Program in May, 2021, also did not find evidence of a relationship between JEE scores and COVID-19 outcomes.^1^

Some previous research has attempted to explain variation in country experiences controlling SARS-CoV-2 infections and averting mortality. To date, most of these analyses have either been commentaries on why preparedness metrics are useful despite not being predictive of COVID-19 pandemic outcomes, or have focused on specific regions,^16^ used somewhat limited correlational and descriptive analyses without controlling for known key determinents,^17^, ^18^ focused exclusively on cumulative deaths,^19^ or had small sample sizes and missing data.^20^ One analysis investigated some political, social, and governmental correlates with cumulative deaths per capita,^21^ and another found a relationship between trust in government and reduced death rates.^22^ Our analysis builds on this research by incorporating results from the Institute for Health Metrics and Evaluation (IHME) on estimated infections built from hospitalisations, reported cases, and deaths accounting for excess mortality due to the COVID-19 pandemic,^13^, ^14^ and additional covariates of interest such as metrics of health system and pandemic preparedness and response capacity. Additionally, we controlled for key covariates associated with age structure of the population and environmental seasonality, among other factors.^23^, ^24^ Without controlling for these factors, an analysis risks confounding from other deterministic drivers that are outside the control of policy makers. Also, we differentiated between infection rates and infection-fatality ratios (IFRs) to assess the differences in prevention and treatment of COVID-19. Finally, we incorporated subnational data where available.

The aim of this research was to complete an exploratory analysis of potential correlates of COVID-19 prevention and treatment across 177 countries and territories. We investigated these correlates in relation to both SARS-CoV-2 infections and IFRs to disentangle the factors that prevented the spread of the virus from the health-system factors that prevented death from disease. We controlled for known factors of SARS-CoV-2 infection and mortality that are generally considered outside the control of policy makers (such as altitude, age profile, and seasonality of the disease) and explored associations with 28 factors that policy makers can control. Variables explored were associated with pandemic preparedness indices; health-system capacity indicators; governance variables; and measures of economic inequality and societies' trust in their government, science, and their communities.

## Methods

### Overview

In this research, the outcomes of interest were infections per capita and IFRs. Both were calculated from estimates produced by IHME's ongoing COVID-19 project.^23^, ^24^ This research was done in three stages. In stage 1, we standardised the national infection rates and IFRs by estimating what the infection rate and IFR would be if each country had the global mean value of key known drivers of infection and IFR. This process included adjusting national infection rates for environmental seasonality, altitude, and income, among other factors, and standardising IFRs to the global age distribution and the prevalence of competing risks. In stage 2, we measured the cross-country association of these standardised infection rates and IFRs against health-system policy variables, such as measures of pandemic preparedness, health-system capacity, governance factors, and several measures of social and governmental trust, to identify which of these policy factors, if any, were associated with fewer infections and lower IFR. In stage 3, we investigated how reduction in mobility and vaccine coverage might be pathways for more distal policy variables to affect infection rates and IFR. For stages 1 and 2, we assessed two time periods. To assess the full span of the pandemic (until present), we assessed cumulative infection and IFR for Jan 1, 2020, until Sept 30, 2021. As a sensitivity analysis, we also assessed the time period before vaccines and disease variants were known to have spread, Jan 1, 2020, until Oct 15, 2020. All analyses were done using R (version 4.0.3).^25^

This study complies with the Guidelines for Accurate and Transparent Health Estimates Reporting (GATHER) recommendations (appendix p 5).^26^ Code used to produce this analysis is available online.

### COVID-19 infection and mortality estimates

Daily estimated infections and death counts were extracted from the IHME modelling database. These estimates span from Jan 1, 2020, to Sept 30, 2021, and exist for 177 countries and 181 subnational locations.^24^ To estimate the number of COVID-19 deaths, IHME extracted data from the Johns Hopkins University Center for Systems Science and Engineering COVID-19 database, supplemented these data with additional data from national and subnational ministries and departments of health, and adjusted them to correct for missing data and reporting lags. The resulting mortality rates were then adjusted for under-reporting on the basis of the ratio of excess deaths attributable to COVID-19 versus reported deaths, a ratio that was modelled using spatial correlation and additional covariates.^24^ To estimate the number of SARS-CoV-2 infections, IHME estimated infections from the number of deaths, hospital census, and reported cases occurring in each location, again extracted from the Johns Hopkins COVID-19 database and adjusted for missing data and reporting lags. The infections estimate was based on IFR, infection to hospitalisation ratios, and infection to detection ratios, respectively, estimated for each population. Ratio observations were derived by matching the parameter (eg, deaths) to the number of infections occurring in the population according to seroprevalence surveys, the results of which have been adjusted for waning sensitivity of antibody tests and other known biases. The IFR, infection to hospitalisation ratio, and infection to detection ratio were then modelled as a function of covariates to obtain predictions for all locations and days. Underlying data uncertainty and modelling uncertainty were propagated at each stage and incorporated into the quantification of the estimates' uncertainty. Full details of the modelling approaches are provided elsewhere.^13^, ^14^, ^24^, ^27^

For this study, cumulative infections were calculated by summing up the total estimated daily infections for each national or subnational location over the entire time period (and also for the shorter time period, Jan 1, 2020, to Oct 15, 2020), and were divided by the 2019 estimated population in each location to get the cumulative infections per capita.^28^ The IFRs were calculated by applying a 9-day lag to our daily infections to account for the delay between infection and death, calculating the sum of infections and deaths, and then dividing the cumulative deaths over the cumulative lagged infections.

### Variable selection

In stage 1, we included demographic, biological, comorbid, economic, and environmental factors known or believed to have influenced infection rates or IFR. Most of these background variables are generally not factors subject to direct policy-maker control, such as population density, gross domestic product (GDP), altitude, and seasonality—factors that might increase transmission—and age, age-standardised chronic obstructive pulmonary disease (COPD) prevalence, and age-standardised cancer prevalence—factors that might increase morbidity or mortality from infection—and previous exposure to coronaviruses, a factor that might influence both subsequent transmission probability and mortality outcomes. A few biological and environmental factors considered at stage 1 are related to health and are policy-amenable, such as body-mass index (BMI), smoking, and air pollution, which might cause increased IFR. We used a theoretical, rather than empirical, approach for including these covariates, assessed for multicollinearity (appendix p 12), and generated standardised infection rates and IFRs that hold these values constant across countries.

In stage 2, we sought to test associations between the standardised infection rates and standardised IFRs and key measures of pandemic preparedness, health-care capacity, and government effectiveness and social conditions that are subject to policy-maker control (table 1). The pandemic preparedness measures included were the JEE and GHS Index, as well as their subcomponents. The health-care capacity and spending measures included the effective coverage index of universal health coverage (UHC) and two UHC index subcomponents (non-communicable diseases and communicable, maternal, and neonatal disease), Healthcare Access and Quality (HAQ) Index, hospital beds per capita, governmental health expenditure per capita, and health spending per capita. For governance and social measures, we included factors that might affect government capacity, priorities, and responsiveness in a pandemic (such as electoral democracy, government effectiveness, populism, state fragility, and corruption), as well as social factors that might affect the willingness of a population to comply with government or health mandates (income inequality and trust in the government, science, and other members of that population).

**Table 1:** Covariates used in stage 1 and 2 analyses

	**Units**	**Temporal coverage**	**Spatial coverage**	**Data source**	**Notes**
**Stage 1 covariates**					
Pneumonia relative risk	Relative risk of death from pneumonia divided by the average risk of death from pneumonia	2013–19	National and subnational	Modelling COVID-19 scenarios for the USA^34^	Varies weekly
Age	Age structure of the population (5-year age bins)	2020	National and subnational	GBD 2019	..
Altitude	Population living below 100 m (%)	2015	National and subnational	GBD 2019	..
Population density	Population living above 1000 people per km^2^ (%)	2020	National and subnational	Modelling COVID-19 scenarios for the USA^34^	..
Air pollution	PM_2·5_air pollution concentration (mg/m^3^)	2019	National and subnational	GBD 2019	..
Smoking prevalence	Age-standardised tobacco smoking prevalence	2019	National and subnational	GBD 2019	..
Cancer prevalence	Age-standardised cancer prevalence	2019	National and subnational	GBD 2019	..
COPD prevalence	Age-standardised COPD prevalence	2019	National and subnational	GBD 2019	..
Bats	Average number of betacoronavirus-host bat species in a given location	2021 bats and ranges	National only	IUCN and Verena Consortium	See appendix (p 44) for more details of methodology
Gross domestic product per capita	2019 US$	2019	National and subnational	GBD 2019	..
BMI	Population-adjusted BMI	2019	National and subnational	GBD 2019	..
**Stage 2 covariates**					
JEE components and Prevent Epidemics' Preparedness overall score	Index	2016–21	National only	WHO and Prevent Epidemics	Only places that have completed a JEE; overall score is a summary variable of JEE components created by Prevent Epidemics
Global Health Security Index components and overall score	Index	2019	National only	Global Health Security Index 2019	Weighted average of the other components
Universal health coverage	Index	2019	Nationals	GBD 2019, Measuring Universal Health Coverage	Included two subcomponents: communicable and non-communicable
Healthcare Accessibility and Quality Index	Index	2019	National only	GBD 2019	..
Government health spending per capita	2019 US$	2020	National only	Global Burden of Disease Health Financing Collaborator Network	Mean value
Beds per capita	Number of hospital beds per capita before start of the pandemic	2019	National and subnational	GBD 2019	..
Health spending per capita	2019 US$	2020	National only	Global Burden of Disease Health Financing Collaborator Network	Mean value
Government corruption (PCA)	Index	..	National only	Transparency International; Varieties of Democracy Institute, Version 10^21^	Principal components analysis of V-Dem Public sector corruption and the Transparency International's Corruptions Perceptions Index; see appendix (p 45) for more details of methodology
Electoral populism	Populism-based campaign run	..	National only	Populism in Power & Bosancinau^21^	Whether a democratically elected head of government ran a populist campaign
Government effectiveness	Index	..	National only	World Bank Indicators and Bosancinau^21^	Perceived quality of public services, its provision, and providers
State fragility	Index	..	National only	State Fragility Index and Bosancinau^21^	Incapacity to provide essential public goods and services and cope with shocks
Electoral democracy index	Index	2020	National only	Varieties of Democracy Institute, Version 11	Aggregate indicator combining free and fair elections, free association, freedom of expression and access to alternative information, suffrage, and elected officials
Interpersonal trust	Trust in other people	2017–21	National only	World values survey wave 7	Trust coded as those who answered “most people can be trusted on Q57”
Trust in science	..	2018	National only	Wellcome Global Monitor Survey	Those who answered “a lot” to trusting science
Trust in government (PCA)	Index	2017–21	National only	World Values Survey Wave 7; Gallup World Poll	Principal components analysis of Gallup's Politics and Government variable Confidence in National government and World Values Survey (Wave 7) question on confidence in government; see appendix (p 45) for more details of methodology
Gini	Gini index	..	National only	SWIID version 8.2 and Bosancinau^21^	..

Further references for data sources given in the appendix (p 4). BMI=body-mass index. GBD=Global Burden of Diseases, Injuries, and Risk Factors Study. COPD=chronic obstructive pulmonary disease. JEE=Joint External Evaluation. PCA=principal component analysis. SWIID=Standardized World Income Inequality Database.

### Stage 1: standardising infection rates and IFRs

To improve comparability across countries, we used regression analyses to standardise the effects of COVID-19 determinants that were not directly related to our research questions. For infections, we regressed infection rates on the time-varying relative risk of pneumonia, and then predicted infection rates holding all countries constant at the global mean, generating seasonally adjusted infection rates. We then used a multivariate generalised linear regression to model the association between seasonality-adjusted infections per capita and GDP per capita, a proxy for previous exposure to betacoronavirus-host bat species (appendix p 44), proportion of the population living below 100 m (a factor meant to capture variability in incidence of pneumonia and other lower respiratory infections that vary by altitude^29^, ^30^, ^31^), and population density. We produced standardised infection rates by estimating the seasonally adjusted infection rates for each location had each had the global mean of each of these factors.

For IFR, we used indirect age standardisation to remove the effects of different age profiles across locations. We then used a multivariate regression model to assess the relationships between the age-standardised IFR and national income per capita, a proxy for previous exposure to betacoronavirus-host bat species, population density, mean BMI, age-standardised COPD prevalence, age-standardised cancer prevalence, air pollution, and smoking prevalence. Among five disease prevalences believed to be related to IFR^32^ (COPD, cancer, diabetes [of any type], cardiovascular disease, and chronic kidney disease), the age-standardised rates of chronic kidney and diabetes were correlated, and cardiovascular disease and diabetes were correlated with BMI, leading to multicollinearity. Consequently, age-standardised prevalence of chronic kidney disease, age-standardised cardiovascular disease, and age-standardised diabetes (the factors with the largest variance inflation factors) were removed from the model.

All models were linear regressions, with dependent and independent variables natural-log-transformed adding 5% of the median value for covariates with estimated zeros. To account for within-country correlations and avoid any one country's estimate from unduly impacting our parameter estimates, we down-weighted subnational locations corresponding to their proportion of the country's population. To estimate the fraction of variance explained by each covariate, we did a Shapley decomposition of the *r*^2^. Variation accounted for by adjusting for seasonality or age was calculated by comparing the sum of squares of the standardised values (cumulative infections per capita and IFRs) to their respective raw values. Additionally, we used regressions estimated in stage 1 to conduct counterfactual analyses. These analyses are presented in table 2 and denote reductions in IFR had risk factors (ie, BMI) been at the 25th percentile across countries, and reductions in infections had trust variables (ie, government trust and interpersonal trust) been at the 75th percentile across countries.

**Table 2:** Factors associated with variation in cross-country cumulative infections per capita, IFR, and hypothetical levels of trust and prevalence of risk factors

	**Variation in infections per capita explained by each factor, % (95% UI)**	**Variation in IFR explained by each factor, % (95% UI)**	**Reduction in global infections each country's level of trust had exceeded 75th percentile across countries, % (95% UI)**	**Reduction in global IFR if each county's mean BMI was less than the 25th percentile across all countries, % (95% UI)**
Seasonality	2·1% (1·7–2·7)*	..	..	..
Age structure	..	46·7% (18·1–67·6)*	..	..
GDP per capita	4·2% (1·8–6·6)*	3·1% (0·3–8·6)*	..	..
Population density	1·8% (0·8–3·2)	1·7% (0·3–5·6)	..	..
Altitude	5·4% (4·0–7·9)*	..	..	..
Pre-exposure to betacoronavirus	2·1% (1·1–3·1)	0·7% (0·1–2·1)	..	..
Body-mass index	..	1·1% (0·2–2·6)*	..	11·1% (2·1–20·6)*
Smoking prevalence	..	0·3% (0·1–3)	..	..
Air pollution	..	0·3% (0·1–2·1)	..	..
COPD prevalence	..	0·2% (0·0–0·7)	..	..
Cancer prevalence	..	1·6% (0·1–4·8)	..	..
Trust in government†	7·4% (5·4–9·6)*	..	12·9% (5·7–17·8)*	..
Interpersonal trust†	16·5% (12·3–19·5)*	..	40·3% (24·3–51·4)*	..
Unexplained variation	60·6% (55·6–65·2)	44·4% (29·2–61·7)	..	..

BMI=body-mass index. COPD=chronic obstructive pulmonary disease. IFR=infection-fatality ratio. UI=uncertainty interval.

* Estimated parameters that are statistically different from zero.

† These covariates are assumed to be independent from each other and all other covariates. Further, a few countries had incomplete reporting of these covariates. Corresponding figures reflect those countries where the respective covariate was present.

### Stage 2: exploring health care, governance, and social associations with standardised COVID-19 outcomes

Standardised infection rates and standardised IFRs from stage 1 (measured for all 177 countries) were used as the dependent variable in stage 2. We regressed these on the 28 health-care, governance, and social indicators previously described and which are outlined in table 1.

For comparisons, all covariates were centred around 0 and scaled to have an SD of 1. Because these indicators are highly correlated with other indicators, and some have a great deal of missingness (appendix pp 8–40), we used bivariate linear regression models to assess each association independent of all the other indicators. To address the issue of testing multiple hypotheses, we adjusted our p value threshold using a Bonferroni correction for each of our stage 2 covariates (n=28) and a desired α of 0·05, resulting in a significance cutoff of 0·0018. For trust in the government and government corruption, multiple data sources existed with varying degrees of variable completeness and coverage by location, so we conducted a principal component analysis (PCA) and extracted the first component to create a summary indicator. We used a PCA method (missMDA package in R)^33^ that imputed values for countries with missing information (appendix p 45). To estimate the fraction of variance explained by each indicator, we noted the sum of squares explained by each factor and combined each value with the raw sum of squares of cumulative infections per capita or of IFR.

### Stage 3: the association between key factors and intermediate health outputs

To explore two potential pathways connecting the governance and social factors identified as statistically associated with COVID-19 outcomes in stage 2, we assessed the relationship between these variables—interpersonal trust, government trust, and government corruption—and the most extreme country-specific reduction in mobility observed at any point in 2020 or 2021, relative to a pre-pandemic baseline that was based on a composite metric extracted from smartphone data,^34^ and the maximum achieved vaccine coverage (at least one dose) as of Sept 30, 2021. Given the lack of access to vaccinations and vaccine supplies in many low-income and lower-middle-income countries during our study period, the analysis of vaccine coverage was only on upper-middle-income and high-income countries.

### Uncertainty and sensitivity analyses

To capture uncertainty associated with the input data and uncertainty associated with our linear models, we completed our analysis 100 times independently, each on a separate draw produced previously by IHME for estimating infection and IFR uncertainty.^24^, ^35^ Additionally, for each draw and each linear regression we took a random sample draw from the estimated variance-covariance matrix to incorporate model uncertainty. Similarly, the PCA-based summary indicators were completed 100 times to capture uncertainty from the imputation process. Here, we report the mean of the 100 estimates, with uncertainty intervals (UIs) spanning for the 2·5th and 97·5th percentiles of the 100 estimates.

To assess the effect of our modelling choices, we did sensitivity analyses using centred and scaled data rather than log-transformed covariates in stage 1, and adding 1% of the median for covariates with values of 0, rather than 5%. To assess the effect of imperfect input data, we did sensitivity analyses using only national and subnational locations where we had seroprevalence survey data (n=303; appendix p 53), and did our analysis without incorporating IHME's technique for adjusting for within-country excess mortality. To assess the role that novel variants and vaccine coverage might have on our analysis, we did a sensitivity analysis including variant spread as a stage 1 covariate and ran our analyses using a subset of the data from the pre-vaccine, pre-variant era, Jan 1, 2020, to Oct 15, 2020.

### Role of the funding source

Funding was provided by the Bill & Melinda Gates Foundation, Bloomberg Philanthropies, J Stanton, T Gillespie, and J and E Nordstrom. The funders of the study had no role in the study design, data collection, data analysis, data interpretation, or the writing of the report. Members of the core research team for this topic area had full access to the underlying data used to generate estimates presented in this paper. All other authors had access, and reviewed, estimates as part of the research evaluation process, which includes additional stages of formal review.

## Results

**Figure 1:**
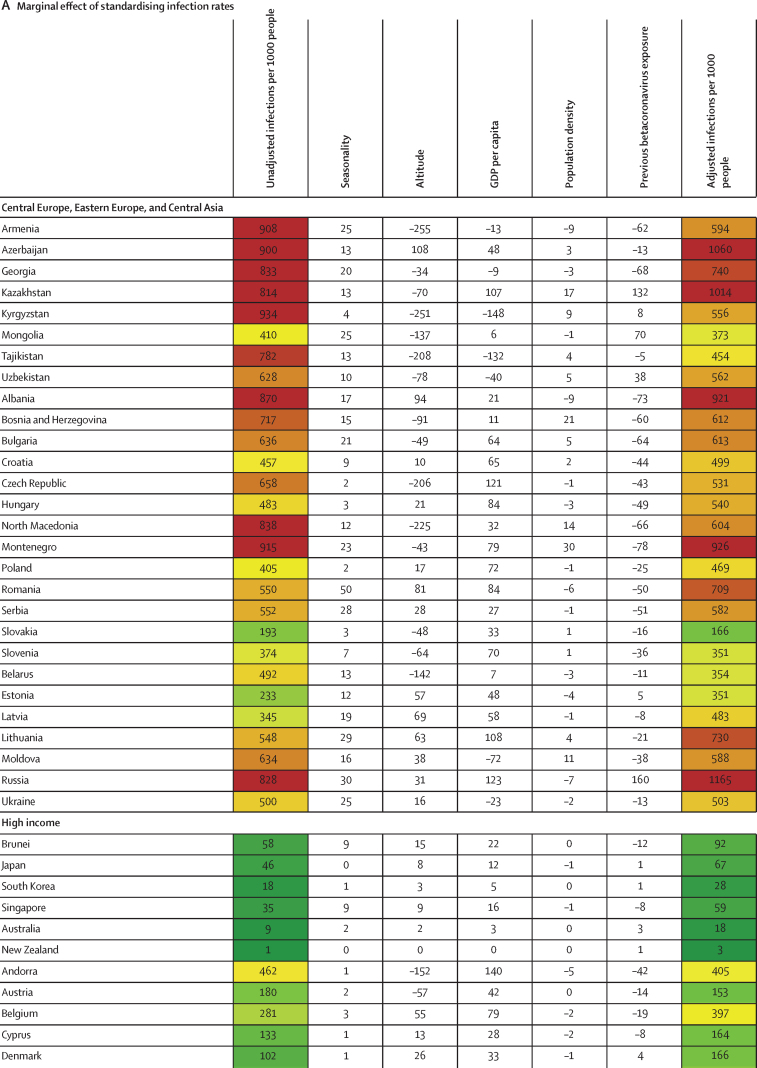

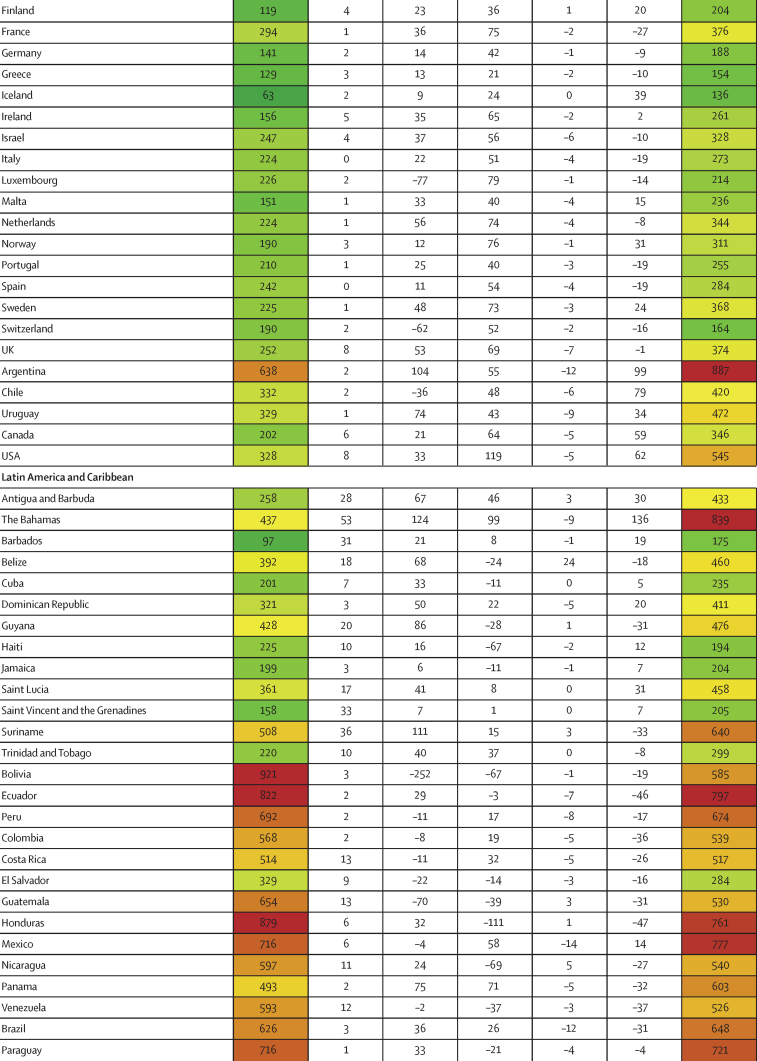

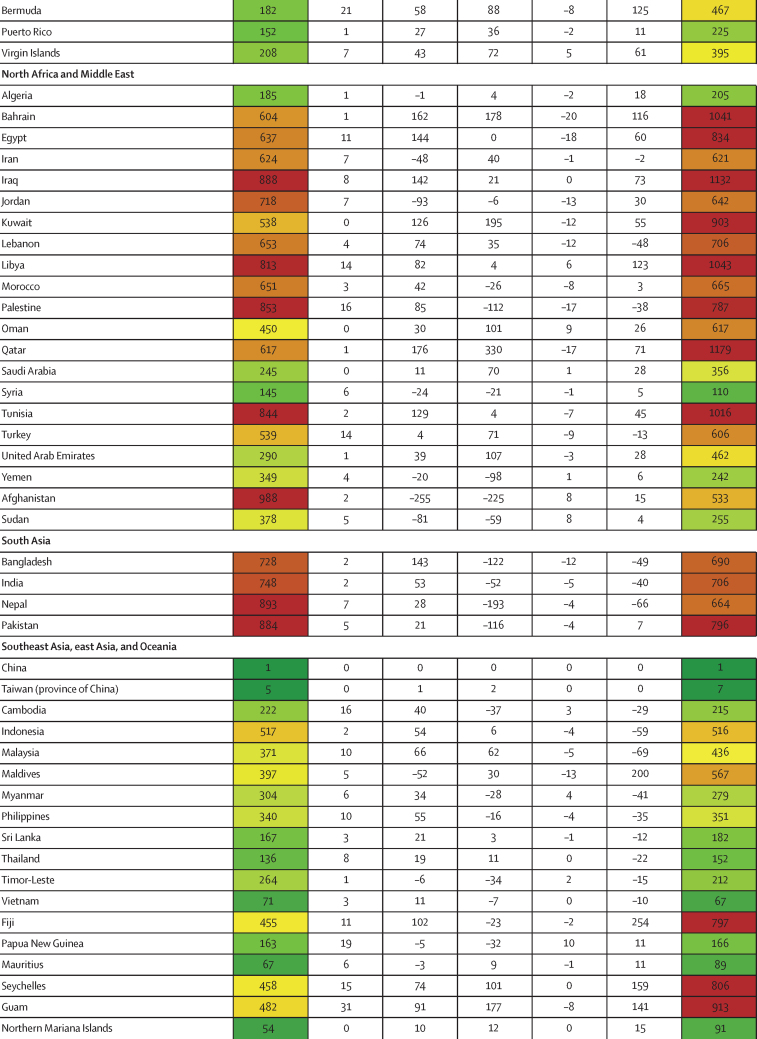

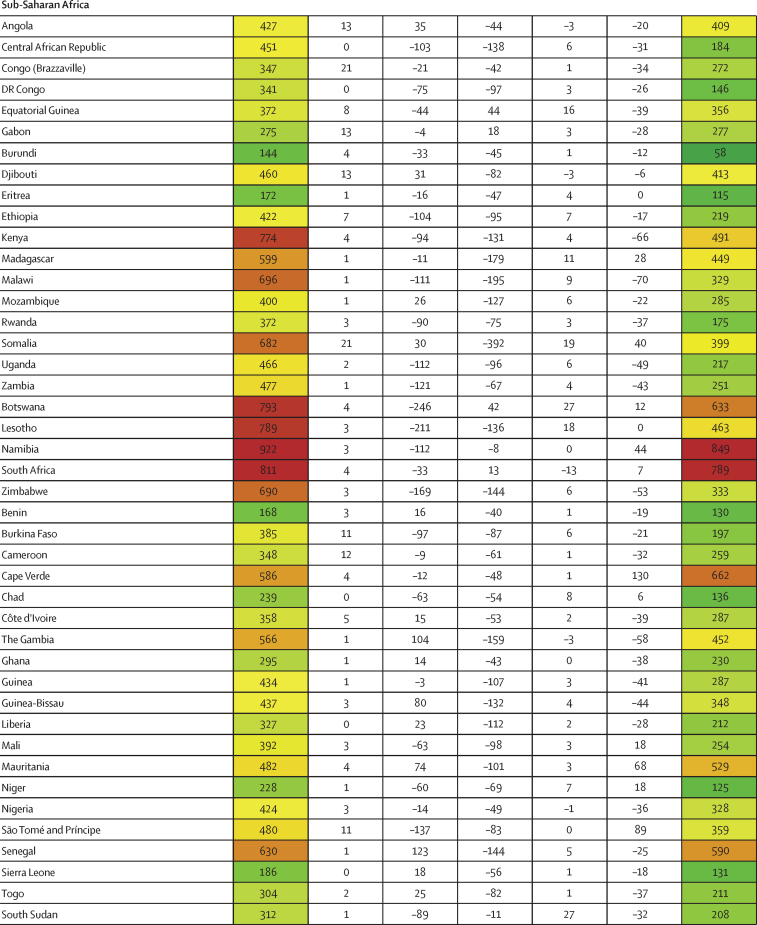

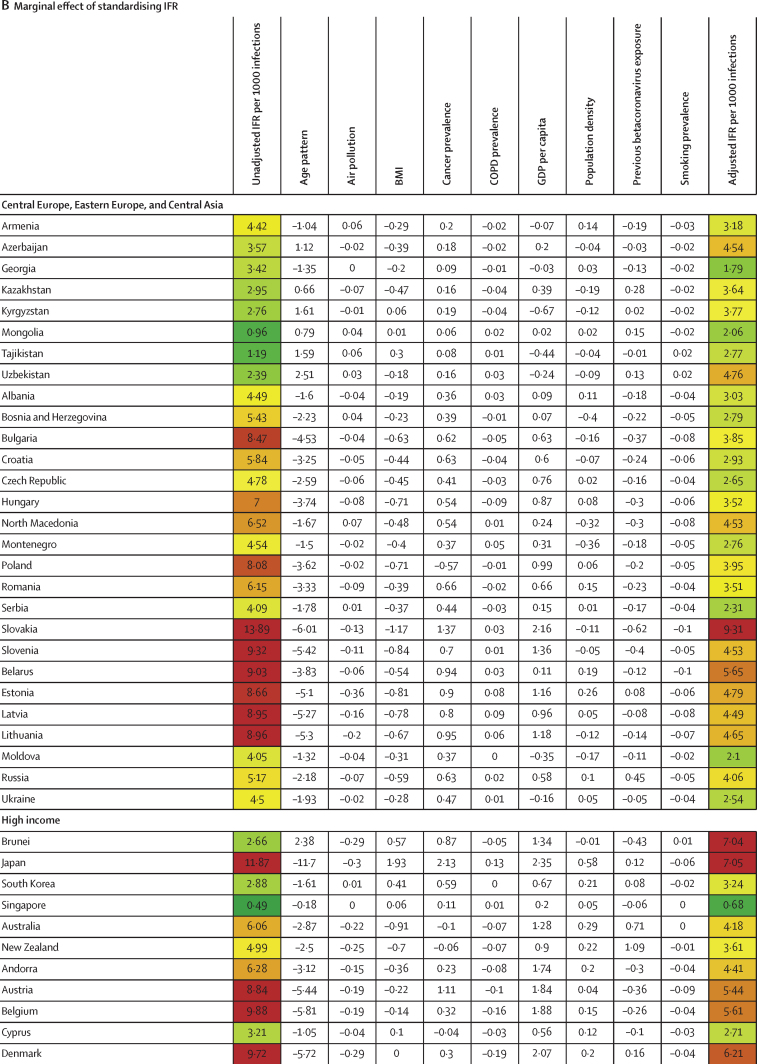

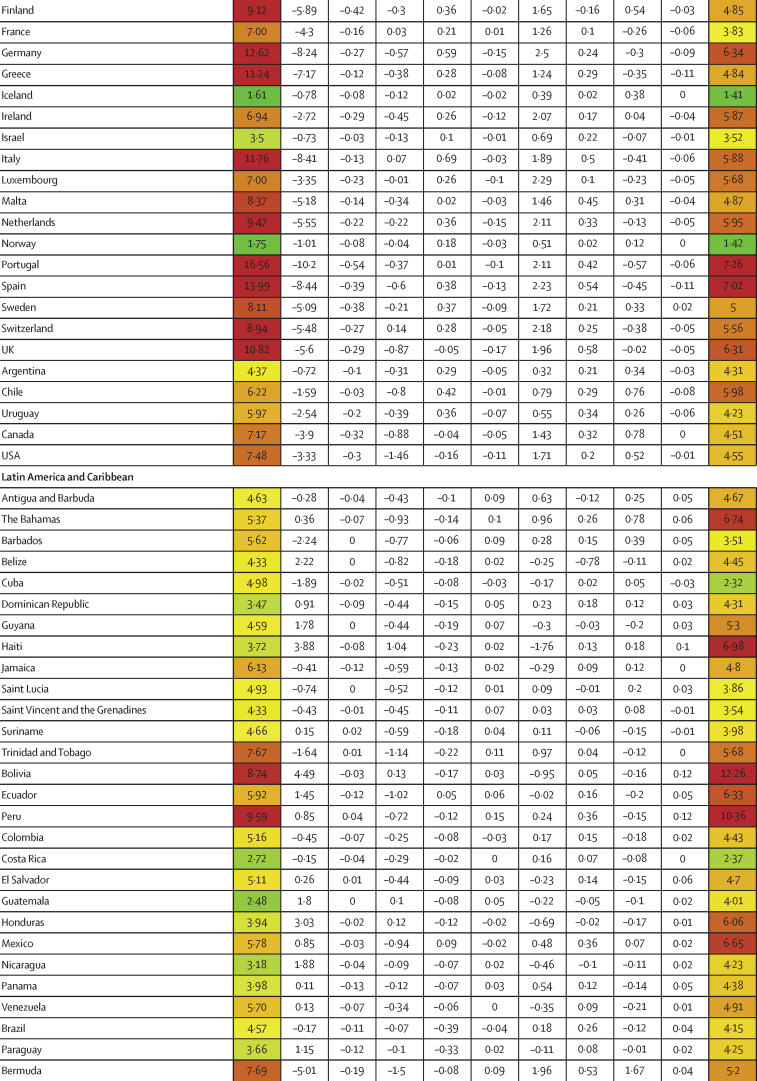

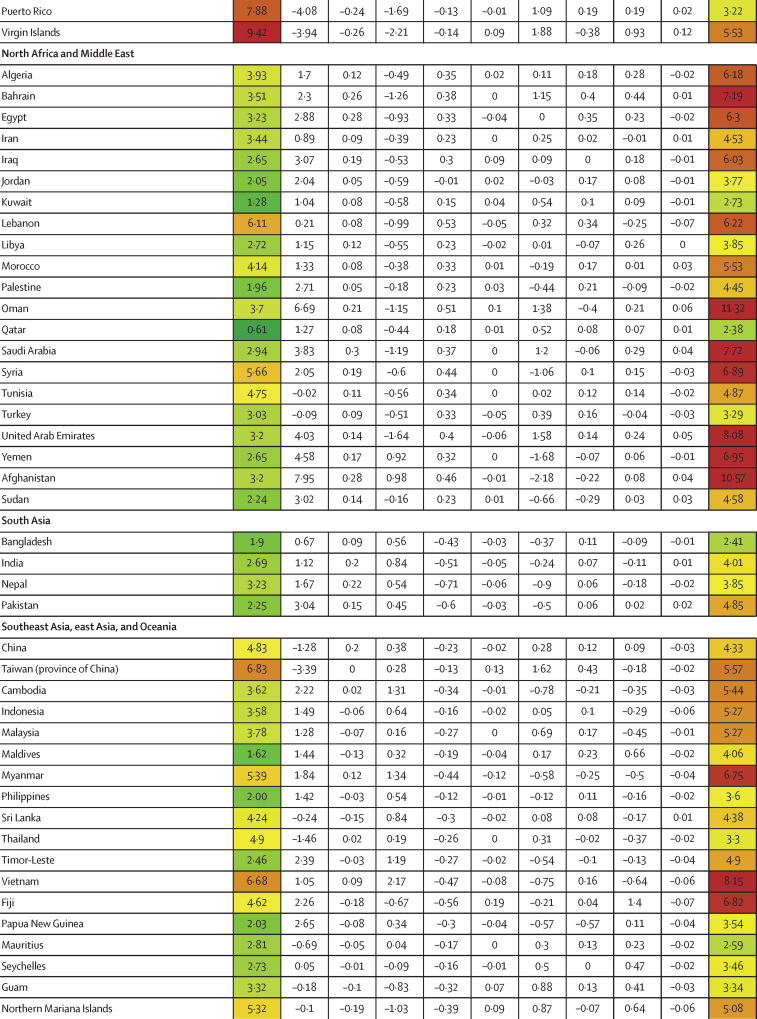

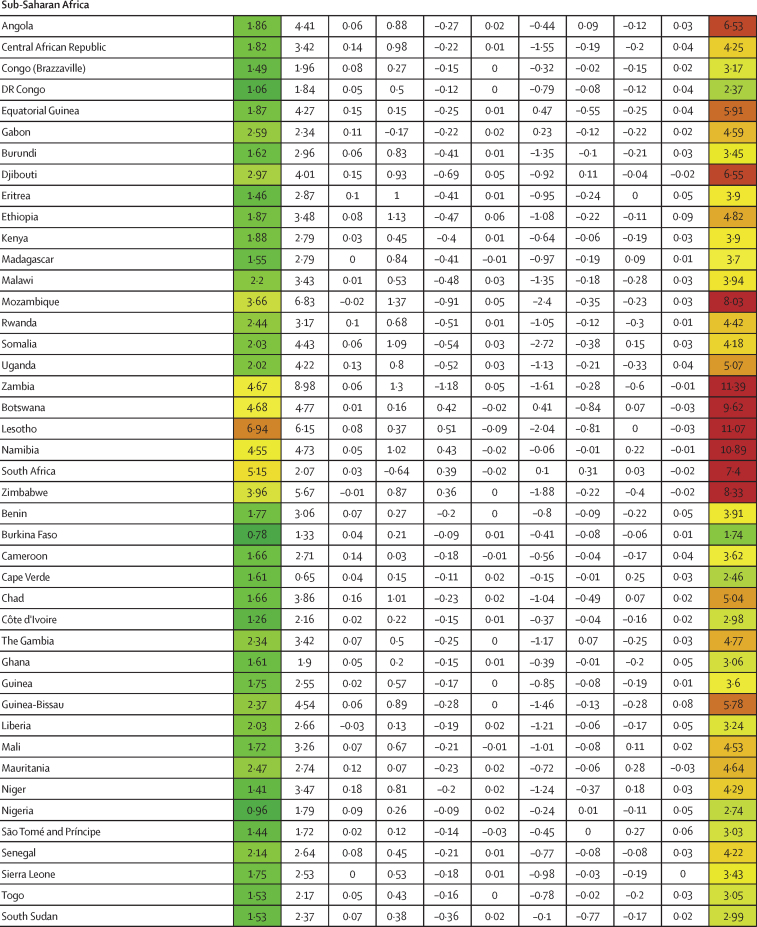
Decomposition of difference in standardised cumulative SARS-CoV-2 infections per capita and IFR (A) Decomposition of the difference between cumulative and standardised cumulative SARS-CoV-2 infections per capita. The first column represents the number of unadjusted infections per capita, and each subsequent column represents the change in adjusted cumulative infections per capita that can be accounted for by seasonality, altitude, GDP per capita, population density, and a proxy for pre-exposure to betacoronavirus; the last column represents the adjusted infections per capita. The infections per capita metrics are colour-coded based on their severity relative to all other countries, with red representing higher cumulative infections per capita and green representing lower cumulative infections per capita (unadjusted and adjusted). (B) Decomposition of the difference between IFR and standardised IFR. The first column represents the raw IFR, and each subsequent column represents the proportion of IFR that can be accounted for by age structure, air pollution, BMI, cancer prevalence, COPD prevalence, GDP per capita, population density, a proxy for pre-exposure to betacoronavirus, and smoking prevalence; the last column represents the adjusted IFR. The IFR metrics are colour-coded based on their severity relative to all other countries, with red representing higher IFR and green representing IFR (raw and adjusted). BMI=body-mass index. COPD=chronic obstructive pulmonary disease. GDP=gross domestic product. IFR=infection-fatality ratio.

Between Jan 1, 2020, and Sept 30, 2021, we found substantial cross-country variation in SARS-CoV-2 infection rates (figure 1A). Although the global infection rate was 433 per 1000 population (95% UI 393–493), country-specific estimates ranged from 1 per 1000 population (0–1) in China to 988 per 1000 population (507–1111) in Afghanistan. Excess relative risk of infection associated with seasonality explained 2·1% (1·7–2·7) of the variation in cross-country daily infection rates (table 2). Countries such as Romania, the Bahamas, and Suriname were most positively affected by excess risk associated with seasonality (figure 1A). A further 5·4% (4·0–7·9) of the cross-country variation was explained by controlling for population living below 100 m in altitude, followed by GDP per capita (4·2% [1·8–6·6] of variation; table 2). Population density and our proxy measure of previous exposure to betacoronaviruses from bat hosts were not significantly associated with infection (table 2). Most cross-country variation (60·6% [55·6–65·2]) in cumulative infection rates could not be explained by the covariates presented to the analysis.

Similar to infection rates, COVID-19 IFRs varied dramatically across countries during the first 21 months of the pandemic (January, 2020, to September, 2021; figure 1B). Although the global IFR was 3·4 (95% UI 2·4–4·8) per 1000 infections, country-specific estimates ranged from 0·49 (0·34–0·64) in Singapore to 16·56 (9·35–26·81) in Portugal. Age was a clear contributor to this cross-country variation, explaining 46·7% (18·1–67·6) of cross-country variation. The countries that saw the largest increases in IFR due to age (ie, the countries with older populations, and saw decreases in IFR when age standardised) were Japan, Portugal, and Spain (figure 1B). The countries with the largest decreases in IFR due to young age profile (and therefore saw an increase in IFR when age standardised) were Afghanistan, Mozambique, and Zambia (figure 1B). After adjusting for age, variation in BMI was significantly associated with age-standardised IFR, accounting for 1·1% (0·2–2·6), as was GDP per capita, accounting for 3·1% (0·3–8·6). 44·4% (29·2–61·7) of cross-national variation in IFR could not be explained (table 2). A 10% increase in BMI was associated with an increase in age-standardised IFR of 17·4% (6·5–32·0; p<0·0001). If these associations are causal and all countries had national BMI that was equal to or less than that of the global 25th percentile, these associations suggest global standardised IFR could be reduced by 11·1% (2·1–20·6).

**Figure 2:**
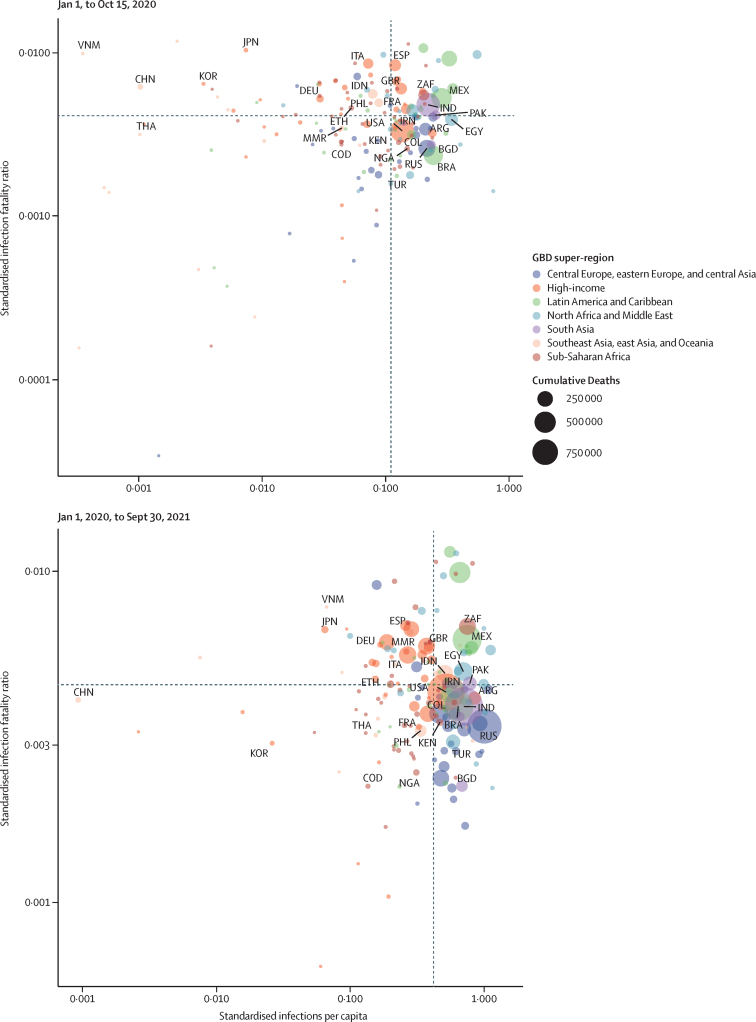
Standardised infections per capita and standardised infection-fatality ratios The top panel shows the relationship between adjusted infections per capita and adjusted infection-fatality ratios from Jan 1, 2020, to Sept 30, 2021. The bottom panel shows the relationship between adjusted infections per capita and adjusted infection-fatality ratios from Jan 1, 2020, to Oct 15, 2020. The size of each circle represents the magnitude of cumulative deaths. ARG=Argentina. BGD=Bangladesh. BRA=Brazil. CHN=China. COD=Democratic Republic of the Congo. COL=Colombia. DEU=Germany. EGY=Egypt. ESP=Spain. ETH=Ethiopia. FRA=France. GBD=Global Burden of Diseases, Injuries, and Risk Factors Study. GBR=UK. IDN=Indonesia. IND=India. IRN=Iran. ITA=Italy. JPN=Japan. KEN=Kenya. KOR=South Korea. MEX=Mexico. MMR=Myanmar. NGA=Nigeria. PAK=Pakistan. PHL=Philippines. RUS=Russian Federation. THA=Thailand. TUR=Turkey. USA=United States of America. VNM=Vietnam. ZAF=South Africa.

High variation in cross-country infection rates and IFR persisted, even after standardising for key factors such as seasonality, age, BMI, and other factors already described (figure 2). Between Jan 1, 2020, and Sept 30, 2021, countries such as Iceland and Singapore were largely successful in preventing infection and death (figure 2). On the contrary, countries such as India, Bolivia, and Peru had high standardised infection rates and high IFRs (figure 2). Taiwan and Vietnam did well in preventing infections yet had high IFRs (figure 2). Meanwhile, Georgia and Qatar had the opposite experience, with less success preventing infection, but low IFRs (figure 2).

**Figure 3:**
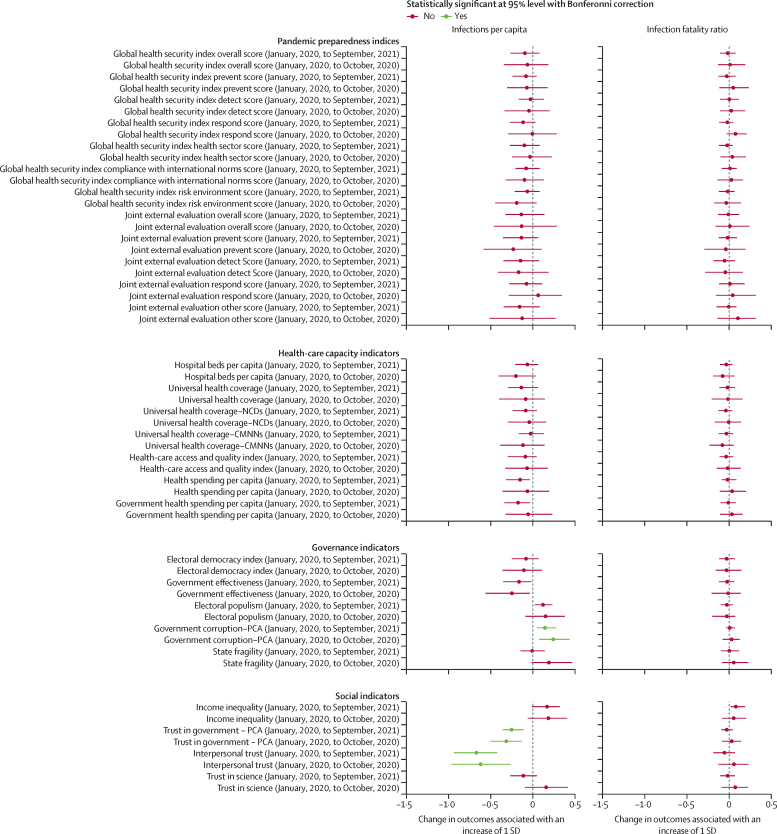
Associations between key preparedness, capacity, governance, and social indicators and infection rates and IFR The left column shows estimated associations between indicators of key contextual factors (pandemic preparedness indices, health-care capacity indicators, governance indicators, and social indicators) and infections per capita. The right column shows estimated associations between key contextual factors and the infection-fatality ratio. Red indicates the association is not significant and green indicates the association is significant at a 95% CI with a Bonferroni correction. CMNN=communicable, maternal, neonatal, and nutritional disease. IFR=infection-fatality ratio. NCD=non-communicable disease. PCA=principal component analysis.

Traditional pandemic preparedness indices, such as the JEE and GHS Index (and their components), were not associated with standardised infection rates or standardised IFRs (figure 3). Similarly, health-care capacity indicators were not favourably associated with standardised infection rates or IFRs (figure 3). More government corruption was associated with higher levels of standardised infections throughout the pandemic, and no other governance variables were associated with standardised IFR (figure 3). Finally, trust in government and interpersonal trust were associated with standardised infection rates, for both the first 10 months of the pandemic and the entire pandemic until Sept 30, 2021 (figure 3). Moreover, the magnitude of these associations was substantive and persistent across nearly all of the sensitivity analyses (appendix p 47). If our modelled associations are causal, an increase in trust of governments such that all countries had societies that attained at least the amount of trust in government or interpersonal trust measured in Denmark, which is in the 75th percentile across these spectrums, might have reduced global infections by 12·9% (95% UI 5·7–17·8) for government trust and 40·3% (24·3–51·4) for interpersonal trust. The appendix (p 14) reports the correlation across all of the health-care, governance, and social indicators assessed to provide information about how these factors might be related. Crucially, low interpersonal trust is highly correlated with income inequality and government corruption.

**Figure 4:**
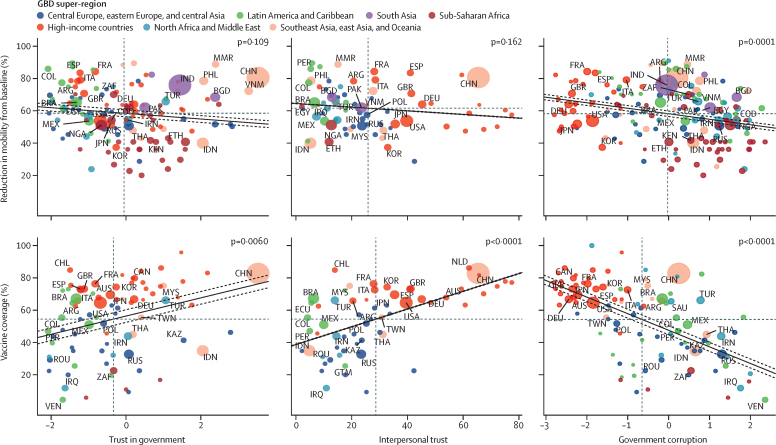
Association between trust and government corruption, and vaccine coverage and change in mobility The size of each circle represents total population. The solid line represents the fit of the linear regression for the two variables, and dotted lines represent the 95% CI. GBD=Global Burden of Diseases, Injuries, and Risk Factors Study.

To assess how government trust, interpersonal trust, and government corruption might have contributed to reductions in infection rates, we assessed the association between these factors and rates of COVID-19 vaccine coverage and reductions in mobility (figure 4). More interpersonal trust (p<0·0001), more government trust (p=0·0060), and less government corruption (p<0·0001) were associated with greater COVID-19 vaccine coverage (as of Sept 30, 2021). Less government corruption was also associated with greater reduction in mobility (p=0·0001).

## Discussion

We draw four main conclusions from this study. First, unlike mask mandates, physical-distancing guidelines, and other policy responses, many of the contextual factors that explain variation in SARS-CoV-2 infections and IFR are not factors that can be readily influenced by policy makers. For infections, those factors are environmental seasonality, altitude, and GDP per capita. Percentage of population living below 100 m and GDP per capita explained a large proportion of cross-country variation in infections. Infections rise during winter—the so-called flu season—and are, more precisely, associated with the relative risk of pneumonia. Similarly, occurrence of pneumonia and other lower respiratory infections is greater at higher altitudes. Explanations for this risk include climatic factors such as increased humidity, as well as seasonal behavioural modifications influencing spread, such as time spent indoors.^29^, ^30^, ^31^ For IFR, the dominant significant factors explaining cross-country variation are age structure, GDP per capita, and BMI. Age structure alone explains the largest proportion of cross-country variation in IFR globally. Much research on COVID-19 has shown that with each year of life lived, the relative risk of mortality with infection increases dramatically,^35^, ^36^ and this increase in risk goes beyond simply increased rates of other competing risks. Some research has shown that lower mortality for younger patients might be driven by molecular differences that allow younger patients to control the viral load, one predictor of mortality.^37^ Despite the importance of age, the effect of modifiable health risks in this analysis confirms previous research findings on the important role of high BMI,^38^ and suggests changes to smoking prevalence^39^ and ambient air pollution^40^ could influence outcomes during this pandemic. The prevalence of these risks related to non-communicable diseases is amenable to national and international health policy and represents an area of potential investment for mitigating the effects of future pandemics.^41^ Tobacco control policies, including increases in taxes and bans on advertising, have proven effective and cost-efficient in poor and wealthy nations alike.^42^, ^43^, ^44^ Strategies to promote lower BMI might include subsidising healthy food, such as fresh fruits, nuts, and vegetables, and taxing unhealthy foods such as sugar-sweetened beverages.^45^, ^46^, ^47^ Increasing reliance on non-polluting, renewable energy sources and ramping up air-quality monitoring have been central to progress in reducing ambient air pollution, even in difficult settings.^48^ In addition to risk-specific interventions, progress might also be advanced by working with WHO and other intergovernmental institutions on advancing international, national, and community-level strategies to counter the common commercial drivers behind tobacco use prevalence, obesity, and air pollution.^49^

The second major conclusion of this study is that important indicators of health-care capacity (the UHC effective coverage index and the HAQ Index) and of pandemic preparedness and response (JEE and GHS Index) were not correlated with cross-country variation in SARS-CoV-2 infections or IFR. The disconnect that exists between COVID-19 outcomes and the composite estimates of JEE and GHS Index scores also holds when the analysis is done on index components devoted to detection, prevention, and response, as well as the GHS Index health sector, commitment to international norms, and risk environment indices.

This analysis suggests that the JEE, GHS Index, and the UHC effective coverage index do not simply reflect capacities otherwise required for effective pandemic prevention and response, but fail to account for the consequences of poor leadership and dysfunctional political environments. A high ranking on the leading measures of health system capacity and pandemic preparedness has not only been insufficient for success in this pandemic, but also unnecessary. Various countries, including Burundi, the Philippines, and the Dominican Republic, are all examples of countries that rank low in GHS Index and JEE overall scores, and UHC effective coverage and HAQ Index scores, but so far have maintained low rates of standardised infections and IFR. We can further conclude that whatever proportion of cross-country variation in infections and IFR might be policy amenable, these existing measures of health-care and pandemic preparedness capacity offer no explanation.

The JEE, GHS Index, and measures of UHC are intended to be tools for identifying gaps in national capacity to direct financial and political support appropriately and were never intended to predict pandemic outcomes. JEEs were developed as a mechanism to identify gaps in a country's preparedness for developing National Action Plans and were not designed for cross-country comparability. Additionally, the aggregate measure is a weighting of the components and was not scored by the countries themselves. More than 100 nations have undertaken voluntary JEEs, and more than 60 countries developed National Action Plans for Health Security; the benefits of such exercises extend beyond preparing for the COVID-19 pandemic. Similarly, the 2019 GHS Index focuses on a national capacity and preparedness to prevent, limit, and respond to epidemic spread, with what the scores measures potentially having benefits associated with future pandemics. Measures assessed by these metrics, such as laboratory capacity, that have not yet been shown to drive outcomes in a pandemic, might well prove important against future emerging disease threats because such measures were not intended to predict outcomes for any one specific pandemic.

The third major conclusion of this study is that higher levels of trust (government and interpersonal) had large, statistically significant associations with fewer infections for the entire study period, but not with global variation in IFR. Less government corruption had a smaller but still statistically significant association with fewer infections and has no association with global variation in IFR. No other social factors (economic inequality or trust in science), state capacity measures (government effectiveness or state fragility), or features of political systems (electoral democracy or populism) had a statistically significant association with cross-country variation in infections or IFR. One way to quantify the contribution of trust to COVID-19 outcomes is with a counterfactual: if these associations are causal and all countries improved trust in government to the level of Denmark (approximately the 75th percentile of measured countries), this analysis suggests 12·9% fewer global infections would have occurred. Similarly, if all countries improved interpersonal trust to the same level (the 75th percentile of measured countries), the effect would be even larger—40·3% fewer global infections would have occurred.

These results support previous research that has found an association between trust and compliance with public health guidance.^50^, ^51^, ^52^, ^53^ When a virus emerges with high potential for spread, governments must be able to convince citizens to adopt essential public health measures. Doing so often requires behaviour change, from mask wearing and physical-distancing rules to following quarantine policies. This study accords with previous research that suggests that the success of that effort depends on two forms of trust: trust in governments^54^, ^55^ and interpersonal trust. Collectively, trust among people and in their government can change behaviour such that if people respond to directives and take protective health measures, they might expect other members of the community to do the same.^56^, ^57^ In much of the world, public health is a local, community-based endeavour and, in that context, the outsized role of interpersonal trust in this pandemic is unsurprising. Previous research has shown that public corruption contributes to lower trust in government and social institutions, which might reduce compliance with public health guidance and policies.^58^ Other smaller and preliminary studies have suggested links between trust in government, interpersonal trust, and public corruption and COVID-19 outcomes.^21^, ^59^, ^60^, ^61^, ^62^, ^63^

The fourth major conclusion of this study is that one specific pathway through which government and interpersonal trust and corruption affect COVID-19 outcomes might be through national vaccination rates. Lower levels of government corruption are associated with reduced mobility, which might signify compliance with physical-distancing guidance and stay-at-home orders. Further research and more complete global data on national adherence to non-pharmaceutical interventions might help identify the other specific pathways by which government and interpersonal trust have affected COVID-19 outcomes, particularly for the first 10 months of the pandemic before vaccines were widely available. Previous surveys in Liberia and the Democratic Republic of the Congo in Ebola outbreaks showed that trust in government was associated with compliance with government-recommended mitigation strategies, such as keeping physical distance and accepting vaccines.^54^, ^64^ Surveys from Italy, the Netherlands, and Switzerland during the 2009 H1N1 influenza pandemic found that government trust was associated with increased handwashing, physical distancing, vaccination, and other recommended behaviours.^50^, ^51^, ^52^

Fortunately, trust is something that can be fostered, even in a crisis. Governments and communities maintain or increase the public's trust by providing accurate, timely information about the pandemic, even when that information is still limited, and by clearly communicating the risk and relevant vulnerabilities.^65^ The identity of the messenger in risk communication can also improve or damage trust.^66^ The Ebola epidemic in west Africa was curtailed by rebuilding the public's trust in the government response. In Liberia, Ebola survivors were celebrated in communities, while community youth leaders, pastors, and imams were trained to conduct daily household surveillance and identify infected patients. In Sierra Leone, local community-liaison teams working in collaboration with WHO increased acceptance of the Ebola vaccine trials during and after the outbreak. In the COVID-19 pandemic, by contrast, some nations with historically low levels of government trust opted to promote politicians over public health experts for risk communication in the crisis, which might have contributed to reduced compliance with public health guidance and worsening health outcomes.^67^

Trust is a shared resource that enables networks of people to do collectively what individual actors cannot.^68^ It can be fostered in between crises through sustained investment. Previous research has assessed that trusting relations affect health outcomes through various forms of social capital, including bonding social capital (among networks of people who consider themselves to be similar), bridging social capital (among members of a network who perceive themselves as differing by age, racial or ethnic group, class, or other sociodemo-graphic characteristic), and linking social capital (across power or authority gradients such as the relationships between people and their law enforcement, health-care providers, medical researchers, or bankers).^68^, ^69^, ^70^, ^71^ Trusting relationships in all three of these forms of social capital have clearly affected health outcomes in the COVID-19 pandemic. Identifying which forms of social capital the role of trust has been the most important in this pandemic is beyond the scope of this paper, but the appendix (p 14) shows the correlations among the health-care, governance, and social indicators considered in this analysis and might provide some preliminary clues. For instance, low interpersonal trust is most correlated with income inequality and government corruption, suggesting those who are economically and socially disadvantaged and confront a society stacked against them might be naturally less inclined to trust others.^72^ The findings of future research should inform the longer-term potential application of the strategies used to promote resilience in disaster response and recovery by deepening trust within and between communities, from the economically and social disadvantaged and up to those in authority.^73^

This analysis has several limitations. First, the explanatory variables come from various sources that include population-based opinion surveys, a survey of expert opinions, reported government statistics, and modelled estimates. The findings for each explanatory variable should be assessed considering the quality of the data source. Second, although we attempted to propagate sources of uncertainty into the final results by running the analysis on each of 100 samples, an estimate of uncertainty was not available for most explanatory variables. Third, we prioritised explanatory value when deciding how to specify the functional forms of the models. Stage 1 and stage 2 variables are assumed to have log-log relationships with their respective outcomes, and the variables in stage 1, stage 2, and stage 3 are assumed to be independent. A fuller model could result in the use of interactions and non-linear effects, but we chose simple models by design and because of limitations in data. Fourth, we did not include country-specific response variables such as mask use, changes in mobility, testing, vaccination, and mandate imposition in our primary analysis of infections and IFR, given the challenges in identifying causal relationships using cross-sectional models. We included these variables in our supplementary analyses (appendix p 51) and in our stage 3 analysis but note that the temporality of these associations is not fully understood. Future research using time-series methods is needed to better understand how response measures influenced COVID-19 outcomes. Fifth, we adjusted for underlying influences on COVID-19 outcomes, including age structure and seasonality, but were unable to adjust for differentials in outcomes by sex given data limitations; future work is needed to better understand the degree of variability in outcomes due to differing sex population structures. Sixth, we include the most up-to-date information on variants and the most recent data but note that continually changing circumstances of the pandemic might influence these results. Seventh, although stage 1 was completed using subnational and national data, we focused our reporting and stage 2 and stage 3 analyses at the national level to focus on factors that could be influenced by national policy. Additional research is needed to better understand within-country variability, which is likely to be equally important in understanding differences in COVID-19 outcomes. Finally, this is an ecological analysis and was not designed to provide information about the causes of COVID-19 variation. Although we hope these results will spur discussion about the drivers of COVID-19 outcomes, a causal analysis would require more data and a different study design.

Uncertainty over the conditions that contribute to variation across countries in COVID-19 outcomes undermines efforts to convince global partners and policy makers to invest in preparing for future pandemics. Large, unexplained variation in differences in infections across countries speaks to the importance of further research in this area. In the meantime, this analysis identifies factors that explain some of the variation in the COVID-19 pandemic and suggests areas for potential investment in preparing for the next pandemic threat. Governments should invest in risk communication and community engagement strategies to boost the confidence that individuals have in government guidance in public health crises, especially in settings with historically low levels of government and interpersonal trust. Additionally, health promotion to address key modifiable risks might be an important condition for reducing fatalities in some pandemic scenarios.

For more on the **GHS Index** see https://www.ghsindex.org/

For the **code used in this analysis** see https://github.com/ihmeuw/covid-crosscountry-analysis

## Data sharing

Data used in this analysis are available to download from the Global Health Data Exchange website, at http://ghdx.healthdata.org/record/ihme-data/covid_19_pandemic_preparedness.
