# Supplementary material for: Pandemic preparedness and COVID-19: an exploratory analysis of infection and fatality rates, and contextual factors associated with preparedness in 177 countries, from Jan 1, 2020, to Sept 30, 2021
Source: Lancet. 2022 Apr 16;399(10334):1489–512. doi: 10.1016/S0140-6736(22)00172-6 (PMC8806194; doi:10.1016/S0140-6736(22)00172-6)
Supplement: Supplementary appendix [file mmc1.pdf]

THE LANCET

## Supplementary appendix

This appendix formed part of the original submission and has been peer reviewed. We post it as supplied by the authors.

Supplement to: COVID-19 National Preparedness Collaborators.Pandemic preparedness and COVID-19: an exploratory analysis of infection and fatality rates, and contextual factors associated with preparedness in 177 countries, from Jan 1, 2020, to Sept 30, 2021. *Lancet* 2022; published online Feb 1. https://doi.org/10.1016/S0140-6736(22)00172-6.

# Appendix 1: Supplemental methods and results for "**Pandemic preparedness and COVID-19: an exploratory analysis of infection and fatality rates and contextual factors associated with preparedness in 177 countries, from January 1, 2020, to September 30, 2021**"

This appendix provides further methodological and supplementary results for "**Pandemic preparedness and COVID-19: an exploratory analysis of infection and fatality rates and contextual factors associated with preparedness in 177 countries, from January 1, 2020, to September 30, 2021**."

# Table of Contents

***Section 1: Abbreviations*** ........ ***4***

***Section 2: Additional tables*** ........ ***5***

**2.1 GATHER** ........ **5**

**2.2 Covariate name, spatiotemporal coverage, data source, notes, assessment of missingness, and summary statistics** ........ **8**

**2.3 Analysis of multicollinearity between stage 1 variables** ........ **12**

**2.4 Correlation between stage 1 variables** ........ **13**

**2.5 Correlation between stage 2 variables** ........ **14**

**2.6 Results of stage 1 and stage 2 regressions** ........ **40**

**2.6.1 Results of stage 1 multivariate regressions, January 1, 2020 – September 30, 2021** ........ **40**

**2.6.2 Results of stage 2 bivariate regressions, January 1, 2020 – September 30, 2021** ........ **41**

**2.6.3 Results of stage 1 multivariate regressions, January 1, 2020 – October 15, 2020** ........ **42**

**2.6.4 Results of stage 2 bivariate regressions, January 1, 2020 – October 15, 2020** ........ **43**

***Section 3: Additional methods*** ........ ***44***

Figure 3.1.1: Modelling pathway for Stages 1-3 for IFR and infections ........ 44

**3.2 Proxy for previous exposure to beta coronaviruses** ........ **44**

Figure 3.2.1: Number of known and probable beta coronavirus bat species per grid cell ........ 45

**3.3 PCA analysis for trust in government and corruption** ........ **45**

***Section 4: Sensitivity analyses*** ........ ***47***

**4.1 Using a correction of 1% of median for log transformations** ........ **47**

Figure 4.1.1: Infections vs. IFR ........ 47

Figure 4.1.2: Bivariate results ........ 47

**4.2 Using centred and scaled variables** ........ **48**

Figure 4.2.1: Infections vs. IFR ........ 48

Figure 4.2.2 Bivariate results ........ 48

........ **48**

**4.3 Including variant spread in stage 1** ........ **49**

Figure 4.3.1: Infections vs. IFR ........ 49

Figure 4.3.2: Bivariate results ........ 49

***Section 5: Supplemental results*** ........ ***50***

**5.1 Additional bivariate analyses** ........ **50**

**5.2 Additional intermediate pathway analyses looking at testing and mask use** ........ **51**

**5.3 Additional analyses for the first phase of the pandemic (pre-variants and vaccines, January 1, 2020 – October 15, 2020)** ........ **52**

Figure 5.3.1: Intermediate mobility pathways ........ 52

**5.4 Analyses for seroprevalence study locations (n=303 nationals and subnationals, n=101 nationals only) .......... 53**
Figure 5.4.1: Seroprevalence study locations .......... 53
Figure 5.4.2: Infections vs. IFR .......... 54
Figure 5.4.3: Intermediate pathways, vaccine coverage, and mobility change, January 1, 2020 – September 30, 2021 .......... 54
Figure 5.4.4 Bivariate results .......... 55
**5.5 Analyses using only reported COVID deaths .......... 55**
Figure 5.5.1: Infections vs. IFR .......... 55
Figure 5.5.2 Bivariate plots .......... 56
**5.6 Scatter of raw data versus adjusted for stage 1 variables – IFR and cumulative infections .......... 56**
**5.7 Using 2021 Global Health Security Index metrics in lieu of 2019 metrics, bivariate results .......... 57**
***Section 6: Author Contributions .......... 57***
Managing the estimation or publications process .......... 57
Writing the first draft of the manuscript .......... 57
Primary responsibility for applying analytical methods to produce estimates .......... 57
Primary responsibility for seeking, cataloguing, extracting, or cleaning data; designing or coding figures and tables .......... 57
Providing data or critical feedback on data sources .......... 58
Developing methods or computational machinery .......... 58
Providing critical feedback on methods or results .......... 58
Drafting the work or revising is critically for important intellectual content .......... 58
Managing the overall research enterprise .......... 58
***Section 7: References .......... 58***

## Section 1: Abbreviations

BMI: body-mass index
COVID-19: coronavirus disease 2019
JEE: Joint External Evaluation
GBD: Global Burden of Diseases, Injuries, and Risk Factors Study
GHS Index: Global Health Security Index
HAQ Index: Healthcare Access and Quality Index
IFR: infection-fatality ratio
IHR: infection-hospitalisation ratio
IDR: infection-detection ratio
IHME: Institute for Health Metrics and Evaluation
PCA: principal component analysis
SARS-CoV2: severe acute respiratory syndrome coronavirus 2
WHO: World Health Organization
UHC effective coverage index: universal health coverage effective coverage index
UI: uncertainty interval

# Section 2: Additional tables

## 2.1 GATHER

Checklist of information that should be included in new reports of global health estimates

| Item # | Checklist item | Reported on page # |
| --- | --- | --- |
| **Objectives and funding** | | |
| 1 | Define the indicator(s), populations (including age, sex, and geographic entities), and time period(s) for which estimates were made. | Summary. Main Text: Introduction, Methods (Overview). |
| 2 | List the funding sources for the work. | Summary. Main Text: Acknowledgements and declarations. |
| **Data Inputs** | | |
| *For all data inputs from multiple sources that are synthesized as part of the study:* | | |
| 3 | Describe how the data were identified and how the data were accessed. | Main Text: Methods. Supplementary Appendix, Section 3. |
| 4 | Specify the inclusion and exclusion criteria. Identify all ad-hoc exclusions. | Main Text: Methods. |
| 5 | Provide information on all included data sources and their main characteristics. For each data source used, report reference information or contact name/institution, population represented, data collection method, year(s) of data collection, sex and age range, diagnostic criteria or measurement method, and sample size, as relevant. | Supplementary Appendix: Section 2.0, Table 2. Main characteristics of data, metadata, and/or NIDs available through: http://ghdx.healthdata.org/ (upon publication) |
| 6 | Identify and describe any categories of input data that have potentially important biases (e.g., based on characteristics listed in item 5). | Main text: Limitations |

| | | |
|---|---|---|
| | | section. Supplementary appendix (biases for input data in each modelling step identified in each section) |
| *For data inputs that contribute to the analysis but were not synthesized as part of the study:* | | |
| 7 | Describe and give sources for any other data inputs. | N/A |
| *For all data inputs:* | | |
| 8 | Provide all data inputs in a file format from which data can be efficiently extracted (e.g., a spreadsheet rather than a PDF), including all relevant meta-data listed in item 5. For any data inputs that cannot be shared because of ethical or legal reasons, such as third-party ownership, provide a contact name or the name of the institution that retains the right to the data. | Available through: http://ghdx.healthdata.org/ (upon publication) |
| Data analysis | | |
| 9 | Provide a conceptual overview of the data analysis method. A diagram may be helpful. | Main text: Methods |
| 10 | Provide a detailed description of all steps of the analysis, including mathematical formulae. This description should cover, as relevant, data cleaning, data pre-processing, data adjustments and weighting of data sources, and mathematical or statistical model(s). | Main text: Methods |
| 11 | Describe how candidate models were evaluated and how the final model(s) were selected. | Supplementary Appendix: Section 3 |
| 12 | Provide the results of an evaluation of model performance, if done, as well as the results of any relevant sensitivity analysis. | Supplementary Appendix: Sections 3.0 & 4.0 |
| 13 | Describe methods for calculating uncertainty of the estimates. State which sources of uncertainty were, and were not, accounted for in the uncertainty analysis. | Main Text: Methods; Supplementary Appendix |
| 14 | State how analytic or statistical source code used to generate estimates can be accessed. | Available through: http://ghdx.healthdata.org/ (upon publication) |
| Results and Discussion | | |
| 15 | Provide published estimates in a file format from which data can be efficiently extracted. | Available through: http://ghdx. |

| | | |
|---|---|---|
| | | healthdata.org/ (upon publication) |
| 16 | Report a quantitative measure of the uncertainty of the estimates (e.g. uncertainty intervals). | Available through: http://ghdx.healthdata.org/ (upon publication) |
| 17 | Interpret results in light of existing evidence. If updating a previous set of estimates, describe the reasons for changes in estimates. | Main Text: Discussion |
| 18 | Discuss limitations of the estimates. Include a discussion of any modelling assumptions or data limitations that affect interpretation of the estimates. | Main Text: Limitations |

## 2.2 Covariate name, spatiotemporal coverage, data source, notes, assessment of missingness, and summary statistics

| Short name | Covariate name | Temporal coverage | Spatial coverage | Data source | Notes | N (%) missing | Median (IQR) | Range |
|---|---|---|---|---|---|---|---|---|
| **Pneumonia RR** | Relative risk of death from pneumonia divided by the average risk of death from pneumonia | 2013-2019 | National and subnational | Modelling COVID-19 scenarios for the United States[1] | Varies weekly | 0 (0%) | 0.93 (0.91 – 0.95) | 0.76 – 1.22 |
| **Age** | Age structure of the population (5-year age bins) | 2020 | National and subnational | Global Burden of Disease Study 2019[2] | | 0 (0%) | - | - |
| **Altitude** | % of population living below 100 m | 2015 | National and subnational | Global Burden of Disease Study 2019[2] | | 0 (0%) | 30.4 (1.5 – 63.9) | 0.0 – 100.0 |
| **Population density** | % of population living above 1000 people/km$^2$ | 2020 | National and subnational | Modelling COVID-19 scenarios for the United States[1] | | 0 (0%) | 51.9 (36.2 – 69.6) | 0.0 – 100.0 |
| **Air pollution** | $PM_{2.5}$ air pollution concentration (mg/m$^3$) | 2019 | National and subnational | Global Burden of Disease Study 2019[2] | | 0 (0%) | 18.5 (10.2 – 34.2) | 3.5 - 217.5 |
| **Smoking prevalence** | Age-standardised tobacco smoking prevalence | 2019 | National and subnational | Global Burden of Disease Study 2019[2] | | 0 (0%) | 0.17 (0.12 – 0.22) | 0.04 -0.44 |
| **GDP** | Gross domestic product per capita | 2019 | National and subnational | Global Burden of Disease Study 2019[2] | | 0 (0%) | 15,722.8 (7345.9 – 39772.2) | 131.8 – 102,793.9 |
| **BMI** | Population-adjusted body-mass index | 2019 | National and subnational | Global Burden of Disease Study 2019[2] | | 0 (0%) | 26.5 (24.5 – 28.2) | 20.5 – 30.8 |
| **UHC** | Universal health coverage | 2019 | Nationals | Global Burden of Disease Study 2019, Measuring Universal Health Coverage[2,3] | Included 2 subcomponents – communicable and non-communicable, maternal, and neonatal | 0 (0%) | 61.2 (49.6 – 72.7) | 22.3 – 96.3 |
| **Mobility** | Largest % change from a January 2020 baseline | 2020-2021 | National and subnational | Modelling COVID-19 scenarios for the United States[1] | | 0 (0%) | 61.3 (51.2 – 72.9) | 20.0 – 93.2 |
| **Mandates** | Mean fraction of mandates on over period | 2020-2021 | National and subnational | Modelling COVID-19 scenarios for the United States[1] | Locations measured at the subnational level had a mandate in place if 2/3 of locations or more had it in place on a given date | 1/361 (0.3%) | 44.0 (33.3 – 49.5) | 0.0 – 85.5 |

| | | | | | | | | |
|---|---|---|---|---|---|---|---|---|
| **Testing** | Mean testing per capita over the time period | 2020-2021 | National and subnational | Modeling COVID-19 scenarios for the United States[1] | | 0 (0%) | 71.4 / 100,000 (21.5 / 100,000 – 200.7 / 100,000) | 0.3 / 100,000 – 1713.3 / 100,000 |
| **Vaccine coverage** | Maximum vaccine coverage over the time period | 2021 | National and subnational | Modeling COVID-19 scenarios for the United States[1] | | 0 (0%) | 50.9 (27.4 – 65.0) | 0.0 – 94.3 |
| **COPD** | Age-standardised COPD prevalence | 2019 | National and subnational | Global Burden of Disease Study 2019[2] | | 0 (0%) | 2.3 (2.0 - 3.2) | 0.7-4.4 |
| **Cancer** | Age-standardised cancer prevalence | 2019 | National and subnational | Global Burden of Disease Study 2019[2] | | 0 (0%) | 5.6 (4.3-8.1) | 2.1 – 20.8 |
| **Interpersonal trust** | Trust in other people | 2017-2021 | National only | WORLD VALUES SURVEY WAVE 7[4] | Trust coded as those who answered "most people can be trusted" on Q57 | 102/177 (57.6%) | 20.7 (11.9 – 32.9) | 2.1 – 77.4 |
| **Trust in science** | Trust in science | 2018 | National only | Wellcome Global Monitor Survey[5] | Those that answered "A lot" to trusting science | 40/177 (22.6%) | 29.8 (23.0 – 39.4) | 6.7 – 76.0 |
| **Trust in government - WVS** | Trust in one's government | 2017-2021 | National only | WORLD VALUES SURVEY WAVE 7[4] | Coded as "A great deal" or "Quite a lot" on Q71 asking about confidence in government | 102/177 (57.6%) | 40.1 (37.3 – 51.7) | 0.0 – 95.2 |
| **Trust in government - Gallup** | Trust in one's government | | National only | Gallup World Poll[6] | | 46/177 (26%) | 53.7 (39.1 – 68.9) | 15.7 – 99.3 |
| **Governmental Corruption - CPI** | Perceived governmental corruption | 2020 | National only | Transparency International[7] | | 10/177 (5.6%) | 40.0 (29.0 – 56.5) | 12.0 – 88.0 |
| **JEE** | Joint External Evaluation Components and Prevent Epidemics' Preparedness overall score | 2016-2021 | National only | WHO and Prevent Epidemics[8] | Only places that have completed a JEE; overall score is a summary variable of JEE components created by Prevent Epidemics | 89/177 (50.3%) | Overall score: 50.0 (38.0 -69.3) | Overall score: 26.0 – 93.0 |

| | | | | | | | | |
|---|---|---|---|---|---|---|---|---|
| **GHSI** | Global Health Security Index components and overall score | 2019 | National only | Global Health Security Index 2019[9] | Weighted average of the other components | 7/177 (4.0%) | Overall score: 39.3 (31.9-51.6) | Overall score: 16.2 – 83.5 |
| **GINI** | Gini Index | | National only | SWIID v8.2[10] & Bosancinau[11] | | 27/177 (15.3%) | 38.38 (33.10 – 44.05) | 23.68 – 65.11 |
| **HAQ Index** | Healthcare Access and Quality Index | 2019 | National only | Global Burden of Disease Study 2019[2] | | 0 (0%) | 63.3 (42.4 -79.5) | 12.8 – 96.8 |
| **Electoral populism** | Populism-based campaign run | | National only | Populism in Power[12] & Bosancinau[11] | Whether a democratically elected head of government ran a populist campaign | 0 (0%) | 0 (0 – 0) | 0 -1 |
| **Government effectiveness** | | | National only | World Bank Indicators[13] & Bosancinau[11] | Perceived quality of public services, its provision and providers | 1/177 (0.6%) | -0.09 (-0.66 – 0.57) | -2.45 – 2.23 |
| **State fragility** | State Fragility Index | | National only | State Fragility Index[14] & Bosancinau[11] | Incapacity to provide essential public goods and services and cope with shocks | 19/177 (10.7%) | 7.0 (3.0 – 12.0) | 0 .0 – 24.0 |
| **Index of federalism** | | 2017 | National only | Database of Political Institutions[15] | Extent to which power and decision-making processes are decentralised | 54/177 (30.5%) | -0.07 (-0.59 – 0.68) | -1.03 – 1.51 |
| **Bureaucracy corruption** | Public sector corruption | | National only | Varieties of Democracy Institute, Version 10[16,17] | Pervasiveness of bureaucratic corruption in the public sector | 13/177 (7.3%) | 0.48 (0.19 – 0.72) | 0 – 0.97 |
| **EDI** | Electoral Democracy Index | 2020 | National only | Varieties of Democracy Institute, Version 11[18,19] | Aggregate indicator combining free and fair elections, free association, freedom of expression, and access to alternative information, suffrage, and elected officials | 13/177 (7.3%) | 0.32 (0.05 – 0.63) | 0.00 – 0.86 |

| | | | | | | | | |
|---|---|---|---|---|---|---|---|---|
| **Beds per capita** | Number of hospital beds per capita before start of the pandemic | 2019 | National and subnational | Global Burden of Disease Study 2019[2] | | 0 (0%) | 206.0 / 100,000 (102.7 / 100,000 – 328.3 / 100,000) | 18.4 / 100,000 – 1185.9 / 100,000 |
| **Health spending per capita** | Total health expenditure per capita | 2020 | National only | Global Burden of Disease Health Financing Collaborator Network[20] | Mean value | 0 (0%) | 538.1 (87.1 – 1744.7) | 5.0 – 6869.6 |
| **Government health spending per capita** | Government health spending per capita | 2020 | National only | Global Burden of Disease Health Financing Collaborator Network[20] | Mean value | 0 (0%) | 932.7 (252.2-2518.3) | 23.5 – 12131.9 |
| **Bats** | Average number of beta-coronavirus host bat species in a given location | 2021 Bats & Ranges | National only | IUCN[21] & Verena Consortium[22] | See section 3.1 below | 0 (0%) | 11.5 (4.2 - 16.6) | 0.0 – 42.0 |

## 2.3 Analysis of multicollinearity between stage 1 variables

| Covariates | Variance inflation factors | |
|---|---|---|
| | Cumulative infections per capita | IFR |
| GDP per capita | 1.280 | 2.860 |
| % of population living above 1000 people/km$^2$ | 1.242 | 1.181 |
| Average number of beta-coronavirus host bat species | 1.105 | 1.299 |
| % of population living below 100 m | 1.245 | - |
| BMI | - | 2.432 |
| Smoking prevalence | - | 1.486 |
| $PM_{2.5}$ air pollution concentration (mg/m$^3$) | - | 1.455 |
| COPD prevalence | - | 1.703 |
| Cancer prevalence | - | 1.215 |

## 2.4 Correlation between stage 1 variables

| | GDP per capita | % of population living above 1000 people/km$^2$ | % of population living below 100 m | Average number of beta-coronavirus host bat species | BMI | $PM_{2.5}$ air pollution concentration (mg/m$^3$) | Smoking prevalence | COPD prevalence | Cancer prevalence |
|---|---|---|---|---|---|---|---|---|---|
| GDP per capita | **1** | 0.343 | 0.135 | -0.380 | 0.709 | -0.594 | 0.417 | 0.336 | 0.449 |
| % of population living above 1000 people/km$^2$ | 0.343 | **1** | 0.231 | -0.122 | 0.287 | -0.205 | 0.054 | 0.095 | 0.224 |
| % of population living below 100 m | 0.135 | 0.231 | **1** | -0.063 | - | - | - | - | - |
| Average number of beta-coronavirus host bat species | -0.380 | -0.122 | -0.063 | **1** | -0.526 | 0.345 | -0.077 | -0.102 | -0.087 |
| BMI | 0.709 | 0.287 | - | -0.526 | **1** | -0.584 | 0.193 | 0.117 | 0.446 |
| $PM_{2.5}$ air pollution concentration (mg/m$^3$) | -0.594 | -0.205 | - | 0.345 | -0.584 | **1** | -0.198 | -0.304 | -0.268 |
| Smoking prevalence | 0.417 | 0.054 | - | -0.077 | 0.193 | -0.198 | **1** | 0.224 | 0.420 |
| COPD prevalence | 0.336 | 0.095 | - | -0.102 | 0.117 | -0.304 | 0.224 | **1** | -0.166 |
| Cancer prevalence | 0.449 | 0.224 | - | -0.087 | 0.446 | -0.268 | 0.420 | -0.166 | **1** |

## 2.5 Correlation between stage 2 variables

| First variable | Correlated variable | Correlation | P-value |
|---|---|---|---|
| Electoral democracy index | Electoral populism | 0.004 | 0.9598 |
| Electoral democracy index | Global health security index compliance with international norms score | 0.404 | <0.0001 |
| Electoral democracy index | Global health security index detect score | 0.481 | <0.0001 |
| Electoral democracy index | Global health security index health sector score | 0.464 | <0.0001 |
| Electoral democracy index | Global health security index overall score | 0.558 | <0.0001 |
| Electoral democracy index | Global health security index prevent score | 0.476 | <0.0001 |
| Electoral democracy index | Global health security index respond score | 0.417 | <0.0001 |
| Electoral democracy index | Global health security index risk environment score | 0.635 | <0.0001 |
| Electoral democracy index | Government corruption - PCA | -0.721 | <0.0001 |
| Electoral democracy index | Government effectiveness | 0.631 | <0.0001 |
| Electoral democracy index | Government health spending per capita | 0.566 | <0.0001 |
| Electoral democracy index | Health spending per capita | 0.568 | <0.0001 |
| Electoral democracy index | Healthcare Access and Quality Index | 0.512 | <0.0001 |
| Electoral democracy index | Hospital beds per capita | 0.382 | <0.0001 |
| Electoral democracy index | Income inequality | -0.342 | <0.0001 |
| Electoral democracy index | Interpersonal trust | 0.416 | 0.0002 |
| Electoral democracy index | Joint external evaluation detect score | 0.359 | 0.0006 |
| Electoral democracy index | Joint external evaluation other score | 0.359 | 0.0006 |
| Electoral democracy index | Joint external evaluation overall score | 0.335 | 0.0014 |
| Electoral democracy index | Joint external evaluation prevent score | 0.343 | 0.0011 |
| Electoral democracy index | Joint external evaluation respond score | 0.259 | 0.0149 |
| Electoral democracy index | State fragility | -0.630 | <0.0001 |
| Electoral democracy index | Trust in government - PCA | -0.317 | 0.0002 |
| Electoral democracy index | Trust in science | 0.273 | 0.0013 |
| Electoral democracy index | Universal health coverage | 0.544 | <0.0001 |
| Electoral democracy index | Universal health coverage - CMNNs | 0.466 | <0.0001 |

| | | | |
|---|---|---|---|
| Electoral democracy index | Universal health coverage - NCDs | 0.516 | <0.0001 |
| Electoral populism | Electoral democracy index | 0.004 | 0.9598 |
| Electoral populism | Global health security index compliance with international norms score | 0.105 | 0.1738 |
| Electoral populism | Global health security index detect score | 0.136 | 0.0774 |
| Electoral populism | Global health security index health sector score | 0.261 | 0.0006 |
| Electoral populism | Global health security index overall score | 0.178 | 0.0201 |
| Electoral populism | Global health security index prevent score | 0.158 | 0.0395 |
| Electoral populism | Global health security index respond score | 0.136 | 0.0763 |
| Electoral populism | Global health security index risk environment score | 0.092 | 0.2309 |
| Electoral populism | Government corruption - PCA | -0.015 | 0.8482 |
| Electoral populism | Government effectiveness | 0.055 | 0.4657 |
| Electoral populism | Government health spending per capita | 0.063 | 0.4033 |
| Electoral populism | Health spending per capita | 0.083 | 0.2712 |
| Electoral populism | Healthcare Access and Quality Index | 0.164 | 0.0288 |
| Electoral populism | Hospital beds per capita | 0.155 | 0.0392 |
| Electoral populism | Income inequality | -0.066 | 0.4256 |
| Electoral populism | Interpersonal trust | -0.133 | 0.2536 |
| Electoral populism | Joint external evaluation detect score | 0.109 | 0.3121 |
| Electoral populism | Joint external evaluation other score | 0.092 | 0.3930 |
| Electoral populism | Joint external evaluation overall score | 0.094 | 0.3861 |
| Electoral populism | Joint external evaluation prevent score | 0.070 | 0.5189 |
| Electoral populism | Joint external evaluation respond score | 0.098 | 0.3653 |
| Electoral populism | State fragility | -0.116 | 0.1464 |
| Electoral populism | Trust in government - PCA | -0.082 | 0.3447 |
| Electoral populism | Trust in science | -0.026 | 0.7610 |
| Electoral populism | Universal health coverage | 0.114 | 0.1320 |
| Electoral populism | Universal health coverage - CMNNs | 0.181 | 0.0157 |
| Electoral populism | Universal health coverage - NCDs | 0.147 | 0.0506 |
| Global health security index compliance with international norms score | Electoral democracy index | 0.404 | <0.0001 |

| | | | |
|---|---|---|---|
| Global health security index compliance with international norms score | Electoral populism | 0.105 | 0.1738 |
| Global health security index compliance with international norms score | Global health security index detect score | 0.598 | <0.0001 |
| Global health security index compliance with international norms score | Global health security index health sector score | 0.563 | <0.0001 |
| Global health security index compliance with international norms score | Global health security index overall score | 0.714 | <0.0001 |
| Global health security index compliance with international norms score | Global health security index prevent score | 0.600 | <0.0001 |
| Global health security index compliance with international norms score | Global health security index respond score | 0.579 | <0.0001 |
| Global health security index compliance with international norms score | Global health security index risk environment score | 0.363 | <0.0001 |
| Global health security index compliance with international norms score | Government corruption - PCA | -0.396 | <0.0001 |
| Global health security index compliance with international norms score | Government effectiveness | 0.432 | <0.0001 |
| Global health security index compliance with international norms score | Government health spending per capita | 0.429 | <0.0001 |
| Global health security index compliance with international norms score | Health spending per capita | 0.442 | <0.0001 |
| Global health security index compliance with international norms score | Healthcare Access and Quality Index | 0.316 | <0.0001 |
| Global health security index compliance with international norms score | Hospital beds per capita | 0.249 | 0.0011 |
| Global health security index compliance with international norms score | Income inequality | -0.297 | 0.0003 |
| Global health security index compliance with international norms score | Interpersonal trust | 0.312 | 0.0073 |
| Global health security index compliance with international norms score | Joint external evaluation detect score | 0.517 | <0.0001 |

| | | | |
|---|---|---|---|
| Global health security index compliance with international norms score | Joint external evaluation other score | 0.447 | <0.0001 |
| Global health security index compliance with international norms score | Joint external evaluation overall score | 0.486 | <0.0001 |
| Global health security index compliance with international norms score | Joint external evaluation prevent score | 0.495 | <0.0001 |
| Global health security index compliance with international norms score | Joint external evaluation respond score | 0.416 | 0.0001 |
| Global health security index compliance with international norms score | State fragility | -0.345 | <0.0001 |
| Global health security index compliance with international norms score | Trust in government - PCA | 0.043 | 0.6209 |
| Global health security index compliance with international norms score | Trust in science | 0.245 | 0.0041 |
| Global health security index compliance with international norms score | Universal health coverage | 0.342 | <0.0001 |
| Global health security index compliance with international norms score | Universal health coverage - CMNNs | 0.272 | 0.0003 |
| Global health security index compliance with international norms score | Universal health coverage - NCDs | 0.319 | <0.0001 |
| Global health security index detect score | Electoral democracy index | 0.481 | <0.0001 |
| Global health security index detect score | Electoral populism | 0.136 | 0.0774 |
| Global health security index detect score | Global health security index compliance with international norms score | 0.598 | <0.0001 |
| Global health security index detect score | Global health security index health sector score | 0.727 | <0.0001 |
| Global health security index detect score | Global health security index overall score | 0.883 | <0.0001 |
| Global health security index detect score | Global health security index prevent score | 0.747 | <0.0001 |
| Global health security index detect score | Global health security index respond score | 0.676 | <0.0001 |
| Global health security index detect score | Global health security index risk environment score | 0.499 | <0.0001 |
| Global health security index detect score | Government corruption - PCA | -0.471 | <0.0001 |
| Global health security index detect score | Government effectiveness | 0.560 | <0.0001 |
| Global health security index detect score | Government health spending per capita | 0.522 | <0.0001 |

| | | | |
|---|---|---|---|
| Global health security index detect score | Health spending per capita | 0.541 | <0.0001 |
| Global health security index detect score | Healthcare Access and Quality Index | 0.510 | <0.0001 |
| Global health security index detect score | Hospital beds per capita | 0.326 | <0.0001 |
| Global health security index detect score | Income inequality | -0.276 | 0.0007 |
| Global health security index detect score | Interpersonal trust | 0.279 | 0.0169 |
| Global health security index detect score | Joint external evaluation detect score | 0.702 | <0.0001 |
| Global health security index detect score | Joint external evaluation other score | 0.663 | <0.0001 |
| Global health security index detect score | Joint external evaluation overall score | 0.670 | <0.0001 |
| Global health security index detect score | Joint external evaluation prevent score | 0.683 | <0.0001 |
| Global health security index detect score | Joint external evaluation respond score | 0.555 | <0.0001 |
| Global health security index detect score | State fragility | -0.537 | <0.0001 |
| Global health security index detect score | Trust in government - PCA | -0.123 | 0.1576 |
| Global health security index detect score | Trust in science | 0.157 | 0.0697 |
| Global health security index detect score | Universal health coverage | 0.526 | <0.0001 |
| Global health security index detect score | Universal health coverage - CMNNs | 0.460 | <0.0001 |
| Global health security index detect score | Universal health coverage - NCDs | 0.510 | <0.0001 |
| Global health security index health sector score | Electoral democracy index | 0.464 | <0.0001 |
| Global health security index health sector score | Electoral populism | 0.261 | 0.0006 |
| Global health security index health sector score | Global health security index compliance with international norms score | 0.563 | <0.0001 |
| Global health security index health sector score | Global health security index detect score | 0.727 | <0.0001 |
| Global health security index health sector score | Global health security index overall score | 0.913 | <0.0001 |
| Global health security index health sector score | Global health security index prevent score | 0.847 | <0.0001 |
| Global health security index health sector score | Global health security index respond score | 0.780 | <0.0001 |
| Global health security index health sector score | Global health security index risk environment score | 0.673 | <0.0001 |
| Global health security index health sector score | Government corruption - PCA | -0.603 | <0.0001 |
| Global health security index health sector score | Government effectiveness | 0.695 | <0.0001 |
| Global health security index health sector score | Government health spending per capita | 0.697 | <0.0001 |
| Global health security index health sector score | Health spending per capita | 0.712 | <0.0001 |
| Global health security index health sector score | Healthcare Access and Quality Index | 0.721 | <0.0001 |
| Global health security index health sector score | Hospital beds per capita | 0.445 | <0.0001 |

| | | | |
|---|---|---|---|
| Global health security index health sector score | Income inequality | -0.463 | <0.0001 |
| Global health security index health sector score | Interpersonal trust | 0.561 | <0.0001 |
| Global health security index health sector score | Joint external evaluation detect Score | 0.796 | <0.0001 |
| Global health security index health sector score | Joint external evaluation other score | 0.796 | <0.0001 |
| Global health security index health sector score | Joint external evaluation overall score | 0.811 | <0.0001 |
| Global health security index health sector score | Joint external evaluation prevent score | 0.790 | <0.0001 |
| Global health security index health sector score | Joint external evaluation respond score | 0.749 | <0.0001 |
| Global health security index health sector score | State fragility | -0.653 | <0.0001 |
| Global health security index health sector score | Trust in government - PCA | -0.086 | 0.3206 |
| Global health security index health sector score | Trust in science | 0.343 | <0.0001 |
| Global health security index health sector score | Universal health coverage | 0.731 | <0.0001 |
| Global health security index health sector score | Universal health coverage - CMNNs | 0.668 | <0.0001 |
| Global health security index health sector score | Universal health coverage - NCDs | 0.722 | <0.0001 |
| Global health security index overall score | Electoral democracy index | 0.558 | <0.0001 |
| Global health security index overall score | Electoral populism | 0.178 | 0.0201 |
| Global health security index overall score | Global health security index compliance with international norms score | 0.714 | <0.0001 |
| Global health security index overall score | Global health security index detect score | 0.883 | <0.0001 |
| Global health security index overall score | Global health security index health sector score | 0.913 | <0.0001 |
| Global health security index overall score | Global health security index prevent score | 0.913 | <0.0001 |
| Global health security index overall score | Global health security index respond score | 0.861 | <0.0001 |
| Global health security index overall score | Global health security index risk environment score | 0.718 | <0.0001 |
| Global health security index overall score | Government corruption - PCA | -0.666 | <0.0001 |
| Global health security index overall score | Government effectiveness | 0.758 | <0.0001 |
| Global health security index overall score | Government health spending per capita | 0.705 | <0.0001 |
| Global health security index overall score | Health spending per capita | 0.728 | <0.0001 |
| Global health security index overall score | Healthcare Access and Quality Index | 0.705 | <0.0001 |
| Global health security index overall score | Hospital beds per capita | 0.431 | <0.0001 |
| Global health security index overall score | Income inequality | -0.424 | <0.0001 |
| Global health security index overall score | Interpersonal trust | 0.547 | <0.0001 |
| Global health security index overall score | Joint external evaluation detect score | 0.849 | <0.0001 |

| | | | |
|---|---|---|---|
| Global health security index overall score | Joint external evaluation other score | 0.815 | <0.0001 |
| Global health security index overall score | Joint external evaluation overall score | 0.844 | <0.0001 |
| Global health security index overall score | Joint external evaluation prevent score | 0.842 | <0.0001 |
| Global health security index overall score | Joint external evaluation respond score | 0.751 | <0.0001 |
| Global health security index overall score | State fragility | -0.691 | <0.0001 |
| Global health security index overall score | Trust in government - PCA | -0.065 | 0.4568 |
| Global health security index overall score | Trust in science | 0.337 | 0.0001 |
| Global health security index overall score | Universal health coverage | 0.723 | <0.0001 |
| Global health security index overall score | Universal health coverage - CMNNs | 0.654 | <0.0001 |
| Global health security index overall score | Universal health coverage - NCDs | 0.703 | <0.0001 |
| Global health security index prevent score | Electoral democracy index | 0.476 | <0.0001 |
| Global health security index prevent score | Electoral populism | 0.158 | 0.0395 |
| Global health security index prevent score | Global health security index compliance with international norms score | 0.600 | <0.0001 |
| Global health security index prevent score | Global health security index detect score | 0.747 | <0.0001 |
| Global health security index prevent score | Global health security index health sector score | 0.847 | <0.0001 |
| Global health security index prevent score | Global health security index overall score | 0.913 | <0.0001 |
| Global health security index prevent score | Global health security index respond score | 0.739 | <0.0001 |
| Global health security index prevent score | Global health security index risk environment score | 0.667 | <0.0001 |
| Global health security index prevent score | Government corruption - PCA | -0.609 | <0.0001 |
| Global health security index prevent score | Government effectiveness | 0.700 | <0.0001 |
| Global health security index prevent score | Government health spending per capita | 0.680 | <0.0001 |
| Global health security index prevent score | Health spending per capita | 0.691 | <0.0001 |
| Global health security index prevent score | Healthcare Access and Quality Index | 0.689 | <0.0001 |
| Global health security index prevent score | Hospital beds per capita | 0.418 | <0.0001 |
| Global health security index prevent score | Income inequality | -0.438 | <0.0001 |
| Global health security index prevent score | Interpersonal trust | 0.509 | <0.0001 |
| Global health security index prevent score | Joint external evaluation detect score | 0.813 | <0.0001 |
| Global health security index prevent score | Joint external evaluation other score | 0.775 | <0.0001 |
| Global health security index prevent score | Joint external evaluation overall score | 0.818 | <0.0001 |
| Global health security index prevent score | Joint external evaluation prevent score | 0.824 | <0.0001 |

| | | | |
|---|---|---|---|
| Global health security index prevent score | Joint external evaluation respond score | 0.731 | <0.0001 |
| Global health security index prevent score | State fragility | -0.636 | <0.0001 |
| Global health security index prevent score | Trust in government - PCA | -0.062 | 0.4765 |
| Global health security index prevent score | Trust in science | 0.359 | <0.0001 |
| Global health security index prevent score | Universal health coverage | 0.699 | <0.0001 |
| Global health security index prevent score | Universal health coverage - CMNNs | 0.654 | <0.0001 |
| Global health security index prevent score | Universal health coverage - NCDs | 0.679 | <0.0001 |
| Global health security index respond score | Electoral democracy index | 0.417 | <0.0001 |
| Global health security index respond score | Electoral populism | 0.136 | 0.0763 |
| Global health security index respond score | Global health security index compliance with international norms score | 0.579 | <0.0001 |
| Global health security index respond score | Global health security index detect score | 0.676 | <0.0001 |
| Global health security index respond score | Global health security index health sector score | 0.780 | <0.0001 |
| Global health security index respond score | Global health security index overall score | 0.861 | <0.0001 |
| Global health security index respond score | Global health security index prevent score | 0.739 | <0.0001 |
| Global health security index respond score | Global health security index risk environment score | 0.543 | <0.0001 |
| Global health security index respond score | Government corruption - PCA | -0.536 | <0.0001 |
| Global health security index respond score | Government effectiveness | 0.592 | <0.0001 |
| Global health security index respond score | Government health spending per capita | 0.524 | <0.0001 |
| Global health security index respond score | Health spending per capita | 0.570 | <0.0001 |
| Global health security index respond score | Healthcare Access and Quality Index | 0.523 | <0.0001 |
| Global health security index respond score | Hospital beds per capita | 0.253 | 0.0009 |
| Global health security index respond score | Income inequality | -0.282 | 0.0005 |
| Global health security index respond score | Interpersonal trust | 0.473 | <0.0001 |
| Global health security index respond score | Joint external evaluation detect score | 0.722 | <0.0001 |
| Global health security index respond score | Joint external evaluation other score | 0.699 | <0.0001 |
| Global health security index respond score | Joint external evaluation overall score | 0.737 | <0.0001 |
| Global health security index respond score | Joint external evaluation prevent score | 0.717 | <0.0001 |
| Global health security index respond score | Joint external evaluation respond score | 0.697 | <0.0001 |
| Global health security index respond score | State fragility | -0.526 | <0.0001 |
| Global health security index respond score | Trust in government - PCA | 0.025 | 0.7721 |

| | | | |
|---|---|---|---|
| Global health security index respond score | Trust in science | 0.259 | 0.0024 |
| Global health security index respond score | Universal health coverage | 0.559 | <0.0001 |
| Global health security index respond score | Universal health coverage - CMNNs | 0.469 | <0.0001 |
| Global health security index respond score | Universal health coverage - NCDs | 0.531 | <0.0001 |
| Global health security index risk environment score | Electoral democracy index | 0.635 | <0.0001 |
| Global health security index risk environment score | Electoral populism | 0.092 | 0.2309 |
| Global health security index risk environment score | Global health security index compliance with international norms score | 0.363 | <0.0001 |
| Global health security index risk environment score | Global health security index detect score | 0.499 | <0.0001 |
| Global health security index risk environment score | Global health security index health sector score | 0.673 | <0.0001 |
| Global health security index risk environment score | Global health security index overall score | 0.718 | <0.0001 |
| Global health security index risk environment score | Global health security index prevent score | 0.667 | <0.0001 |
| Global health security index risk environment score | Global health security index respond score | 0.543 | <0.0001 |
| Global health security index risk environment score | Government corruption - PCA | -0.870 | <0.0001 |
| Global health security index risk environment score | Government effectiveness | 0.940 | <0.0001 |
| Global health security index risk environment score | Government health spending per capita | 0.782 | <0.0001 |
| Global health security index risk environment score | Health spending per capita | 0.786 | <0.0001 |
| Global health security index risk environment score | Healthcare Access and Quality Index | 0.864 | <0.0001 |
| Global health security index risk environment score | Hospital beds per capita | 0.542 | <0.0001 |
| Global health security index risk environment score | Income inequality | -0.449 | <0.0001 |
| Global health security index risk environment score | Interpersonal trust | 0.702 | <0.0001 |
| Global health security index risk environment score | Joint external evaluation detect score | 0.842 | <0.0001 |
| Global health security index risk environment score | Joint external evaluation other score | 0.830 | <0.0001 |
| Global health security index risk environment score | Joint external evaluation overall score | 0.851 | <0.0001 |
| Global health security index risk environment score | Joint external evaluation prevent score | 0.852 | <0.0001 |
| Global health security index risk environment score | Joint external evaluation respond score | 0.761 | <0.0001 |
| Global health security index risk environment score | State fragility | -0.892 | <0.0001 |
| Global health security index risk environment score | Trust in government - PCA | -0.068 | 0.4327 |
| Global health security index risk environment score | Trust in science | 0.430 | <0.0001 |
| Global health security index risk environment score | Universal health coverage | 0.858 | <0.0001 |
| Global health security index risk environment score | Universal health coverage - CMNNs | 0.852 | <0.0001 |

| | | | |
|---|---|---|---|
| Global health security index risk environment score | Universal health coverage - NCDs | 0.849 | <0.0001 |
| Government corruption - PCA | Electoral democracy index | -0.721 | <0.0001 |
| Government corruption - PCA | Electoral populism | -0.015 | 0.8482 |
| Government corruption - PCA | Global health security index compliance with international norms score | -0.396 | <0.0001 |
| Government corruption - PCA | Global health security index detect score | -0.471 | <0.0001 |
| Government corruption - PCA | Global health security index health sector score | -0.603 | <0.0001 |
| Government corruption - PCA | Global health security index overall score | -0.666 | <0.0001 |
| Government corruption - PCA | Global health security index prevent score | -0.609 | <0.0001 |
| Government corruption - PCA | Global health security index respond score | -0.536 | <0.0001 |
| Government corruption - PCA | Global health security index risk environment score | -0.87 | <0.0001 |
| Government corruption - PCA | Government corruption - PCA | 1 | <0.0001 |
| Government corruption - PCA | Government effectiveness | -0.911 | <0.0001 |
| Government corruption - PCA | Government health spending per capita | -0.774 | <0.0001 |
| Government corruption - PCA | Health spending per capita | -0.773 | <0.0001 |
| Government corruption - PCA | Healthcare Access and Quality Index | -0.714 | <0.0001 |
| Government corruption - PCA | Hospital beds per capita | -0.464 | <0.0001 |
| Government corruption - PCA | Income inequality | 0.424 | <0.0001 |
| Government corruption - PCA | Interpersonal trust | -0.727 | <0.0001 |
| Government corruption - PCA | Joint external evaluation detect score | -0.767 | <0.0001 |
| Government corruption - PCA | Joint external evaluation other score | -0.69 | <0.0001 |
| Government corruption - PCA | Joint external evaluation overall score | -0.749 | <0.0001 |
| Government corruption - PCA | Joint external evaluation prevent score | -0.755 | <0.0001 |
| Government corruption - PCA | Joint external evaluation respond score | -0.672 | <0.0001 |
| Government corruption - PCA | State fragility | 0.747 | <0.0001 |
| Government corruption - PCA | Trust in government - PCA | -0.007 | 0.9323 |
| Government corruption - PCA | Trust in science | -0.418 | <0.0001 |
| Government corruption - PCA | Universal health coverage | -0.757 | <0.0001 |
| Government corruption - PCA | Universal health coverage - CMNNs | -0.697 | <0.0001 |
| Government effectiveness | Electoral democracy index | 0.631 | <0.0001 |
| Government effectiveness | Electoral populism | 0.055 | 0.4657 |

| | | | |
|---|---|---|---|
| Government effectiveness | Global health security index compliance with international norms score | 0.432 | <0.0001 |
| Government effectiveness | Global health security index detect score | 0.560 | <0.0001 |
| Government effectiveness | Global health security index health sector score | 0.695 | <0.0001 |
| Government effectiveness | Global health security index overall score | 0.758 | <0.0001 |
| Government effectiveness | Global health security index prevent score | 0.700 | <0.0001 |
| Government effectiveness | Global health security index respond score | 0.592 | <0.0001 |
| Government effectiveness | Global health security index risk environment score | 0.940 | <0.0001 |
| Government effectiveness | Government corruption - PCA | -0.911 | <0.0001 |
| Government effectiveness | Government health spending per capita | 0.784 | <0.0001 |
| Government effectiveness | Health spending per capita | 0.798 | <0.0001 |
| Government effectiveness | Healthcare Access and Quality Index | 0.808 | <0.0001 |
| Government effectiveness | Hospital beds per capita | 0.506 | <0.0001 |
| Government effectiveness | Income inequality | -0.460 | <0.0001 |
| Government effectiveness | Interpersonal trust | 0.745 | <0.0001 |
| Government effectiveness | Joint external evaluation detect score | 0.858 | <0.0001 |
| Government effectiveness | Joint external evaluation other score | 0.823 | <0.0001 |
| Government effectiveness | Joint external evaluation overall score | 0.848 | <0.0001 |
| Government effectiveness | Joint external evaluation prevent score | 0.848 | <0.0001 |
| Government effectiveness | Joint external evaluation respond score | 0.750 | <0.0001 |
| Government effectiveness | State fragility | -0.825 | <0.0001 |
| Government effectiveness | Trust in government - PCA | 0.057 | 0.5126 |
| Government effectiveness | Trust in science | 0.387 | <0.0001 |
| Government effectiveness | Universal health coverage | 0.821 | <0.0001 |
| Government effectiveness | Universal health coverage - CMNNs | 0.790 | <0.0001 |
| Government effectiveness | Universal health coverage - NCDs | 0.794 | <0.0001 |
| Government health spending per capita | Electoral democracy index | 0.566 | <0.0001 |
| Government health spending per capita | Electoral populism | 0.063 | 0.4033 |
| Government health spending per capita | Global health security index compliance with international norms score | 0.429 | <0.0001 |
| Government health spending per capita | Global health security index detect score | 0.522 | <0.0001 |

| | | | |
|---|---|---|---|
| Government health spending per capita | Global health security index health sector score | 0.697 | <0.0001 |
| Government health spending per capita | Global health security index overall score | 0.705 | <0.0001 |
| Government health spending per capita | Global health security index prevent score | 0.680 | <0.0001 |
| Government health spending per capita | Global health security index respond score | 0.524 | <0.0001 |
| Government health spending per capita | Global health security index risk environment score | 0.782 | <0.0001 |
| Government health spending per capita | Government corruption - PCA | -0.774 | <0.0001 |
| Government health spending per capita | Government effectiveness | 0.784 | <0.0001 |
| Government health spending per capita | Health spending per capita | 0.964 | <0.0001 |
| Government health spending per capita | Healthcare Access and Quality Index | 0.769 | <0.0001 |
| Government health spending per capita | Hospital beds per capita | 0.486 | <0.0001 |
| Government health spending per capita | Income inequality | -0.542 | <0.0001 |
| Government health spending per capita | Interpersonal trust | 0.765 | <0.0001 |
| Government health spending per capita | Joint external evaluation detect score | 0.775 | <0.0001 |
| Government health spending per capita | Joint external evaluation other score | 0.733 | <0.0001 |
| Government health spending per capita | Joint external evaluation overall score | 0.768 | <0.0001 |
| Government health spending per capita | Joint external evaluation prevent score | 0.759 | <0.0001 |
| Government health spending per capita | Joint external evaluation respond score | 0.700 | <0.0001 |
| Government health spending per capita | State fragility | -0.652 | <0.0001 |
| Government health spending per capita | Trust in government - PCA | -0.028 | 0.7479 |
| Government health spending per capita | Trust in science | 0.564 | <0.0001 |
| Government health spending per capita | Universal health coverage | 0.817 | <0.0001 |
| Government health spending per capita | Universal health coverage - CMNNs | 0.686 | <0.0001 |
| Government health spending per capita | Universal health coverage - NCDs | 0.777 | <0.0001 |
| Health spending per capita | Electoral democracy index | 0.568 | <0.0001 |
| Health spending per capita | Electoral populism | 0.083 | 0.2712 |
| Health spending per capita | Global health security index compliance with international norms score | 0.442 | <0.0001 |
| Health spending per capita | Global health security index detect score | 0.541 | <0.0001 |
| Health spending per capita | Global health security index health sector score | 0.712 | <0.0001 |
| Health spending per capita | Global health security index overall score | 0.728 | <0.0001 |
| Health spending per capita | Global health security index prevent score | 0.691 | <0.0001 |

| | | | |
|---|---|---|---|
| Health spending per capita | Global health security index respond score | 0.570 | <0.0001 |
| Health spending per capita | Global health security index risk environment score | 0.786 | <0.0001 |
| Health spending per capita | Government corruption - PCA | -0.773 | <0.0001 |
| Health spending per capita | Government effectiveness | 0.798 | <0.0001 |
| Health spending per capita | Government health spending per capita | 0.964 | <0.0001 |
| Health spending per capita | Healthcare Access and Quality Index | 0.771 | <0.0001 |
| Health spending per capita | Hospital beds per capita | 0.472 | <0.0001 |
| Health spending per capita | Income inequality | -0.515 | <0.0001 |
| Health spending per capita | Interpersonal trust | 0.730 | <0.0001 |
| Health spending per capita | Joint external evaluation detect score | 0.773 | <0.0001 |
| Health spending per capita | Joint external evaluation other score | 0.721 | <0.0001 |
| Health spending per capita | Joint external evaluation overall score | 0.759 | <0.0001 |
| Health spending per capita | Joint external evaluation prevent score | 0.749 | <0.0001 |
| Health spending per capita | Joint external evaluation respond score | 0.691 | <0.0001 |
| Health spending per capita | State fragility | -0.652 | <0.0001 |
| Health spending per capita | Trust in government - PCA | -0.023 | 0.7947 |
| Health spending per capita | Trust in science | 0.536 | <0.0001 |
| Health spending per capita | Universal health coverage | 0.811 | <0.0001 |
| Health spending per capita | Universal health coverage - CMNNs | 0.685 | <0.0001 |
| Health spending per capita | Universal health coverage - NCDs | 0.779 | <0.0001 |
| Healthcare Access and Quality Index | Electoral democracy index | 0.512 | <0.0001 |
| Healthcare Access and Quality Index | Electoral populism | 0.164 | 0.0288 |
| Healthcare Access and Quality Index | Global health security index compliance with international norms score | 0.316 | <0.0001 |
| Healthcare Access and Quality Index | Global health security index detect score | 0.510 | <0.0001 |
| Healthcare Access and Quality Index | Global health security index health sector score | 0.721 | <0.0001 |
| Healthcare Access and Quality Index | Global health security index overall score | 0.705 | <0.0001 |
| Healthcare Access and Quality Index | Global health security index prevent score | 0.689 | <0.0001 |
| Healthcare Access and Quality Index | Global health security index respond score | 0.523 | <0.0001 |
| Healthcare Access and Quality Index | Global health security index risk environment score | 0.864 | <0.0001 |
| Healthcare Access and Quality Index | Government corruption - PCA | -0.714 | <0.0001 |

| | | | |
|---|---|---|---|
| Healthcare Access and Quality Index | Government effectiveness | 0.808 | <0.0001 |
| Healthcare Access and Quality Index | Government health spending per capita | 0.769 | <0.0001 |
| Healthcare Access and Quality Index | Health spending per capita | 0.771 | <0.0001 |
| Healthcare Access and Quality Index | Hospital beds per capita | 0.626 | <0.0001 |
| Healthcare Access and Quality Index | Income inequality | -0.612 | <0.0001 |
| Healthcare Access and Quality Index | Interpersonal trust | 0.621 | <0.0001 |
| Healthcare Access and Quality Index | Joint external evaluation detect score | 0.839 | <0.0001 |
| Healthcare Access and Quality Index | Joint external evaluation other score | 0.856 | <0.0001 |
| Healthcare Access and Quality Index | Joint external evaluation overall score | 0.871 | <0.0001 |
| Healthcare Access and Quality Index | Joint external evaluation prevent score | 0.870 | <0.0001 |
| Healthcare Access and Quality Index | Joint external evaluation respond score | 0.793 | <0.0001 |
| Healthcare Access and Quality Index | State fragility | -0.831 | <0.0001 |
| Healthcare Access and Quality Index | Trust in government - PCA | -0.193 | 0.0241 |
| Healthcare Access and Quality Index | Trust in science | 0.367 | <0.0001 |
| Healthcare Access and Quality Index | Universal health coverage | 0.939 | <0.0001 |
| Healthcare Access and Quality Index | Universal health coverage - CMNNs | 0.944 | <0.0001 |
| Healthcare Access and Quality Index | Universal health coverage - NCDs | 0.987 | <0.0001 |
| Hospital beds per capita | Electoral democracy index | 0.382 | <0.0001 |
| Hospital beds per capita | Electoral populism | 0.155 | 0.0392 |
| Hospital beds per capita | Global health security index compliance with international norms score | 0.249 | 0.0011 |
| Hospital beds per capita | Global health security index detect score | 0.326 | <0.0001 |
| Hospital beds per capita | Global health security index health sector score | 0.445 | <0.0001 |
| Hospital beds per capita | Global health security index overall score | 0.431 | <0.0001 |
| Hospital beds per capita | Global health security index prevent score | 0.418 | <0.0001 |
| Hospital beds per capita | Global health security index respond score | 0.253 | 0.0009 |
| Hospital beds per capita | Global health security index risk environment score | 0.542 | <0.0001 |
| Hospital beds per capita | Government corruption - PCA | -0.464 | <0.0001 |
| Hospital beds per capita | Government effectiveness | 0.506 | <0.0001 |
| Hospital beds per capita | Government health spending per capita | 0.486 | <0.0001 |
| Hospital beds per capita | Health spending per capita | 0.472 | <0.0001 |

| | | | |
|---|---|---|---|
| Hospital beds per capita | Healthcare Access and Quality Index | 0.626 | <0.0001 |
| Hospital beds per capita | Income inequality | -0.567 | <0.0001 |
| Hospital beds per capita | Interpersonal trust | 0.327 | 0.0042 |
| Hospital beds per capita | Joint external evaluation detect score | 0.571 | <0.0001 |
| Hospital beds per capita | Joint external evaluation other score | 0.632 | <0.0001 |
| Hospital beds per capita | Joint external evaluation overall score | 0.601 | <0.0001 |
| Hospital beds per capita | Joint external evaluation prevent score | 0.630 | <0.0001 |
| Hospital beds per capita | Joint external evaluation respond score | 0.497 | <0.0001 |
| Hospital beds per capita | State fragility | -0.568 | <0.0001 |
| Hospital beds per capita | Trust in government - PCA | -0.103 | 0.2306 |
| Hospital beds per capita | Trust in science | 0.087 | 0.3137 |
| Hospital beds per capita | Universal health coverage | 0.533 | <0.0001 |
| Hospital beds per capita | Universal health coverage - CMNNs | 0.614 | <0.0001 |
| Hospital beds per capita | Universal health coverage - NCDs | 0.609 | <0.0001 |
| Income inequality | Electoral democracy index | -0.342 | <0.0001 |
| Income inequality | Electoral populism | -0.066 | 0.4256 |
| Income inequality | Global health security index compliance with international norms score | -0.297 | 0.0003 |
| Income inequality | Global health security index detect score | -0.276 | 0.0007 |
| Income inequality | Global health security index health sector score | -0.463 | <0.0001 |
| Income inequality | Global health security index overall score | -0.424 | <0.0001 |
| Income inequality | Global health security index prevent score | -0.438 | <0.0001 |
| Income inequality | Global health security index respond score | -0.282 | 0.0005 |
| Income inequality | Global health security index risk environment score | -0.449 | <0.0001 |
| Income inequality | Government corruption - PCA | 0.424 | <0.0001 |
| Income inequality | Government effectiveness | -0.460 | <0.0001 |
| Income inequality | Government health spending per capita | -0.542 | <0.0001 |
| Income inequality | Health spending per capita | -0.515 | <0.0001 |
| Income inequality | Healthcare Access and Quality Index | -0.612 | <0.0001 |
| Income inequality | Hospital beds per capita | -0.567 | <0.0001 |
| Income inequality | Interpersonal trust | -0.599 | <0.0001 |

| | | | |
|---|---|---|---|
| Income inequality | Joint external evaluation detect score | -0.375 | 0.0007 |
| Income inequality | Joint external evaluation other score | -0.445 | <0.0001 |
| Income inequality | Joint external evaluation overall score | -0.428 | 0.0001 |
| Income inequality | Joint external evaluation prevent score | -0.403 | 0.0002 |
| Income inequality | Joint external evaluation respond score | -0.431 | 0.0001 |
| Income inequality | State fragility | 0.412 | <0.0001 |
| Income inequality | Trust in government - PCA | -0.005 | 0.9506 |
| Income inequality | Trust in science | -0.304 | 0.0004 |
| Income inequality | Universal health coverage | -0.539 | <0.0001 |
| Income inequality | Universal health coverage - CMNNs | -0.551 | <0.0001 |
| Income inequality | Universal health coverage - NCDs | -0.601 | <0.0001 |
| Interpersonal trust | Electoral democracy index | 0.416 | 0.0002 |
| Interpersonal trust | Electoral populism | -0.133 | 0.2536 |
| Interpersonal trust | Global health security index compliance with international norms score | 0.312 | 0.0073 |
| Interpersonal trust | Global health security index detect score | 0.279 | 0.0169 |
| Interpersonal trust | Global health security index health sector score | 0.561 | <0.0001 |
| Interpersonal trust | Global health security index overall score | 0.547 | <0.0001 |
| Interpersonal trust | Global health security index prevent score | 0.509 | <0.0001 |
| Interpersonal trust | Global health security index respond score | 0.473 | <0.0001 |
| Interpersonal trust | Global health security index risk environment score | 0.702 | <0.0001 |
| Interpersonal trust | Government corruption - PCA | -0.727 | <0.0001 |
| Interpersonal trust | Government effectiveness | 0.745 | <0.0001 |
| Interpersonal trust | Government health spending per capita | 0.765 | <0.0001 |
| Interpersonal trust | Health spending per capita | 0.730 | <0.0001 |
| Interpersonal trust | Healthcare Access and Quality Index | 0.621 | <0.0001 |
| Interpersonal trust | Hospital beds per capita | 0.327 | 0.0042 |
| Interpersonal trust | Income inequality | -0.599 | <0.0001 |
| Interpersonal trust | Joint external evaluation detect Score | 0.732 | <0.0001 |
| Interpersonal trust | Joint external evaluation other score | 0.706 | <0.0001 |
| Interpersonal trust | Joint external evaluation overall score | 0.758 | <0.0001 |

| | | | |
|---|---|---|---|
| Interpersonal trust | Joint external evaluation prevent score | 0.732 | <0.0001 |
| Interpersonal trust | Joint external evaluation respond score | 0.703 | <0.0001 |
| Interpersonal trust | State fragility | -0.482 | <0.0001 |
| Interpersonal trust | Trust in government - PCA | 0.355 | 0.0018 |
| Interpersonal trust | Trust in science | 0.549 | <0.0001 |
| Interpersonal trust | Universal health coverage | 0.630 | <0.0001 |
| Interpersonal trust | Universal health coverage - CMNNs | 0.544 | <0.0001 |
| Interpersonal trust | Universal health coverage - NCDs | 0.596 | <0.0001 |
| Joint external evaluation detect Score | Electoral democracy index | 0.359 | 0.0006 |
| Joint external evaluation detect Score | Electoral populism | 0.109 | 0.3121 |
| Joint external evaluation detect Score | Global health security index compliance with international norms score | 0.517 | <0.0001 |
| Joint external evaluation detect Score | Global health security index detect score | 0.702 | <0.0001 |
| Joint external evaluation detect Score | Global health security index health sector score | 0.796 | <0.0001 |
| Joint external evaluation detect Score | Global health security index overall score | 0.849 | <0.0001 |
| Joint external evaluation detect Score | Global health security index prevent score | 0.813 | <0.0001 |
| Joint external evaluation detect Score | Global health security index respond score | 0.722 | <0.0001 |
| Joint external evaluation detect Score | Global health security index risk environment score | 0.842 | <0.0001 |
| Joint external evaluation detect Score | Government corruption - PCA | -0.767 | <0.0001 |
| Joint external evaluation detect Score | Government effectiveness | 0.858 | <0.0001 |
| Joint external evaluation detect Score | Government health spending per capita | 0.775 | <0.0001 |
| Joint external evaluation detect Score | Health spending per capita | 0.773 | <0.0001 |
| Joint external evaluation detect Score | Healthcare Access and Quality Index | 0.839 | <0.0001 |
| Joint external evaluation detect Score | Hospital beds per capita | 0.571 | <0.0001 |
| Joint external evaluation detect Score | Income inequality | -0.375 | 0.0007 |
| Joint external evaluation detect Score | Interpersonal trust | 0.732 | <0.0001 |
| Joint external evaluation detect Score | Joint external evaluation other score | 0.873 | <0.0001 |
| Joint external evaluation detect Score | Joint external evaluation overall score | 0.946 | <0.0001 |
| Joint external evaluation detect Score | Joint external evaluation prevent score | 0.940 | <0.0001 |
| Joint external evaluation detect Score | Joint external evaluation respond score | 0.847 | <0.0001 |
| Joint external evaluation detect Score | State fragility | -0.721 | <0.0001 |

| | | | |
|---|---|---|---|
| Joint external evaluation detect Score | Trust in government - PCA | -0.035 | 0.7767 |
| Joint external evaluation detect Score | Trust in science | 0.325 | 0.0053 |
| Joint external evaluation detect Score | Universal health coverage | 0.842 | <0.0001 |
| Joint external evaluation detect Score | Universal health coverage - CMNNs | 0.806 | <0.0001 |
| Joint external evaluation detect Score | Universal health coverage - NCDs | 0.831 | <0.0001 |
| Joint external evaluation other score | Electoral democracy index | 0.359 | 0.0006 |
| Joint external evaluation other score | Electoral populism | 0.092 | 0.3930 |
| Joint external evaluation other score | Global health security index compliance with international norms score | 0.447 | <0.0001 |
| Joint external evaluation other score | Global health security index detect score | 0.663 | <0.0001 |
| Joint external evaluation other score | Global health security index health sector score | 0.796 | <0.0001 |
| Joint external evaluation other score | Global health security index overall score | 0.815 | <0.0001 |
| Joint external evaluation other score | Global health security index prevent score | 0.775 | <0.0001 |
| Joint external evaluation other score | Global health security index respond score | 0.699 | <0.0001 |
| Joint external evaluation other score | Global health security index risk environment score | 0.830 | <0.0001 |
| Joint external evaluation other score | Government corruption - PCA | -0.690 | <0.0001 |
| Joint external evaluation other score | Government effectiveness | 0.823 | <0.0001 |
| Joint external evaluation other score | Government health spending per capita | 0.733 | <0.0001 |
| Joint external evaluation other score | Health spending per capita | 0.721 | <0.0001 |
| Joint external evaluation other score | Healthcare Access and Quality Index | 0.856 | <0.0001 |
| Joint external evaluation other score | Hospital beds per capita | 0.632 | <0.0001 |
| Joint external evaluation other score | Income inequality | -0.445 | <0.0001 |
| Joint external evaluation other score | Interpersonal trust | 0.706 | <0.0001 |
| Joint external evaluation other score | Joint external evaluation detect score | 0.873 | <0.0001 |
| Joint external evaluation other score | Joint external evaluation overall score | 0.957 | <0.0001 |
| Joint external evaluation other score | Joint external evaluation prevent score | 0.912 | <0.0001 |
| Joint external evaluation other score | Joint external evaluation respond score | 0.913 | <0.0001 |
| Joint external evaluation other score | State fragility | -0.740 | <0.0001 |
| Joint external evaluation other score | Trust in government - PCA | -0.127 | 0.2937 |
| Joint external evaluation other score | Trust in science | 0.306 | 0.0089 |
| Joint external evaluation other score | Universal health coverage | 0.813 | <0.0001 |

| | | | |
|---|---|---|---|
| Joint external evaluation other score | Universal health coverage - CMNNs | 0.807 | <0.0001 |
| Joint external evaluation other score | Universal health coverage - NCDs | 0.837 | <0.0001 |
| Joint external evaluation overall score | Electoral democracy index | 0.335 | 0.0014 |
| Joint external evaluation overall score | Electoral populism | 0.094 | 0.3861 |
| Joint external evaluation overall score | Global health security index compliance with international norms score | 0.486 | <0.0001 |
| Joint external evaluation overall score | Global health security index detect score | 0.670 | <0.0001 |
| Joint external evaluation overall score | Global health security index health sector score | 0.811 | <0.0001 |
| Joint external evaluation overall score | Global health security index overall score | 0.844 | <0.0001 |
| Joint external evaluation overall score | Global health security index prevent score | 0.818 | <0.0001 |
| Joint external evaluation overall score | Global health security index respond score | 0.737 | <0.0001 |
| Joint external evaluation overall score | Global health security index risk environment score | 0.851 | <0.0001 |
| Joint external evaluation overall score | Government corruption - PCA | -0.749 | <0.0001 |
| Joint external evaluation overall score | Government effectiveness | 0.848 | <0.0001 |
| Joint external evaluation overall score | Government health spending per capita | 0.768 | <0.0001 |
| Joint external evaluation overall score | Health spending per capita | 0.759 | <0.0001 |
| Joint external evaluation overall score | Healthcare Access and Quality Index | 0.871 | <0.0001 |
| Joint external evaluation overall score | Hospital beds per capita | 0.601 | <0.0001 |
| Joint external evaluation overall score | Income inequality | -0.428 | 0.0001 |
| Joint external evaluation overall score | Interpersonal trust | 0.758 | <0.0001 |
| Joint external evaluation overall score | Joint external evaluation detect score | 0.946 | <0.0001 |
| Joint external evaluation overall score | Joint external evaluation other score | 0.957 | <0.0001 |
| Joint external evaluation overall score | Joint external evaluation prevent score | 0.978 | <0.0001 |
| Joint external evaluation overall score | Joint external evaluation respond score | 0.955 | <0.0001 |
| Joint external evaluation overall score | State fragility | -0.744 | <0.0001 |
| Joint external evaluation overall score | Trust in government - PCA | -0.079 | 0.5153 |
| Joint external evaluation overall score | Trust in science | 0.322 | 0.0058 |
| Joint external evaluation overall score | Universal health coverage | 0.851 | <0.0001 |
| Joint external evaluation overall score | Universal health coverage - CMNNs | 0.837 | <0.0001 |
| Joint external evaluation overall score | Universal health coverage - NCDs | 0.859 | <0.0001 |
| Joint external evaluation prevent score | Electoral democracy index | 0.343 | 0.0011 |

| | | | |
|---|---|---|---|
| Joint external evaluation prevent score | Electoral populism | 0.070 | 0.5189 |
| Joint external evaluation prevent score | Global health security index compliance with international norms score | 0.495 | <0.0001 |
| Joint external evaluation prevent score | Global health security index detect score | 0.683 | <0.0001 |
| Joint external evaluation prevent score | Global health security index health sector score | 0.790 | <0.0001 |
| Joint external evaluation prevent score | Global health security index overall score | 0.842 | <0.0001 |
| Joint external evaluation prevent score | Global health security index prevent score | 0.824 | <0.0001 |
| Joint external evaluation prevent score | Global health security index respond score | 0.717 | <0.0001 |
| Joint external evaluation prevent score | Global health security index risk environment score | 0.852 | <0.0001 |
| Joint external evaluation prevent score | Government corruption - PCA | -0.755 | <0.0001 |
| Joint external evaluation prevent score | Government effectiveness | 0.848 | <0.0001 |
| Joint external evaluation prevent score | Government health spending per capita | 0.759 | <0.0001 |
| Joint external evaluation prevent score | Health spending per capita | 0.749 | <0.0001 |
| Joint external evaluation prevent score | Healthcare Access and Quality Index | 0.870 | <0.0001 |
| Joint external evaluation prevent score | Hospital beds per capita | 0.630 | <0.0001 |
| Joint external evaluation prevent score | Income inequality | -0.403 | 0.0002 |
| Joint external evaluation prevent score | Interpersonal trust | 0.732 | <0.0001 |
| Joint external evaluation prevent score | Joint external evaluation detect score | 0.940 | <0.0001 |
| Joint external evaluation prevent score | Joint external evaluation other score | 0.912 | <0.0001 |
| Joint external evaluation prevent score | Joint external evaluation overall score | 0.978 | <0.0001 |
| Joint external evaluation prevent score | Joint external evaluation respond score | 0.887 | <0.0001 |
| Joint external evaluation prevent score | State fragility | -0.749 | <0.0001 |
| Joint external evaluation prevent score | Trust in government - PCA | -0.072 | 0.5553 |
| Joint external evaluation prevent score | Trust in science | 0.265 | 0.0242 |
| Joint external evaluation prevent score | Universal health coverage | 0.864 | <0.0001 |
| Joint external evaluation prevent score | Universal health coverage - CMNNs | 0.846 | <0.0001 |
| Joint external evaluation prevent score | Universal health coverage - NCDs | 0.860 | <0.0001 |
| Joint external evaluation respond score | Electoral democracy index | 0.259 | 0.0149 |
| Joint external evaluation respond score | Electoral populism | 0.098 | 0.3653 |
| Joint external evaluation respond score | Global health security index compliance with international norms score | 0.416 | 0.0001 |

| | | | |
|---|---|---|---|
| Joint external evaluation respond score | Global health security index detect score | 0.555 | <0.0001 |
| Joint external evaluation respond score | Global health security index health sector score | 0.749 | <0.0001 |
| Joint external evaluation respond score | Global health security index overall score | 0.751 | <0.0001 |
| Joint external evaluation respond score | Global health security index prevent score | 0.731 | <0.0001 |
| Joint external evaluation respond score | Global health security index respond score | 0.697 | <0.0001 |
| Joint external evaluation respond score | Global health security index risk environment score | 0.761 | <0.0001 |
| Joint external evaluation respond score | Government corruption - PCA | -0.672 | <0.0001 |
| Joint external evaluation respond score | Government effectiveness | 0.750 | <0.0001 |
| Joint external evaluation respond score | Government health spending per capita | 0.700 | <0.0001 |
| Joint external evaluation respond score | Health spending per capita | 0.691 | <0.0001 |
| Joint external evaluation respond score | Healthcare Access and Quality Index | 0.793 | <0.0001 |
| Joint external evaluation respond score | Hospital beds per capita | 0.497 | <0.0001 |
| Joint external evaluation respond score | Income inequality | -0.431 | 0.0001 |
| Joint external evaluation respond score | Interpersonal trust | 0.703 | <0.0001 |
| Joint external evaluation respond score | Joint external evaluation detect score | 0.847 | <0.0001 |
| Joint external evaluation respond score | Joint external evaluation other score | 0.913 | <0.0001 |
| Joint external evaluation respond score | Joint external evaluation overall score | 0.955 | <0.0001 |
| Joint external evaluation respond score | Joint external evaluation prevent score | 0.887 | <0.0001 |
| Joint external evaluation respond score | State fragility | -0.664 | <0.0001 |
| Joint external evaluation respond score | Trust in government - PCA | -0.085 | 0.4824 |
| Joint external evaluation respond score | Trust in science | 0.369 | 0.0014 |
| Joint external evaluation respond score | Universal health coverage | 0.758 | <0.0001 |
| Joint external evaluation respond score | Universal health coverage - CMNNs | 0.760 | <0.0001 |
| Joint external evaluation respond score | Universal health coverage - NCDs | 0.781 | <0.0001 |
| State fragility | Electoral democracy index | -0.630 | <0.0001 |
| State fragility | Electoral populism | -0.116 | 0.1464 |
| State fragility | Global health security index compliance with international norms score | -0.345 | <0.0001 |
| State fragility | Global health security index detect score | -0.537 | <0.0001 |
| State fragility | Global health security index health sector score | -0.653 | <0.0001 |
| State fragility | Global health security index overall score | -0.691 | <0.0001 |

| | | | |
|---|---|---|---|
| State fragility | Global health security index prevent score | -0.636 | <0.0001 |
| State fragility | Global health security index respond score | -0.526 | <0.0001 |
| State fragility | Global health security index risk environment score | -0.892 | <0.0001 |
| State fragility | Government corruption - PCA | 0.747 | <0.0001 |
| State fragility | Government effectiveness | -0.825 | <0.0001 |
| State fragility | Government health spending per capita | -0.652 | <0.0001 |
| State fragility | Health spending per capita | -0.652 | <0.0001 |
| State fragility | Healthcare Access and Quality Index | -0.831 | <0.0001 |
| State fragility | Hospital beds per capita | -0.568 | <0.0001 |
| State fragility | Income inequality | 0.412 | <0.0001 |
| State fragility | Interpersonal trust | -0.482 | <0.0001 |
| State fragility | Joint external evaluation detect score | -0.721 | <0.0001 |
| State fragility | Joint external evaluation other score | -0.740 | <0.0001 |
| State fragility | Joint external evaluation overall score | -0.744 | <0.0001 |
| State fragility | Joint external evaluation prevent score | -0.749 | <0.0001 |
| State fragility | Joint external evaluation respond score | -0.664 | <0.0001 |
| State fragility | Trust in government - PCA | 0.228 | 0.0085 |
| State fragility | Trust in science | -0.208 | 0.0158 |
| State fragility | Universal health coverage | -0.785 | <0.0001 |
| State fragility | Universal health coverage - CMNNs | -0.841 | <0.0001 |
| State fragility | Universal health coverage - NCDs | -0.807 | <0.0001 |
| Trust in government - PCA | Electoral democracy index | -0.317 | 0.0002 |
| Trust in government - PCA | Electoral populism | -0.082 | 0.3447 |
| Trust in government - PCA | Global health security index compliance with international norms score | 0.043 | 0.6209 |
| Trust in government - PCA | Global health security index detect score | -0.123 | 0.1576 |
| Trust in government - PCA | Global health security index health sector score | -0.086 | 0.3206 |
| Trust in government - PCA | Global health security index overall score | -0.065 | 0.4568 |
| Trust in government - PCA | Global health security index prevent score | -0.062 | 0.4765 |
| Trust in government - PCA | Global health security index respond score | 0.025 | 0.7721 |
| Trust in government - PCA | Global health security index risk environment score | -0.068 | 0.4327 |

| | | | |
|---|---|---|---|
| Trust in government - PCA | Government corruption - PCA | -0.007 | 0.9323 |
| Trust in government - PCA | Government effectiveness | 0.057 | 0.5126 |
| Trust in government - PCA | Government health spending per capita | -0.028 | 0.7479 |
| Trust in government - PCA | Health spending per capita | -0.023 | 0.7947 |
| Trust in government - PCA | Healthcare Access and Quality Index | -0.193 | 0.0241 |
| Trust in government - PCA | Hospital beds per capita | -0.103 | 0.2306 |
| Trust in government - PCA | Income inequality | -0.005 | 0.9506 |
| Trust in government - PCA | Interpersonal trust | 0.355 | 0.0018 |
| Trust in government - PCA | Joint external evaluation detect score | -0.035 | 0.7767 |
| Trust in government - PCA | Joint external evaluation other score | -0.127 | 0.2937 |
| Trust in government - PCA | Joint external evaluation overall score | -0.079 | 0.5153 |
| Trust in government - PCA | Joint external evaluation prevent score | -0.072 | 0.5553 |
| Trust in government - PCA | Joint external evaluation respond score | -0.085 | 0.4824 |
| Trust in government - PCA | State fragility | 0.228 | 0.0085 |
| Trust in government - PCA | Trust in government - PCA | 1 | |
| Trust in government - PCA | Trust in science | 0.205 | 0.0205 |
| Trust in government - PCA | Universal health coverage | -0.168 | 0.0500 |
| Trust in government - PCA | Universal health coverage - CMNNs | -0.193 | 0.0242 |
| Trust in science | Electoral democracy index | 0.273 | 0.0013 |
| Trust in science | Electoral populism | -0.026 | 0.7610 |
| Trust in science | Global health security index compliance with international norms score | 0.245 | 0.0041 |
| Trust in science | Global health security index detect score | 0.157 | 0.0697 |
| Trust in science | Global health security index health sector score | 0.343 | <0.0001 |
| Trust in science | Global health security index overall score | 0.337 | 0.0001 |
| Trust in science | Global health security index prevent score | 0.359 | <0.0001 |
| Trust in science | Global health security index respond score | 0.259 | 0.0024 |
| Trust in science | Global health security index risk environment score | 0.430 | <0.0001 |
| Trust in science | Government corruption - PCA | -0.418 | <0.0001 |
| Trust in science | Government effectiveness | 0.387 | <0.0001 |
| Trust in science | Government health spending per capita | 0.564 | <0.0001 |

| | | | |
|---|---|---|---|
| Trust in science | Health spending per capita | 0.536 | <0.0001 |
| Trust in science | Healthcare Access and Quality Index | 0.367 | <0.0001 |
| Trust in science | Hospital beds per capita | 0.087 | 0.3137 |
| Trust in science | Income inequality | -0.304 | 0.0004 |
| Trust in science | Interpersonal trust | 0.549 | <0.0001 |
| Trust in science | Joint external evaluation detect score | 0.325 | 0.0053 |
| Trust in science | Joint external evaluation other score | 0.306 | 0.0089 |
| Trust in science | Joint external evaluation overall score | 0.322 | 0.0058 |
| Trust in science | Joint external evaluation prevent score | 0.265 | 0.0242 |
| Trust in science | Joint external evaluation respond score | 0.369 | 0.0014 |
| Trust in science | State fragility | 0.205 | 0.0205 |
| Trust in science | Trust in government - PCA | 0.208 | 0.0147 |
| Trust in science | Universal health coverage | 0.392 | <0.0001 |
| Trust in science | Universal health coverage - CMNNs | 0.294 | 0.0005 |
| Trust in science | Universal health coverage - NCDs | 0.350 | <0.0001 |
| Universal health coverage | Electoral democracy index | 0.544 | <0.0001 |
| Universal health coverage | Electoral populism | 0.114 | 0.1320 |
| Universal health coverage | Global health security index compliance with international norms score | 0.342 | <0.0001 |
| Universal health coverage | Global health security index detect score | 0.526 | <0.0001 |
| Universal health coverage | Global health security index health sector score | 0.731 | <0.0001 |
| Universal health coverage | Global health security index overall score | 0.723 | <0.0001 |
| Universal health coverage | Global health security index prevent score | 0.699 | <0.0001 |
| Universal health coverage | Global health security index respond score | 0.559 | <0.0001 |
| Universal health coverage | Global health security index risk environment score | 0.858 | <0.0001 |
| Universal health coverage | Government corruption - PCA | -0.757 | <0.0001 |
| Universal health coverage | Government effectiveness | 0.821 | <0.0001 |
| Universal health coverage | Government health spending per capita | 0.817 | <0.0001 |
| Universal health coverage | Health spending per capita | 0.811 | <0.0001 |
| Universal health coverage | Healthcare Access and Quality Index | 0.939 | <0.0001 |
| Universal health coverage | Hospital beds per capita | 0.533 | <0.0001 |

| | | | |
|---|---|---|---|
| Universal health coverage | Income inequality | -0.539 | <0.0001 |
| Universal health coverage | Interpersonal trust | 0.630 | <0.0001 |
| Universal health coverage | Joint external evaluation detect score | 0.842 | <0.0001 |
| Universal health coverage | Joint external evaluation other score | 0.813 | <0.0001 |
| Universal health coverage | Joint external evaluation overall score | 0.851 | <0.0001 |
| Universal health coverage | Joint external evaluation prevent score | 0.864 | <0.0001 |
| Universal health coverage | Joint external evaluation respond score | 0.758 | <0.0001 |
| Universal health coverage | State fragility | -0.785 | <0.0001 |
| Universal health coverage | Trust in government - PCA | -0.168 | 0.0500 |
| Universal health coverage | Trust in science | 0.392 | <0.0001 |
| Universal health coverage | Universal health coverage - CMNNs | 0.909 | <0.0001 |
| Universal health coverage | Universal health coverage - NCDs | 0.958 | <0.0001 |
| Universal health coverage - CMNNs | Electoral democracy index | 0.466 | <0.0001 |
| Universal health coverage - CMNNs | Electoral populism | 0.181 | 0.0157 |
| Universal health coverage - CMNNs | Global health security index compliance with international norms score | 0.272 | 0.0003 |
| Universal health coverage - CMNNs | Global health security index detect score | 0.460 | <0.0001 |
| Universal health coverage - CMNNs | Global health security index health sector score | 0.668 | <0.0001 |
| Universal health coverage - CMNNs | Global health security index overall score | 0.654 | <0.0001 |
| Universal health coverage - CMNNs | Global health security index prevent score | 0.654 | <0.0001 |
| Universal health coverage - CMNNs | Global health security index respond score | 0.469 | <0.0001 |
| Universal health coverage - CMNNs | Global health security index risk environment score | 0.852 | <0.0001 |
| Universal health coverage - CMNNs | Government corruption - PCA | -0.697 | <0.0001 |
| Universal health coverage - CMNNs | Government effectiveness | 0.790 | <0.0001 |
| Universal health coverage - CMNNs | Government health spending per capita | 0.686 | <0.0001 |
| Universal health coverage - CMNNs | Health spending per capita | 0.685 | <0.0001 |
| Universal health coverage - CMNNs | Healthcare Access and Quality Index | 0.944 | <0.0001 |
| Universal health coverage - CMNNs | Hospital beds per capita | 0.614 | <0.0001 |
| Universal health coverage - CMNNs | Income inequality | -0.551 | <0.0001 |
| Universal health coverage - CMNNs | Interpersonal trust | 0.544 | <0.0001 |
| Universal health coverage - CMNNs | Joint external evaluation detect score | 0.806 | <0.0001 |

| | | | |
|---|---|---|---|
| Universal health coverage - CMNNs | Joint external evaluation other score | 0.807 | <0.0001 |
| Universal health coverage - CMNNs | Joint external evaluation overall score | 0.837 | <0.0001 |
| Universal health coverage - CMNNs | Joint external evaluation prevent score | 0.846 | <0.0001 |
| Universal health coverage - CMNNs | Joint external evaluation respond score | 0.760 | <0.0001 |
| Universal health coverage - CMNNs | State fragility | -0.841 | <0.0001 |
| Universal health coverage - CMNNs | Trust in government - PCA | -0.193 | 0.0242 |
| Universal health coverage - CMNNs | Trust in science | 0.294 | 0.0005 |
| Universal health coverage - CMNNs | Universal health coverage | 0.909 | <0.0001 |
| Universal health coverage - CMNNs | Universal health coverage - NCDs | 0.923 | <0.0001 |
| Universal health coverage - NCDs | Electoral democracy index | 0.516 | <0.0001 |
| Universal health coverage - NCDs | Electoral populism | 0.147 | 0.0506 |
| Universal health coverage - NCDs | Global health security index compliance with international norms score | 0.319 | <0.0001 |
| Universal health coverage - NCDs | Global health security index detect score | 0.510 | <0.0001 |
| Universal health coverage - NCDs | Global health security index health sector score | 0.722 | <0.0001 |
| Universal health coverage - NCDs | Global health security index overall score | 0.703 | <0.0001 |
| Universal health coverage - NCDs | Global health security index prevent score | 0.679 | <0.0001 |
| Universal health coverage - NCDs | Global health security index respond score | 0.531 | <0.0001 |
| Universal health coverage - NCDs | Global health security index risk environment score | 0.849 | <0.0001 |
| Universal health coverage - NCDs | Government corruption - PCA | -0.707 | <0.0001 |
| Universal health coverage - NCDs | Government effectiveness | 0.794 | <0.0001 |
| Universal health coverage - NCDs | Government health spending per capita | 0.777 | <0.0001 |
| Universal health coverage - NCDs | Health spending per capita | 0.779 | <0.0001 |
| Universal health coverage - NCDs | Healthcare Access and Quality Index | 0.987 | <0.0001 |
| Universal health coverage - NCDs | Hospital beds per capita | 0.609 | <0.0001 |
| Universal health coverage - NCDs | Income inequality | -0.601 | <0.0001 |
| Universal health coverage - NCDs | Interpersonal trust | 0.596 | <0.0001 |
| Universal health coverage - NCDs | Joint external evaluation detect score | 0.831 | <0.0001 |
| Universal health coverage - NCDs | Joint external evaluation other score | 0.837 | <0.0001 |
| Universal health coverage - NCDs | Joint external evaluation overall score | 0.859 | <0.0001 |
| Universal health coverage - NCDs | Joint external evaluation prevent score | 0.860 | <0.0001 |

| | | | |
|---|---|---|---|
| Universal health coverage - NCDs | Joint external evaluation respond score | 0.781 | <0.0001 |
| Universal health coverage - NCDs | State fragility | -0.807 | <0.0001 |
| Universal health coverage - NCDs | Trust in government - PCA | -0.219 | 0.0105 |
| Universal health coverage - NCDs | Trust in science | 0.350 | <0.0001 |
| Universal health coverage - NCDs | Universal health coverage | 0.958 | <0.0001 |
| Universal health coverage - NCDs | Universal health coverage - CMNNs | 0.923 | <0.0001 |

## 2.6 Results of stage 1 and stage 2 regressions

### 2.6.1 Results of stage 1 multivariate regressions, January 1, 2020 – September 30, 2021

| Covariates | Beta estimate (uncertainty interval) | |
|---|---|---|
| | Cumulative infections per capita | IFR |
| GDP per capita | -0.17 (-0.28 - -0.05) | -0.17 (-0.28 - -0.05) |
| % of population living above 1000 people/km$^2$ | 0.05 (-0.10 – 0.19) | -0.03 (-0.16-0.06) |
| Average number of beta-coronavirus host bat species | 0.08 (-0.04 – 0.23) | 0.07 (-0.01-0.18) |
| % of population living below 100 m | -0.15 (-0.25 - -0.06) | - |
| BMI | - | 1.74 (0.65-3.2) |
| Smoking prevalence | - | 0.01 (-0.16-0.19) |
| $PM_{2.5}$ air pollution concentration (mg/m$^3$) | - | -0.03 (-0.16-0.06) |
| COPD prevalence | - | 0.04 (-0.16-0.27) |
| Cancer prevalence | - | -0.16 (-0.4-0.01) |

### 2.6.2 Results of stage 2 bivariate regressions, January 1, 2020 – September 30, 2021

| Covariates | Beta estimate (uncertainty interval) | |
|---|---|---|
| | **Cumulative infections per capita** | **IFR** |
| Hospital beds per capita | -0.07 (-0.21 - 0.06) | -0.03 (-0.11 - 0.04) |
| Government health spending per capita | -0.17 (-0.34 - 0) | -0.01 (-0.10 - 0.08) |
| Healthcare Access and Quality Index | -0.09 (-0.28 - 0.05) | -0.04 (-0.11 - 0.05) |
| Health spending per capita | -0.16 (-0.32 - -0.02) | -0.02 (-0.09 - 0.08) |
| Universal health coverage - CMNNs | -0.03 (-0.16 - 0.13) | 0.00 (-0.07 - 0.09) |
| Electoral populism | 0.12 (0.02 - 0.22) | -0.03 (-0.12 - 0.05) |
| Global health security index overall score | -0.10 (-0.26 - 0.07) | -0.03 (-0.10 - 0.04) |
| Global health security index prevent score | -0.08 (-0.24 - 0.04) | 0.01 (-0.08 - 0.12) |
| Global health security index detect score | -0.03 (-0.17 - 0.12) | -0.02 (-0.11 - 0.07) |
| Global health security index respond score | -0.12 (-0.28 - 0.03) | -0.03 (-0.13 - 0.08) |
| Global health security index health sector score | -0.11 (-0.27 - 0.07) | 0.00 (-0.11 - 0.11) |
| Global health security index compliance with international norms score | -0.08 (-0.2 - 0.08) | -0.02 (-0.12 - 0.05) |
| Global health security index risk environment score | -0.06 (-0.22 - 0.08) | -0.02 (-0.12 - 0.04) |
| Income inequality | 0.17 (0.00 - 0.31) | 0.01 (-0.09 - 0.09) |
| Government effectiveness | -0.16 (-0.34 - -0.01) | -0.02 (-0.12 - 0.06) |
| Government corruption - PCA | 0.14 (0.05 - 0.27) | 0.08 (0.02 - 0.19) |
| Trust in government - PCA | -0.25 (-0.36 - -0.11) | -0.03 (-0.12 - 0.06) |
| Interpersonal trust | -0.67 (-0.94 - -0.42) | 0.01 (-0.03 - 0.07) |
| Joint external evaluation overall score | -0.15 (-0.35 - 0.07) | -0.02 (-0.17 - 0.08) |
| Joint external evaluation prevent score | -0.16 (-0.35 - 0.08) | 0.00 (-0.07 - 0.06) |
| Joint external evaluation detect Score | -0.14 (-0.33 - 0.14) | -0.03 (-0.09 - 0.04) |
| Joint external evaluation respond score | -0.14 (-0.35 - 0.06) | -0.06 (-0.19 - 0.07) |
| Joint external evaluation other score | -0.08 (-0.28 - 0.11) | -0.05 (-0.19 - 0.07) |
| Universal health coverage - NCDs | -0.08 (-0.24 - 0.05) | -0.01 (-0.15 - 0.09) |
| State fragility | -0.01 (-0.15 - 0.14) | -0.01 (-0.13 - 0.12) |
| Trust in science | -0.11 (-0.27 - 0.05) | -0.02 (-0.12 - 0.09) |
| Universal health coverage | -0.14 (-0.29 - 0.07) | 0.01 (-0.12 - 0.18) |
| Electoral democracy index | -0.08 (-0.25 - 0.07) | 0.02 (-0.07 - 0.11) |

### 2.6.3 Results of stage 1 multivariate regressions, January 1, 2020 – October 15, 2020

| Covariates | Beta estimate (uncertainty interval) | |
|---|---|---|
| | Cumulative infections per capita | IFR |
| GDP per capita | -0.21 (-0.39 - -0.09) | -0.19 (-0.31 - 0.01) |
| % of population living above 1000 people/km$^2$ | 0.10 (-0.13 – 0.35) | -0.04 (-0.17 - 0.08) |
| Average number of beta-coronavirus host bat species | 0.01 (-0.19 – 0.22) | 0.04 (-0.09 - 0.14) |
| % of population living below 100 m | -0.24 (-0.37 - -0.09) | - |
| BMI | - | 3.36 (1.72 - 4.88) |
| Smoking prevalence | - | -0.38 (-0.64 - -0.11) |
| $PM_{2.5}$ air pollution concentration (mg/m$^3$) | - | -0.20 (-0.39 - 0.03) |
| COPD prevalence | - | 0.51 (0.19 - 0.78) |
| Cancer prevalence | - | -0.13 (-0.42 - 0.1) |

### 2.6.4 Results of stage 2 bivariate regressions, January 1, 2020 – October 15, 2020

| Covariates | Beta estimate (uncertainty interval) | |
|---|---|---|
| | Cumulative infections per capita | IFR |
| Hospital beds per capita | -0.21 (-0.41 - 0.02) | -0.03 (-0.11 - 0.04) |
| Government health spending per capita | -0.05 (-0.28 - 0.17) | -0.01 (-0.1 - 0.08) |
| Healthcare Access and Quality Index | -0.07 (-0.31 - 0.19) | -0.04 (-0.11 - 0.05) |
| Health spending per capita | -0.09 (-0.36 - 0.14) | -0.02 (-0.09 - 0.08) |
| Universal health coverage - CMNNs | -0.12 (-0.38 - 0.13) | 0.00 (-0.07 - 0.09) |
| Electoral populism | 0.15 (-0.09 - 0.37) | -0.03 (-0.12 - 0.05) |
| Global health security index overall score | -0.06 (-0.34 - 0.18) | -0.03 (-0.10 - 0.04) |
| Global health security index prevent score | -0.07 (-0.30 - 0.18) | 0.01 (-0.08 - 0.12) |
| Global health security index detect score | -0.04 (-0.33 - 0.20) | -0.02 (-0.11 - 0.07) |
| Global health security index respond score | -0.01 (-0.28 - 0.27) | -0.03 (-0.13 - 0.08) |
| Global health security index health sector score | -0.04 (-0.25 - 0.22) | 0.00 (-0.11 - 0.11) |
| Global health security index compliance with international norms score | -0.10 (-0.32 - 0.12) | -0.02 (-0.12 - 0.05) |
| Global health security index risk environment score | -0.20 (-0.44 - 0.04) | -0.02 (-0.12 - 0.04) |
| Income inequality | 0.19 (-0.05 - 0.40) | 0.01 (-0.09 - 0.09) |
| Government effectiveness | -0.24 (-0.55 - -0.05) | -0.02 (-0.12 - 0.06) |
| Government corruption - PCA | 0.24 (0.07 - 0.43) | 0.08 (0.02 - 0.19) |
| Trust in government - PCA | -0.31 (-0.51 - -0.13) | -0.03 (-0.12 - 0.06) |
| Interpersonal trust | -0.62 (-0.97 - -0.26) | 0.01 (-0.03 - 0.07) |
| Joint external evaluation overall score | -0.17 (-0.42 - 0.19) | -0.02 (-0.17 - 0.08) |
| Joint external evaluation prevent score | -0.13 (-0.52 - 0.27) | 0.00 (-0.07 - 0.06) |
| Joint external evaluation detect Score | -0.13 (-0.46 - 0.28) | -0.03 (-0.09 - 0.04) |
| Joint external evaluation respond score | -0.23 (-0.58 - 0.1) | -0.06 (-0.19 - 0.07) |
| Joint external evaluation other score | 0.06 (-0.29 - 0.34) | -0.05 (-0.19 - 0.07) |
| Universal health coverage - NCDs | -0.04 (-0.30 - 0.16) | -0.01 (-0.15 - 0.09) |
| State fragility | 0.19 (-0.02 - 0.47) | -0.01 (-0.13 - 0.12) |
| Trust in science | 0.16 (-0.08 - 0.43) | -0.02 (-0.12 - 0.09) |
| Universal health coverage | -0.10 (-0.41 - 0.13) | 0.01 (-0.12 - 0.18) |
| Electoral democracy index | -0.11 (-0.36 - 0.11) | 0.02 (-0.07 - 0.11) |

# Section 3: Additional methods

For our final analysis, we included all variables that we had access to, there was previous literature connecting, and there was theoretical perspective on how they could be included. In general, we opted for an inclusive modelling strategy and included rather than excluded variables in this exploratory analysis. A diagram of our analytic pathway follows.

Figure 3.1.1: Modelling pathway for Stages 1-3 for IFR and infections

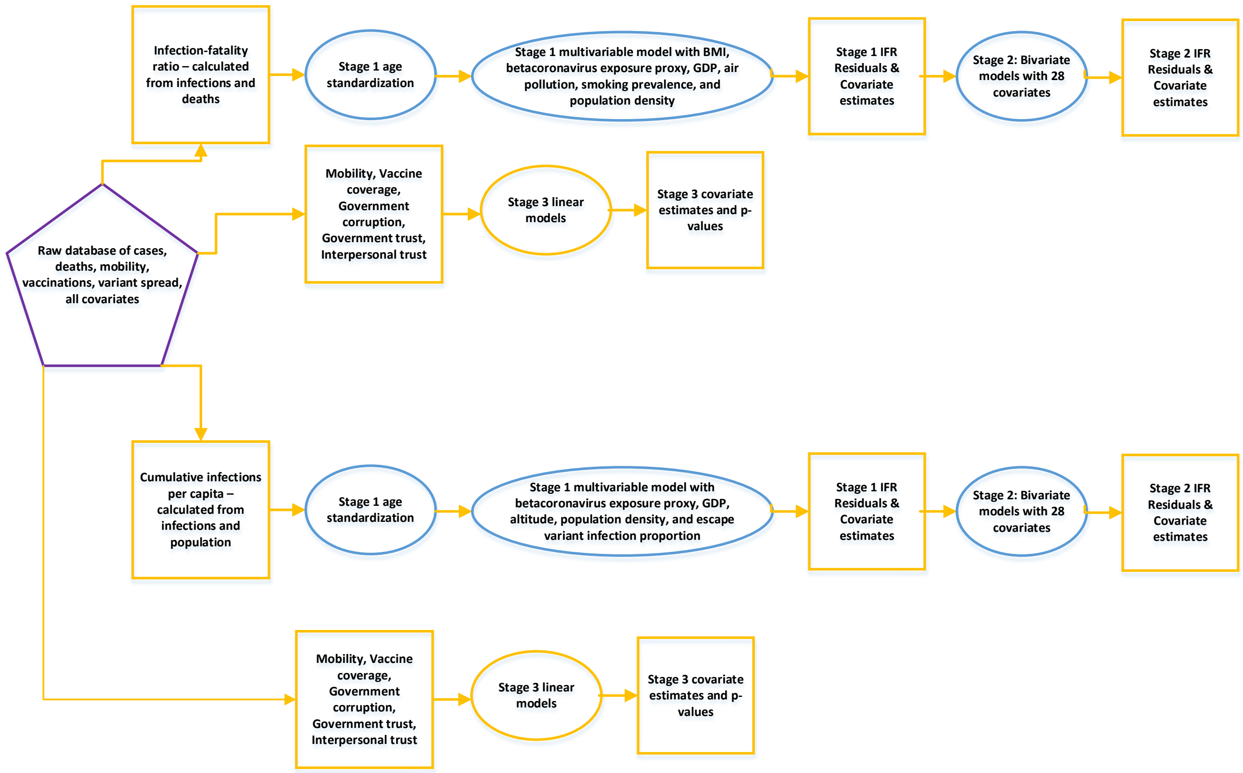


## 3.2 Proxy for previous exposure to beta coronaviruses

Literature suggests that possible previous exposure to SARS, MERS, or other beta coronaviruses may provide cross-immunity to SARS-CoV2.[23-25] In order to capture previous exposure to other beta coronaviruses, we obtained a list of possible bat hosts of beta coronaviruses produced by authors at the Verena Consortium.[18] These authors used an ensemble model of seven other statistical models (network- and trait-based models) to predict possible bat beta coronavirus hosts in addition to known bat hosts. This list of 300+ species was then compared against known species ranges, determined by experts, using the IUCN Red List of Threatened Species.[21] Of the 448 known and probable species extracted from the publication by Becker et al,[22] 441 were able to be

retrieved from the IUCN red list, allowing for possible changes in species naming convention.

Following the extraction of a geographical range for each species, each range was converted to a 1-km by 1-km raster in R using the raster package. Ranges were then layered one on top of the other, interpolated to a 5-km by 5-km grid cell range for processing times using the average of all cells in that grid. All ranges were then summed together to get the estimated number of bat species per grid cell, as seen in Figure 1 below.

Figure 3.2.1: Number of known and probable beta coronavirus bat species per grid cell

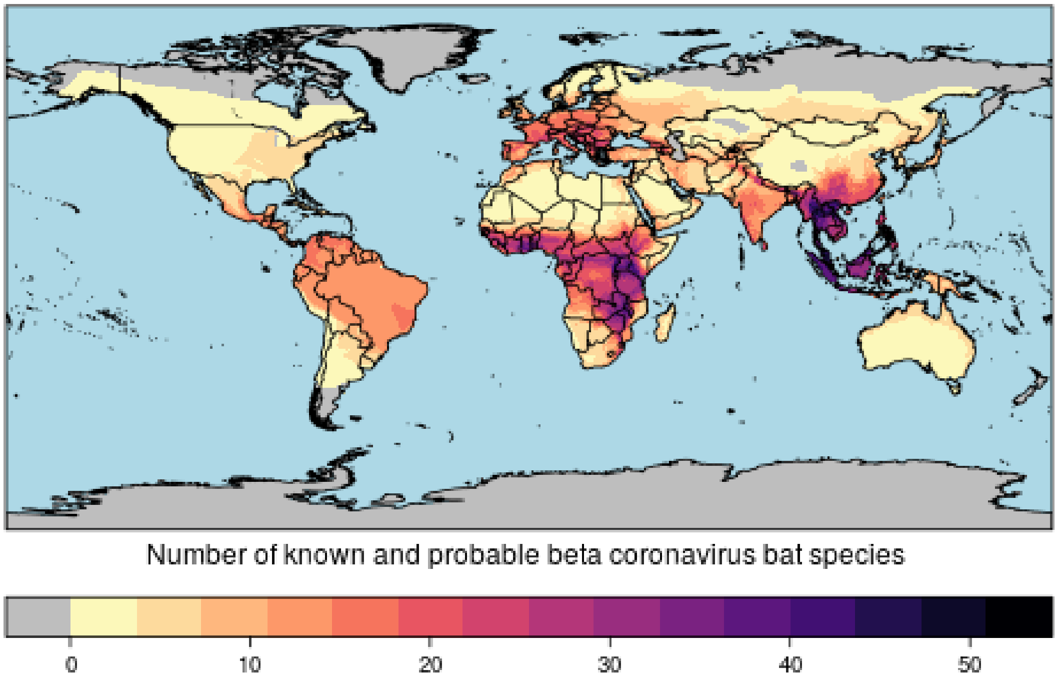


The number of species per grid cell were then averaged over the first and second administrative region in agreement with our COVID-19 units of analysis. Number of bat species per location ranged from 0 to 42, with a median exposure of 11.5 (IQR: 4.2–16.6).

## 3.3 PCA analysis for trust in government and corruption

We used principal component analysis (PCA) to create summary metrics that combine information about trust in government and/or corruption. The input variables were governmental corruption, bureaucracy corruption, trust in government (WVS), and trust in government (Gallup). The first summary metric combined the two trust covariates, and the second combined the two corruption covariates. Using centered and scaled versions of the covariates, the summary metrics consist of the first principal component from each PCA analysis. The first component (of four) explained 92.6% of the variation

for the trust variable and 94.4% of the corruption variable. PCA requires that all variables have complete data, but some of the variables had missing data. We used the imputePCA function from the missMDA R package to impute missing values, but only used observations that had at least one of the two original observations. The package implements the iterative PCA method described in Josse and Husson (2010).[26] To propagate uncertainty from the imputation process, we created 100 datasets with random realisations of the imputation.

# Section 4: Sensitivity analyses

## 4.1 Using a correction of 1% of median for log transformations

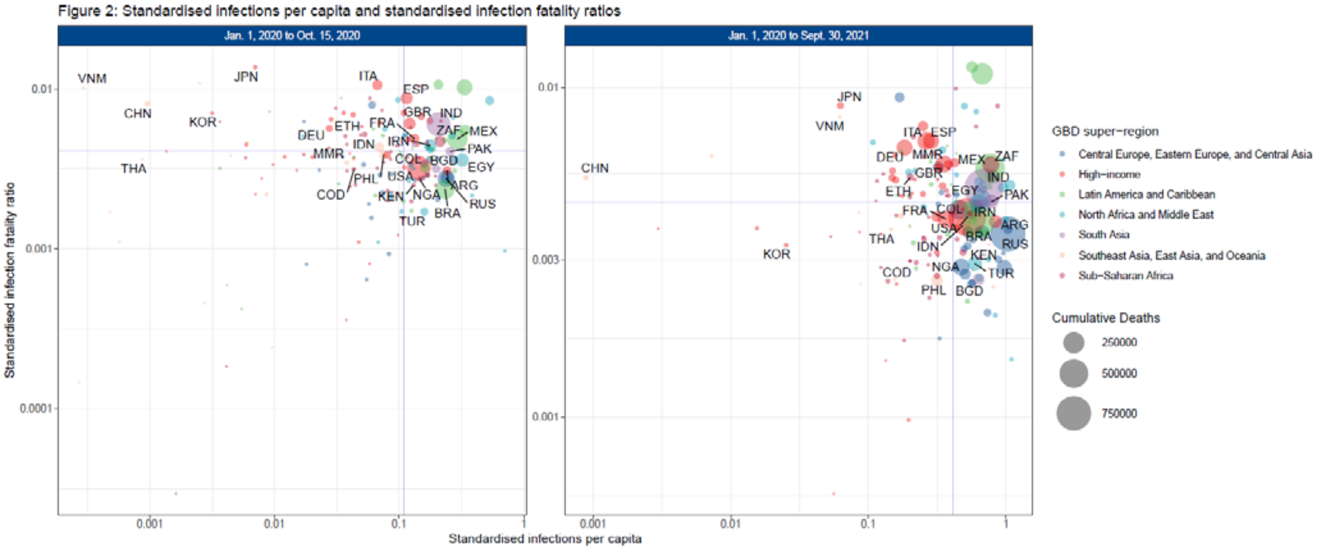


Figure 4.1.1: Infections vs. IFR

Figure 4.1.2: Bivariate results

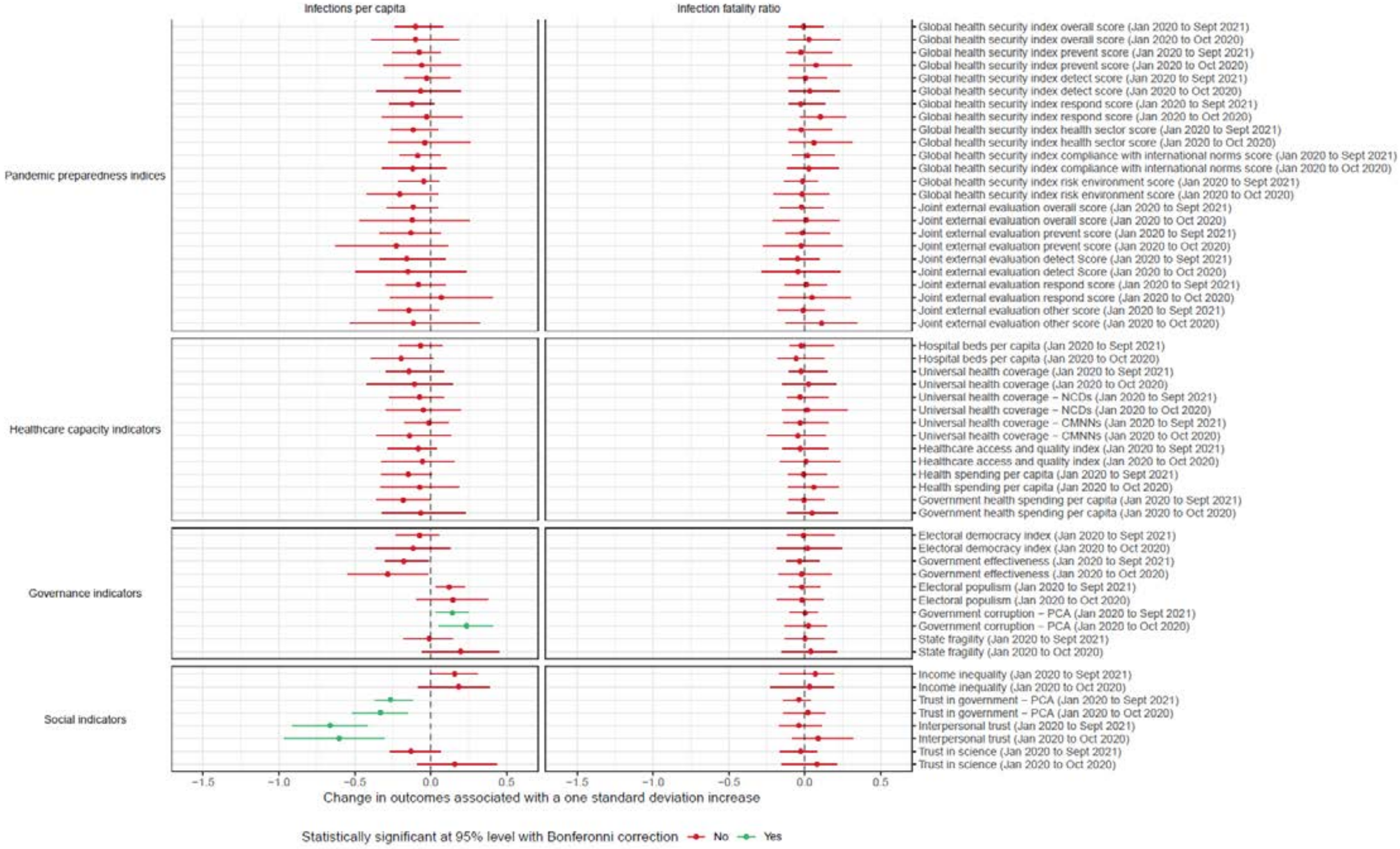

## 4.2 Using centred and scaled variables

### Figure 4.2.1: Infections vs. IFR

Figure 2: Standardised infections per capita and standardised infection fatality ratios

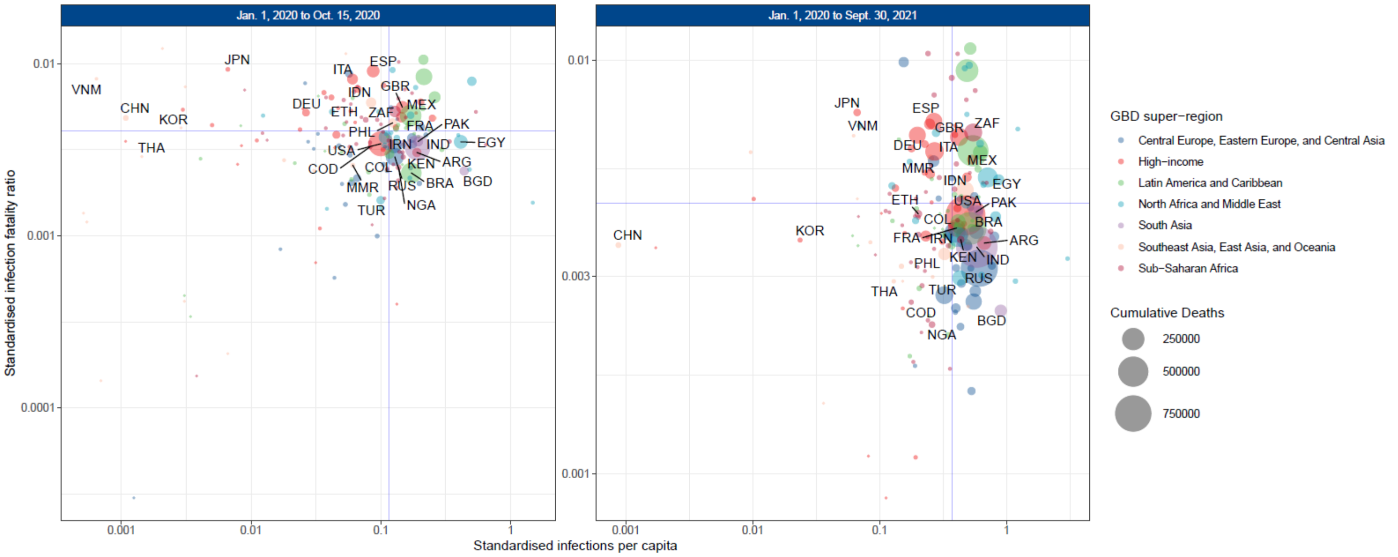


### Figure 4.2.2 Bivariate results

Figure 3: Associations between key preparedness, capacity, governance, and social indicators and infections and infection fatality ratio

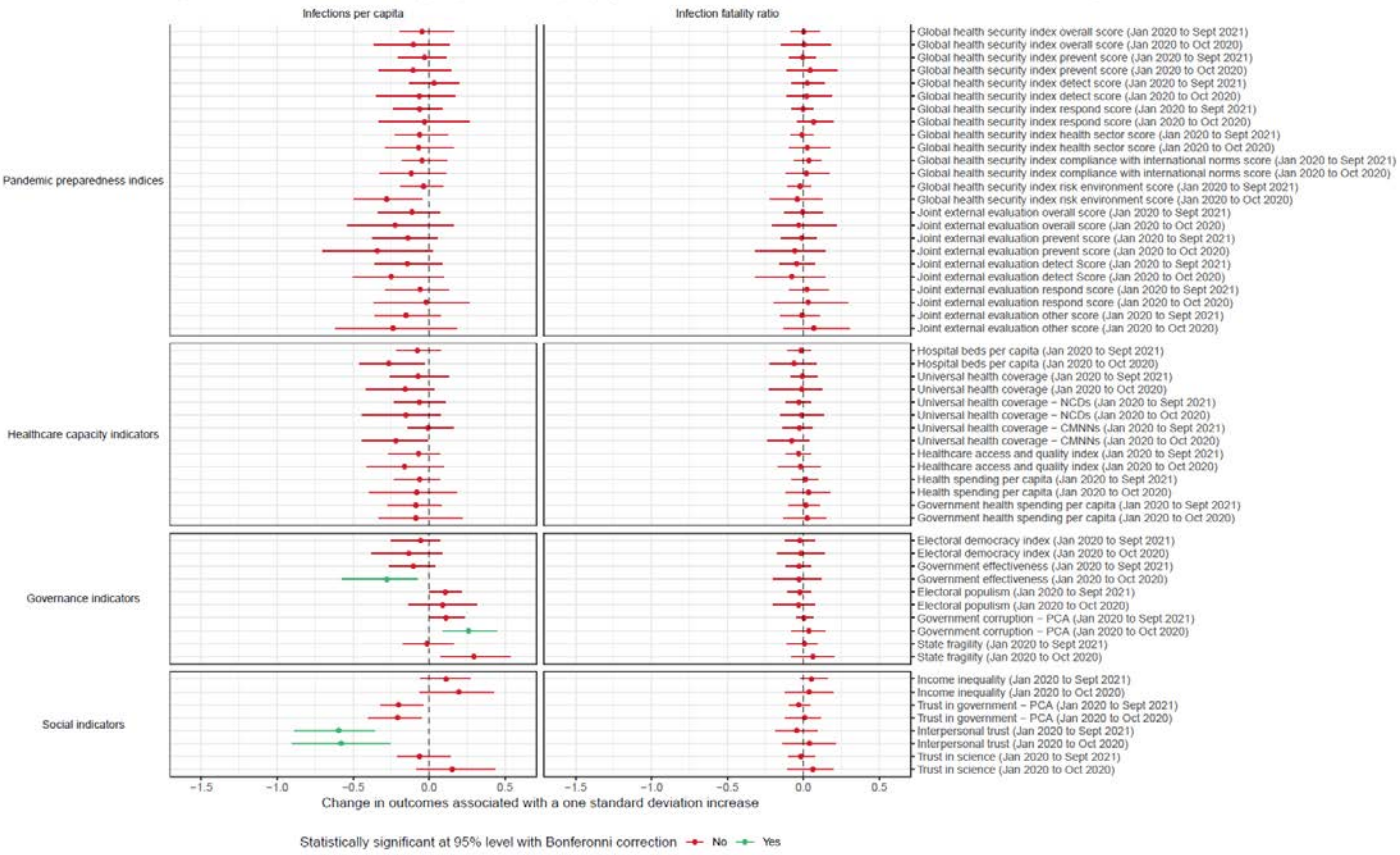

## 4.3 Including variant spread in stage 1

Figure 4.3.1: Infections vs. IFR

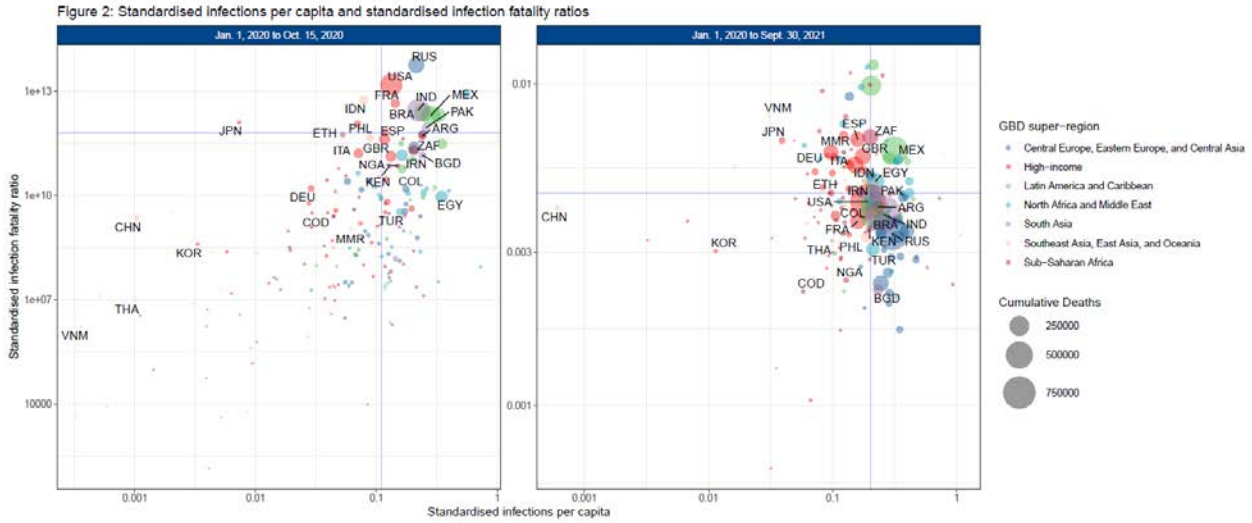


Figure 4.3.2: Bivariate results

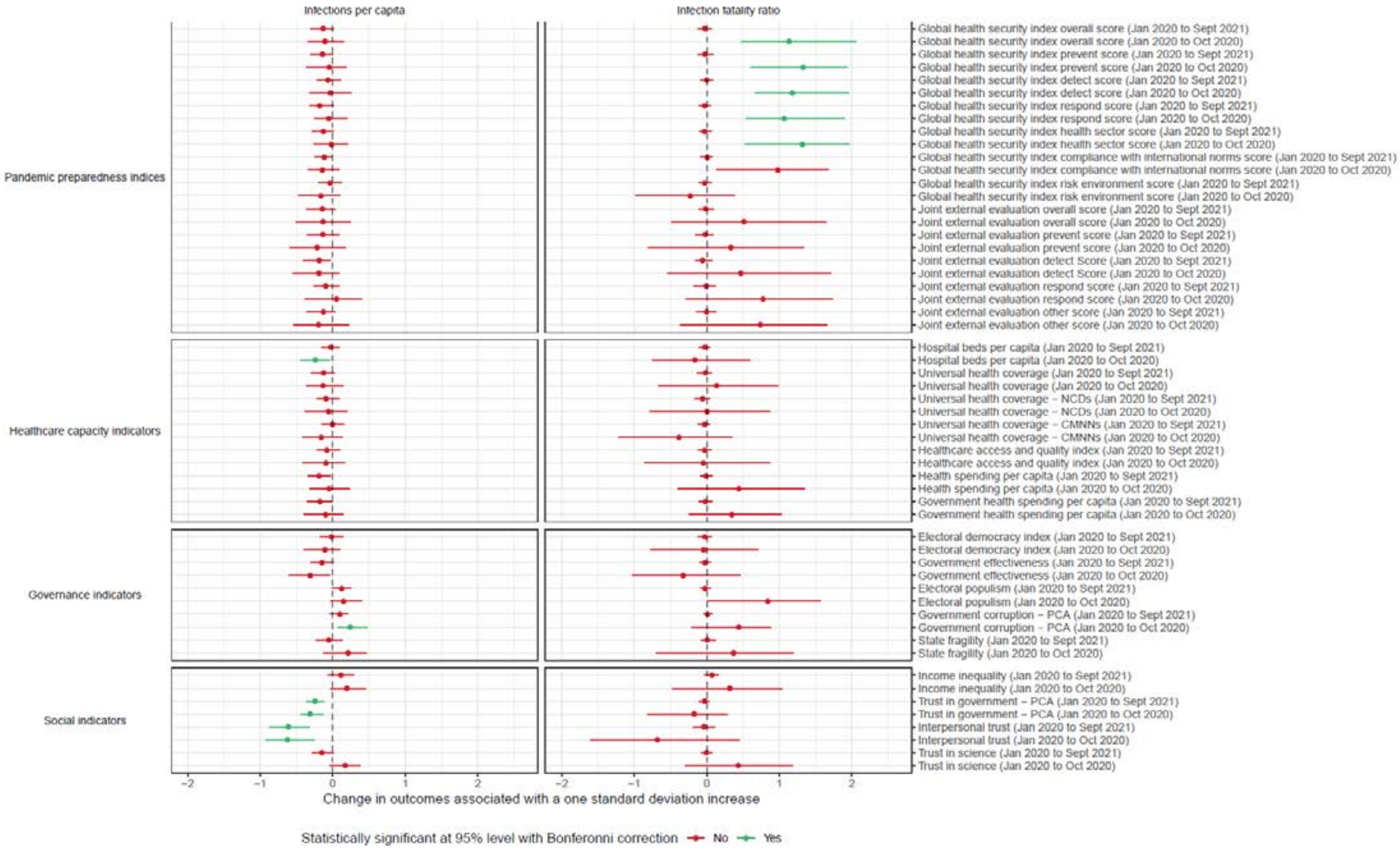

# Section 5: Supplemental results

## 5.1 Additional bivariate analyses

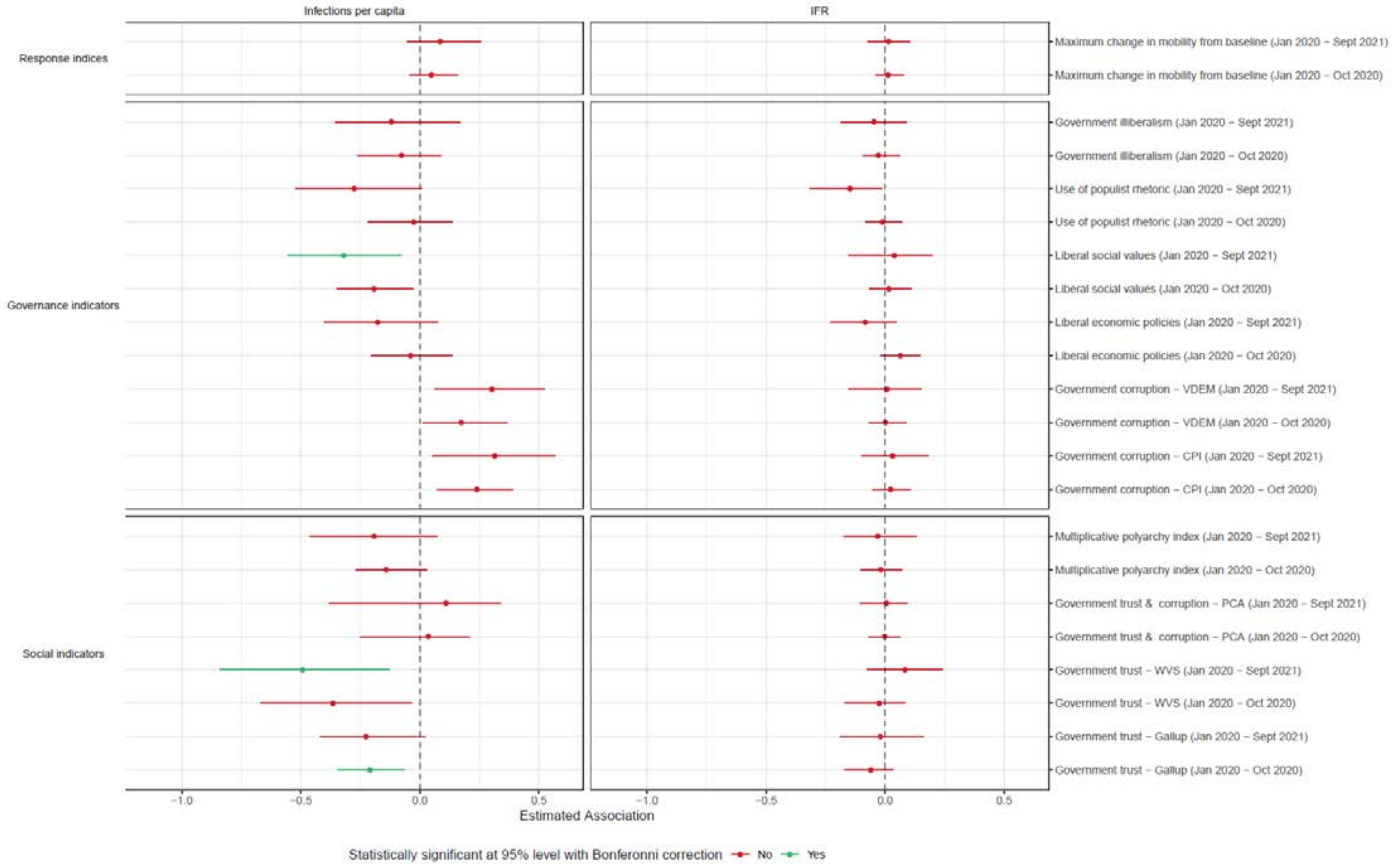

## 5.2 Additional intermediate pathway analyses looking at testing and mask use

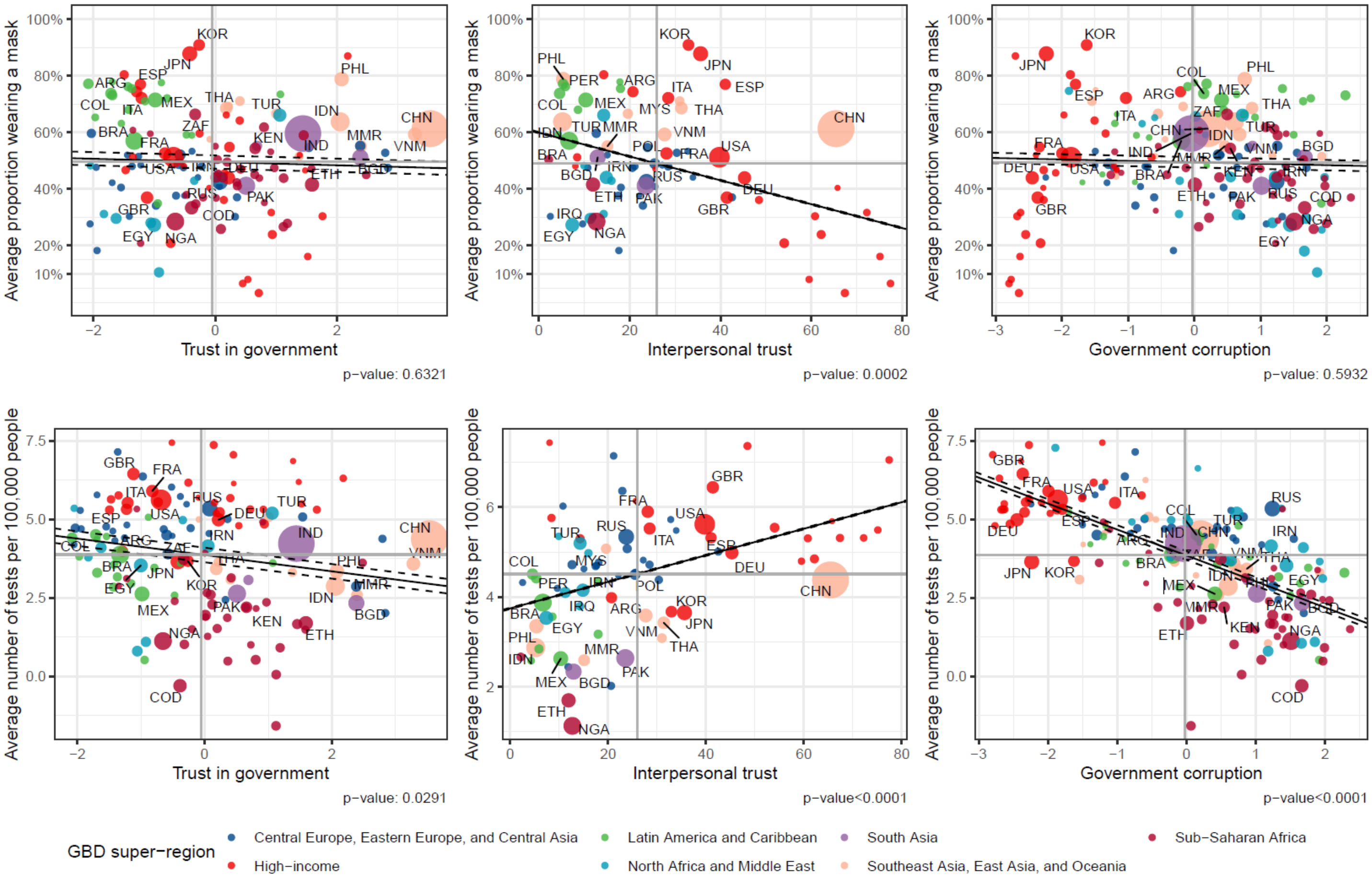

## 5.3 Additional analyses for the first phase of the pandemic (pre-variants and vaccines, January 1, 2020 – October 15, 2020)

Figure 5.3.1: Intermediate mobility pathways

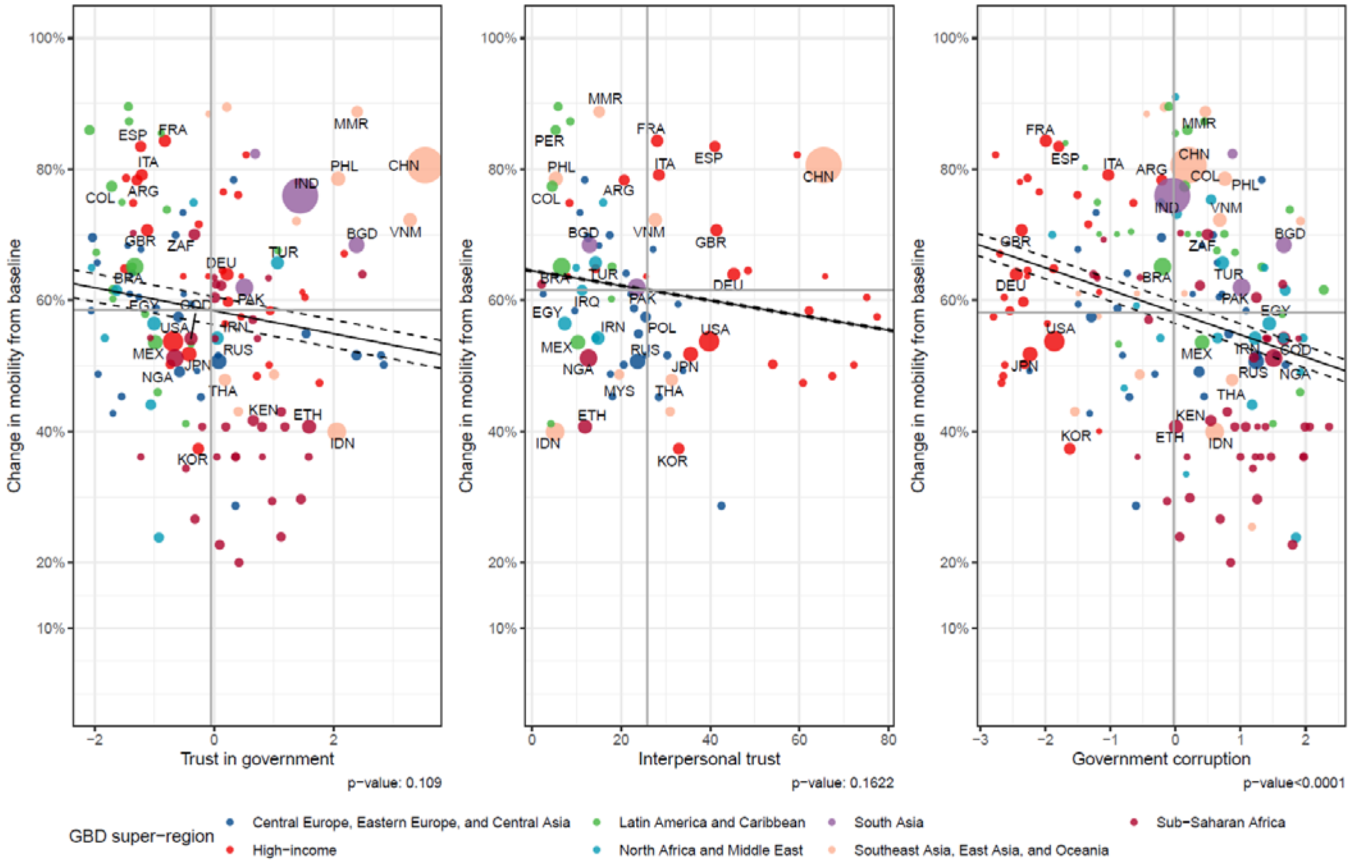

### 5.4 Analyses for seroprevalence study locations (n=303 nationals and subnationals, n=101 nationals only)

Figure 5.4.1: Seroprevalence study locations

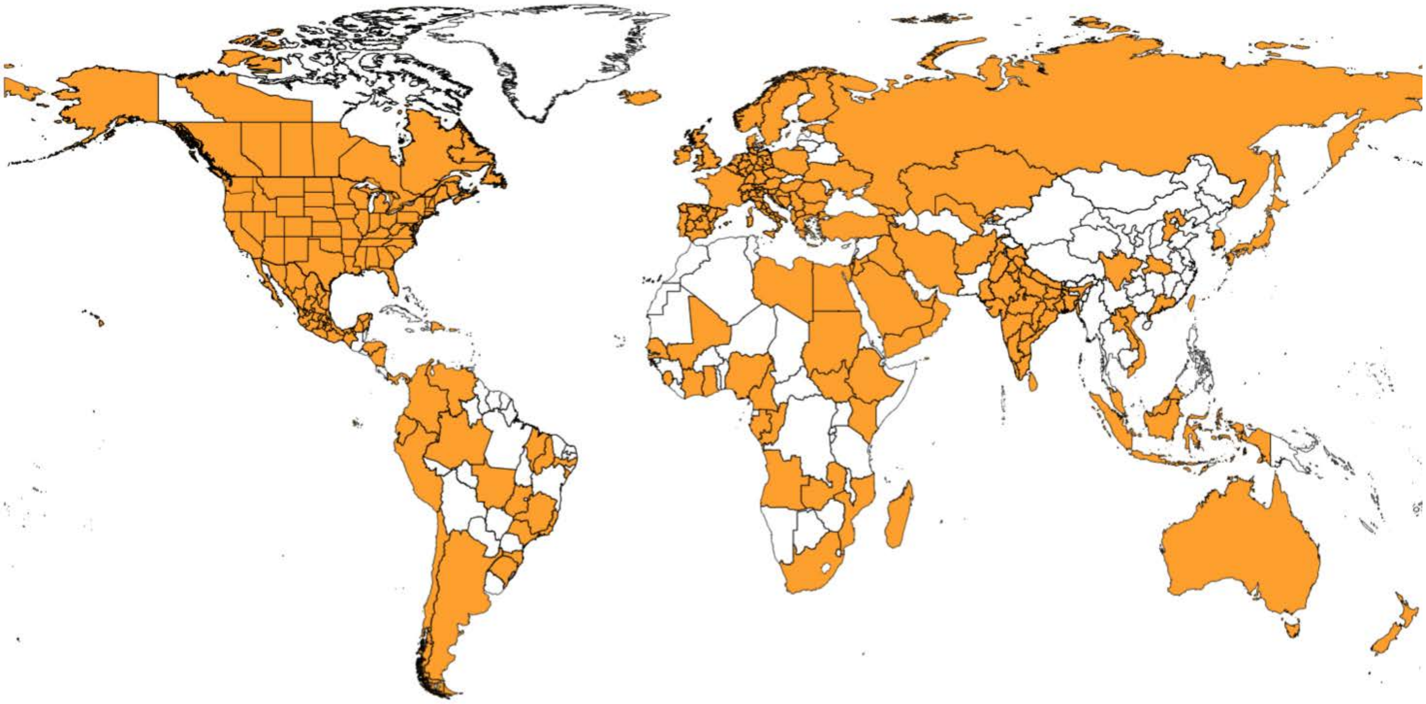

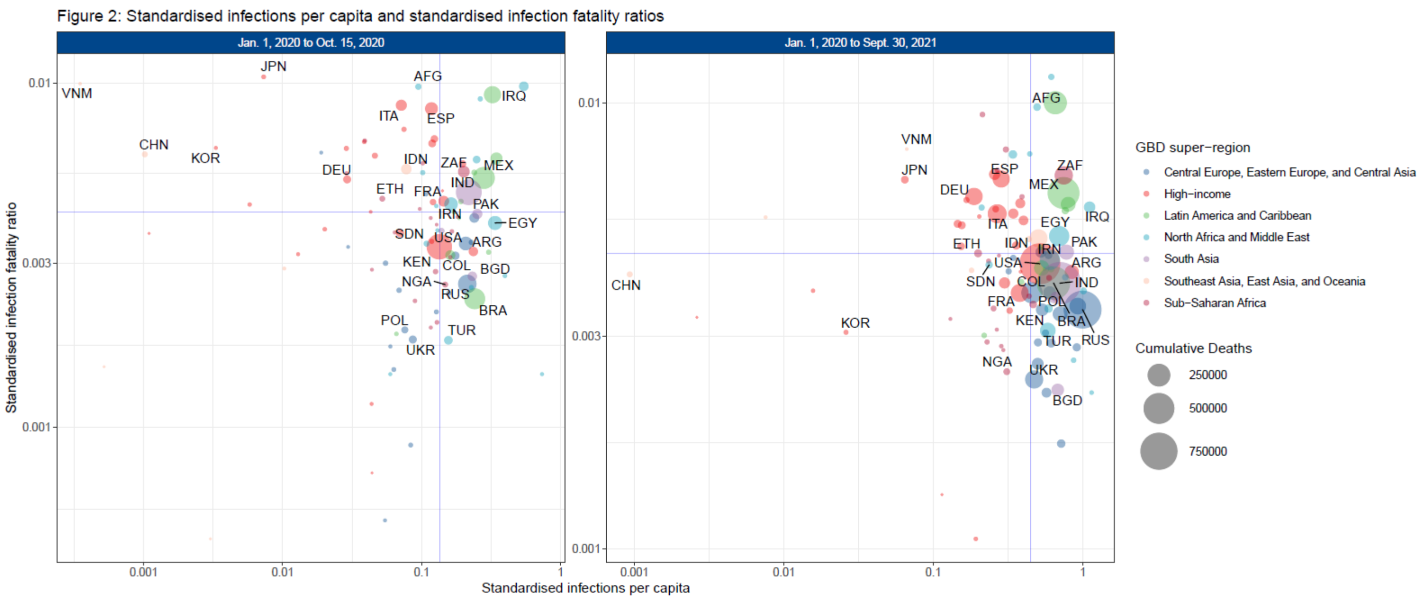


Figure 5.4.2: Infections vs. IFR

Figure 5.4.3: Intermediate pathways, vaccine coverage, and mobility change, January 1, 2020 – September 30, 2021

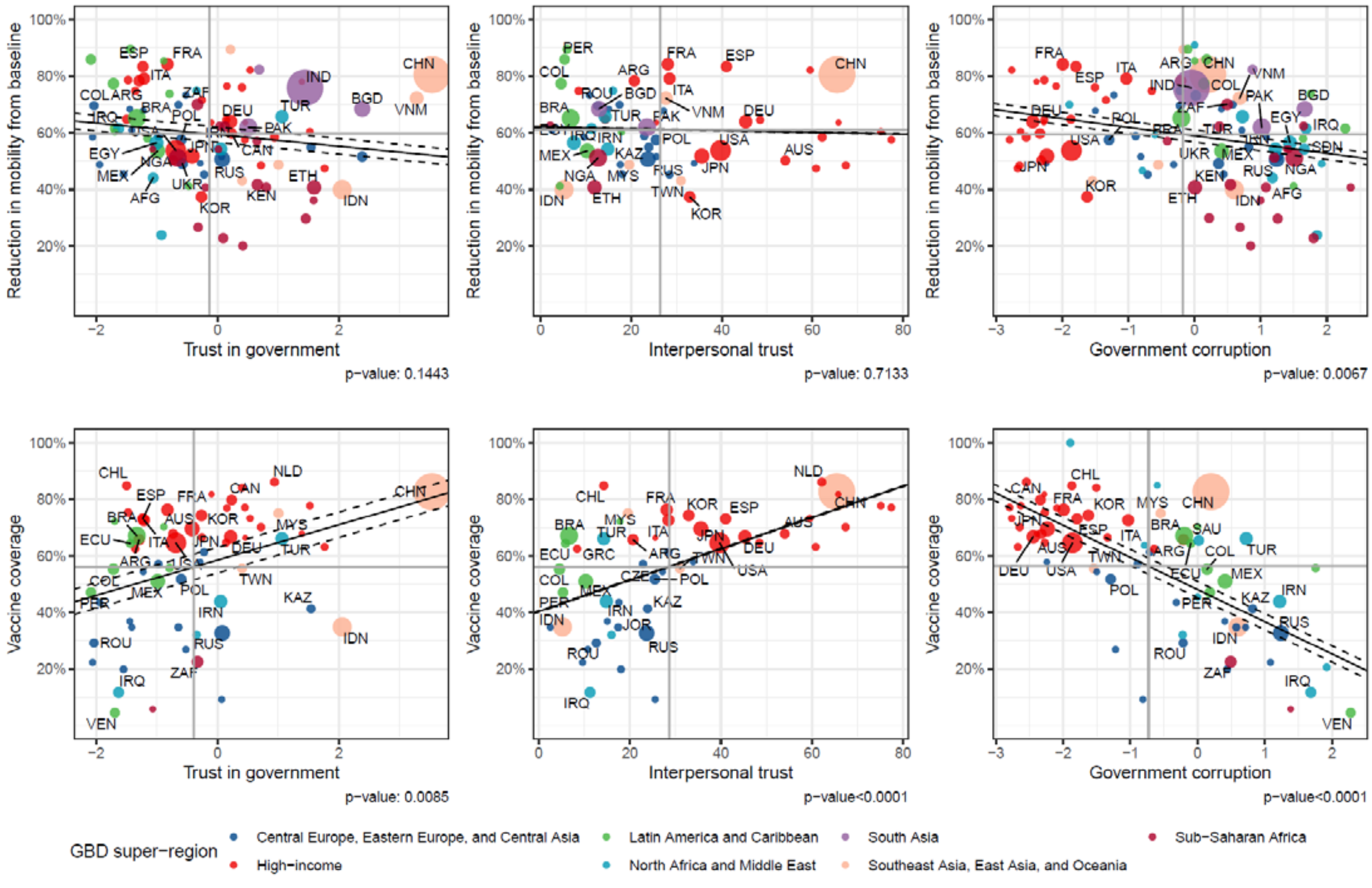

### Figure 5.4.4 Bivariate results

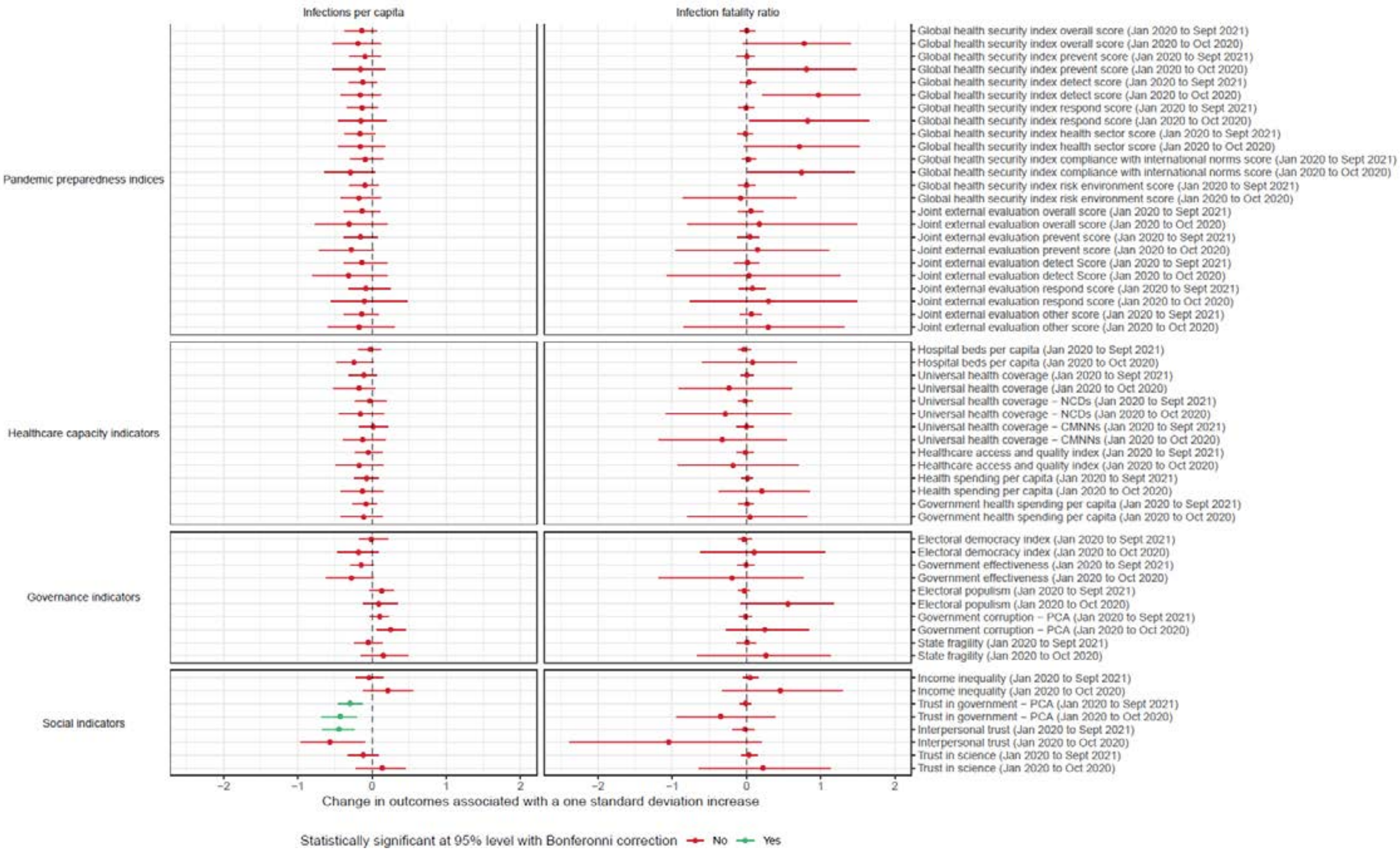


## 5.5 Analyses using only reported COVID deaths

### Figure 5.5.1: Infections vs. IFR

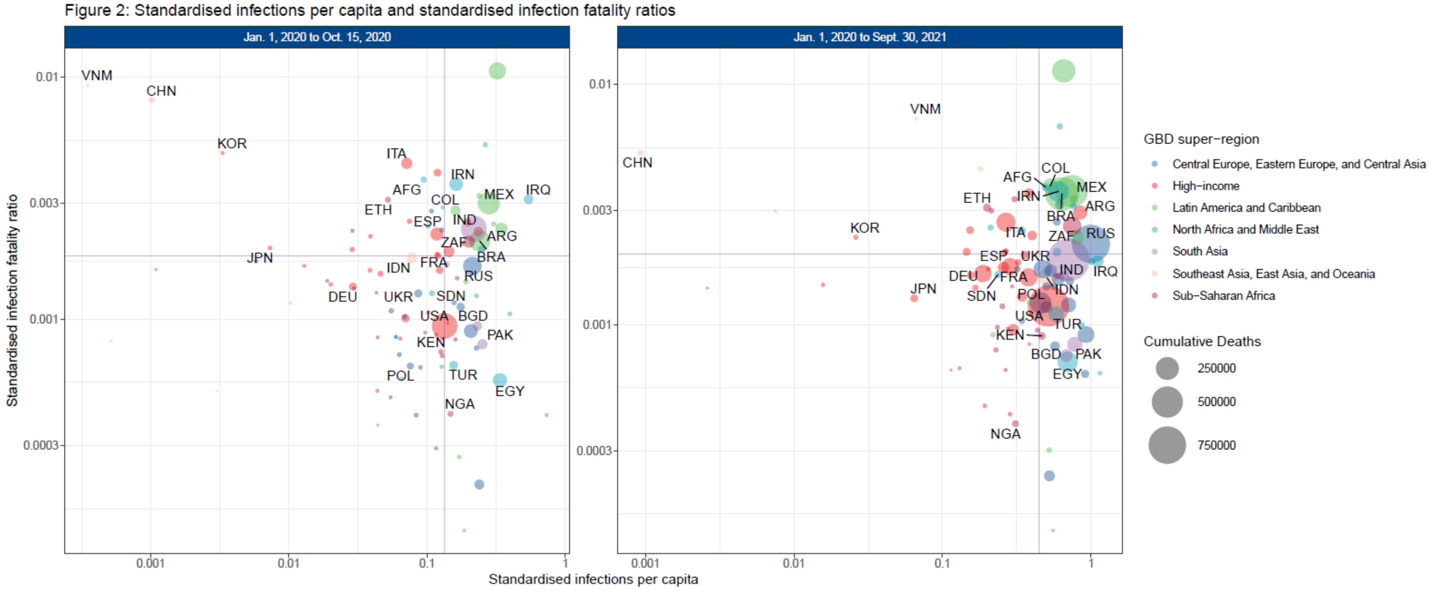

# Figure 5.5.2 Bivariate plots

Figure 3: Associations between key preparedness, capacity, governance, and social indicators and infections and infection fatality ratio

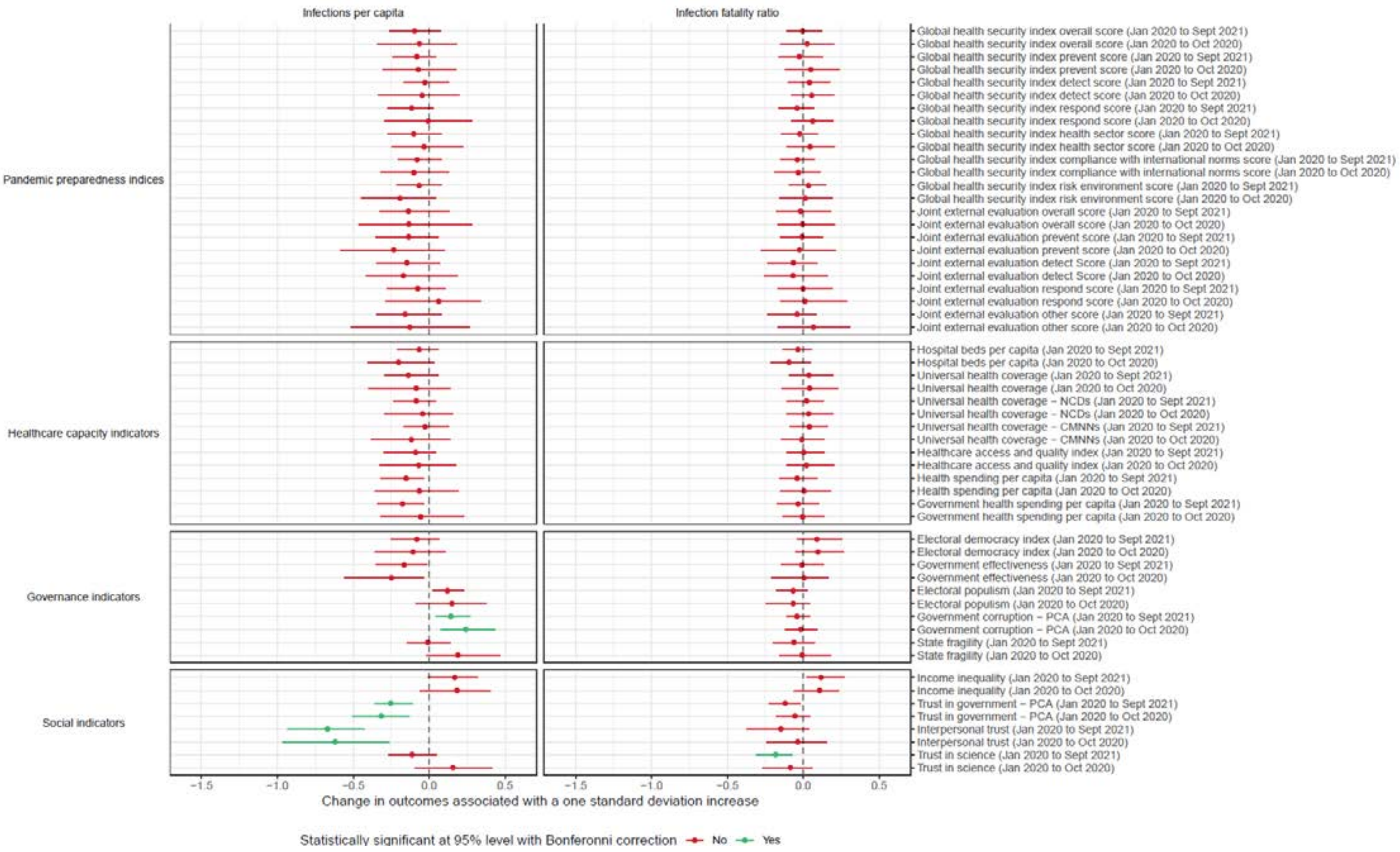


# 5.6 Scatter of raw data versus adjusted for stage 1 variables – IFR and cumulative infections

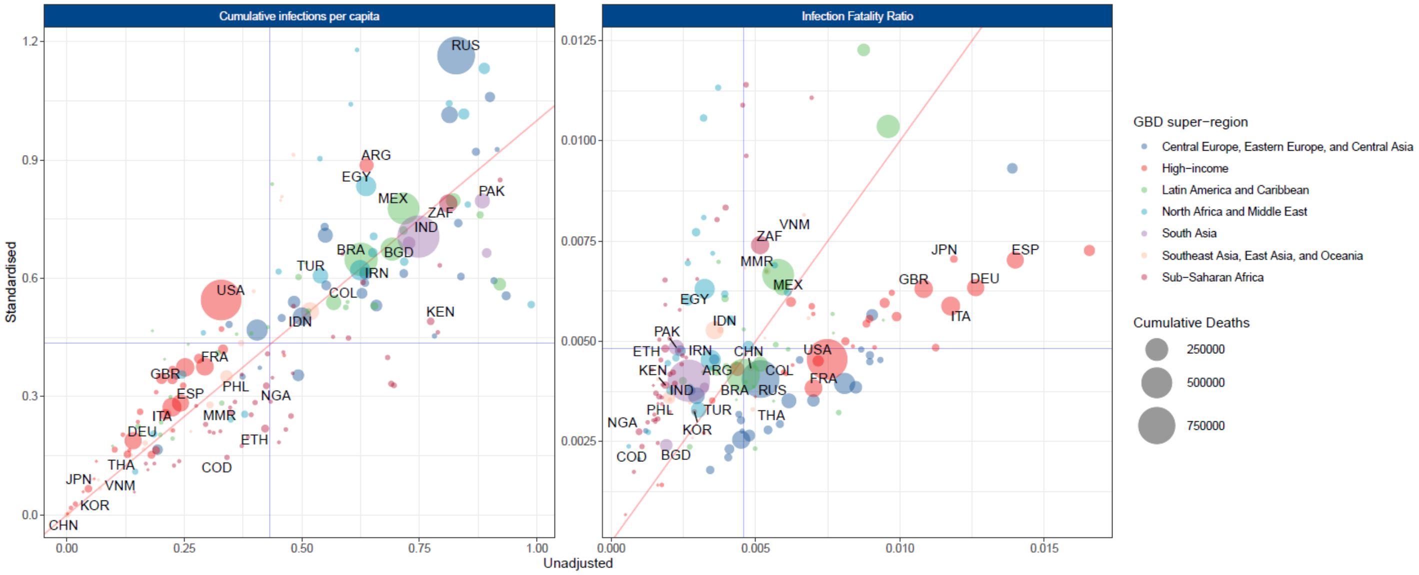

### 5.7 Using 2021 Global Health Security Index metrics in lieu of 2019 metrics, bivariate results

Figure 3: Associations between key preparedness, capacity, governance, and social indicators and infections and infection fatality ratio

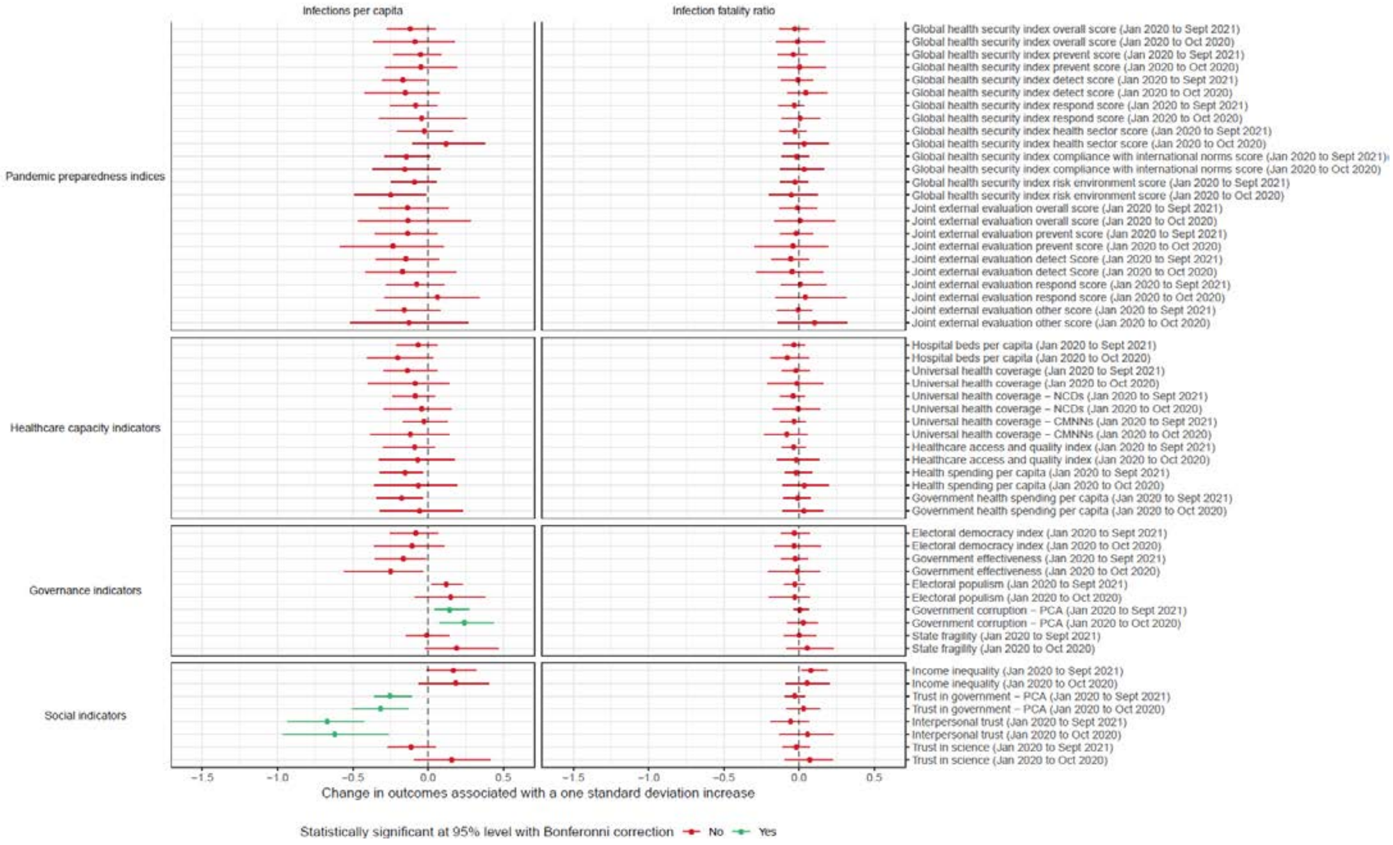


# Section 6: Author Contributions

### Managing the estimation or publications process

Erin N Hulland and Joseph L Dielemen

### Writing the first draft of the manuscript

Thomas J Bollyky and Erin N Hulland

### Primary responsibility for applying analytical methods to produce estimates

Erin N Hulland, Reed J D Sorenson, and Mark Moses

### Primary responsibility for seeking, cataloguing, extracting, or cleaning data; designing or coding figures and tables

Bree Bang-Jensen, Emily Combs, Joseph L Dieleman, Samuel B Ewald, Alize J Ferrari, John Gallagher, Gaorui Guo, Monika Helak, Erin N Hulland, Casey Johanns, Kate E LeGrand, Akiaja Lindstrom, Ana M Mantilla Herrera, Ali H Mokdad, Mark Moses, Christopher J L Murray, Paulami Naik, James Kevin O'Halloran, Maja Pasovic, Louise Penberthy, David M Pigott, Damian Francesco Santomauro, Reed J D Sorensen, Emma Spurlock, and Anh T Vo.

### Providing data or critical feedback on data sources

Cristiana Abbafati, Christopher Adolph, Bree Bang-Jensen, Thomas J Bollyky, Rachel Castellano, Suman Chakrabarti, Xiaochen Dai, William James Dangel, Carolyn Dapper, Joseph L Dieleman, Bruce B Duncan, Lucas Earl, Megan Erickson, Abraham D Flaxman, Nancy Fullman, Emmanuela Gakidou, Bayan Galal, John Gallagher, John R Giles, Gaorui Guo, Monika Helak, Erin N Hulland, Bulat Idrisov, Samantha Kiernan, Kate E LeGrand, Ian D Letourneau, Rafael Lozano, Beatrice Magistro, Deborah Carvalho Malta, Johan Månsson, Fatima Marinho, Alemnesh H Mirkuzie, Ali H Mokdad, Lorenzo Monasta, Christopher J L Murray, Shuhei Nomura, Latera Tesfaye Olana, Maja Pasovic, Valeria Maria de Azeredo Passos, David M Pigott, Grace Reinke, Damian Francesco Santomauro, Maria Inês Schmidt, Reed J D Sorensen, Emma Spurlock, Christopher E Troeger, Anh T Vo, Rebecca Walcott, Simon D Wigley, Charles Shey Wiysonge, Nahom Alemseged Worku, Yifan Wu, and Sarah Wulf Hanson.

### Developing methods or computational machinery

Adrien Allorant, Aleksandr Y Aravkin, Ryan M Barber, Austin Carter, Emma Castro, Suman Chakrabarti, James K Collins, Xiaochen Dai, William James Dangel, Joseph L Dieleman, Tatiana Fedosseeva, Abraham D Flaxman, John R Giles, Gaorui Guo, Jiawei He, Erin N Hulland, Emily Linebarger, Ali H Mokdad, Mark Moses, Christopher J L Murray, Robert C Reiner Jr, Aleksei Sholokhov, Reed J D Sorensen, Christopher E Troeger, Sarah Wulf Hanson, and Peng Zheng.

### Providing critical feedback on methods or results

Cristiana Abbafati, Christopher Adolph, Ryan M Barber, Thomas J Bollyky, James K Collins, Xiaochen Dai, Joseph L Dieleman, Emmanuela Gakidou, Simon I Hay, Erin N Hulland, Paulo A Lotufo, Rafael Lozano, Deborah Carvalho Malta, Alemnesh H Mirkuzie, Ali H Mokdad, Christopher J L Murray, Latera Tesfaye Olana, Samuel M Ostroff, Maja Pasovic, David M Pigott, Robert C Reiner Jr, Reed J D Sorensen, Emma Spurlock, Christopher E Troeger, Theo Vos, Ally Walker, Simon D Wigley, Charles Shey Wiysonge, and Nahom Alemseged Worku.

### Drafting the work or revising is critically for important intellectual content

Cristiana Abbafati, Thomas J Bollyky, James K Collins, Joseph L Dieleman, Simon I Hay, Erin N Hulland, Paulo A Lotufo, Deborah Carvalho Malta, Alemnesh H Mirkuzie, Ali H Mokdad, Lorenzo Monasta, Christopher J L Murray, Reed J D Sorensen, Elena Varavikova, and Charles Shey Wiysonge.

### Managing the overall research enterprise

Joanne O Amlag, William James Dangel, Amanda Deen, Joseph L Dieleman, Simon I Hay, Erin N Hulland, Bethany M Huntley, Ali H Mokdad, Christopher J L Murray, Christopher M Odell, and Emma Spurlock.

## Section 7: References

1 Modeling COVID-19 scenarios for the United States | Nature Medicine. https://www.nature.com/articles/s41591-020-1132-9 (accessed May 5, 2021).

2 GBD 2019 Risk Factors Collaborators. Global burden of 87 risk factors in 204 countries and territories, 1990-2019: a systematic analysis for the Global Burden of Disease Study 2019. *Lancet* 2020; **396**: 1223–49.

3 Lozano R, Fullman N, Mumford JE, *et al.* Measuring universal health coverage based on an index of effective coverage of health services in 204 countries and territories, 1990–2019: a systematic analysis for the Global Burden of Disease Study 2019. *The Lancet* 2020; **396**: 1250–84.

4 Haerpfer C, Inglehart R, Moreno A, *et al.* World Values Survey: Round Seven -Country-Pooled Datafile. *Madrid, Spain & Vienna, Austria: JD Systems Institute & WVSA Secretariat* 2020. DOI:doi.org/10.14281/18241.1.

5 Gallup (2019) Wellcome Global Monitor – First Wave Findings.

6 Gallup Analytics World Poll 2018 - Government and Politics.

7 Transparency International. CORRUPTION PERCEPTIONS INDEX. 2019. https://www.transparency.org/en/cpi/2019/index/nzl.

8 Shahpar C, Lee CT, Wilkason C, Buissonnière M, McClelland A, Frieden TR. Protecting the world from infectious disease threats: now or never. *BMJ Glob Health* 2019; **4**. DOI:10.1136/bmjgh-2019-001885.

9 The Global Health Security Index. GHS Index. https://www.ghsindex.org/ (accessed May 5, 2021).

10 Solt, Frederick. The Standardized World Income Inequality Database, Versions 8. Harvard Dataverse, 2019. https://doi.org/10.7910/DVN/LM4OWF

11 Bosancianu CM, Dionne KY, Hilbig H, *et al.* Political and Social Correlates of Covid-19 Mortality. SocArXiv, 2020 DOI:10.31235/osf.io/ub3zd.

12 Kyle, Jordan, and Brett Meyer. 2020. "High Tide? Populism in Power, 1990-2020." Working Paper. Tony Blair Institute

13 The World Bank. Worldwide Governance Indicators. https://databank.worldbank.org/databases/governance-effectiveness

14 The Fund for Peace. Fragile States Index. https://fragilestatesindex.org/

15 Cruz C, Keefer P, Scartascini C. The Database of Political Institutions 2020 (DPI2020). *Inter-American Development Bank* 2021; published online Jan. DOI:http://dx.doi.org/10.18235/0003049.

16 Coppedge M, Gerring J, Knutsen CH, et al. V-Dem Dataset v10. *Varieties of Democracy (V-Dem) Project* 2020. DOI:https://doi.org/10.23696/vdemds20.

17 Pemstein D, Marquardt K, Tzelgov E, et al. The V-Dem Measurement Model: Latent Variable Analysis for Cross-National and Cross-Temporal Expert-Coded Data.V-Dem Working Paper No. 21. *University of Gothenburg: Varieties of Democracy Institute*; **6th edition**.

18 Pemstein D, Marquardt K, Tzelgov E, et al. The V-Dem Measurement Model: Latent Variable Analysis for Cross-National and Cross-Temporal Expert-Coded Data.V-Dem Working Paper No. 21. *University of Gothenburg: Varieties of Democracy Institute*; **5th edition**.

19 Coppedge M, Gerring J, Knutsen CH, et al. V-Dem Dataset v11. *Varieties of Democracy (V-Dem) Project* 2021. DOI:https://doi.org/10.23696/vdemds21.

20 Global Burden of Disease Health Financing Collaborator Network. Tracking Development Assistance for Health and for COVID-19: a review of development assistance, government, out-of-pocket, and other private spending on health for 204 countries and territories, 1990-2050. *Lancet* Forthcoming.

21 IUCN. The IUCN Red List of Threatened Species. 2021 https://www.iucnredlist.org.

22 Becker DJ, Albery GF, Sjodin AR, *et al.* Predicting wildlife hosts of betacoronaviruses for SARS-CoV-2 sampling prioritization: a modeling study. *bioRxiv* 2020; 2020.05.22.111344.

23 Le Bert N, Tan AT, Kunasegaran K, *et al.* SARS-CoV-2-specific T cell immunity in cases of COVID-19 and SARS, and uninfected controls. *Nature* 2020; **584**: 457–62.

24 Huang J, Teoh JY-C, Wong SH, Wong MCS. The potential impact of previous exposure to SARS or MERS on control of the COVID-19 pandemic. *Eur J Epidemiol* 2020; **35**: 1099–103.

25 Pinotti F, Wikramaratna PS, Obolski U, *et al.* Potential impact of individual exposure histories to endemic human coronaviruses on age-dependent severity of COVID-19. *BMC Medicine* 2021; **19**: 19.

26 Josse J, Husson F. missMDA: A Package for Handling Missing Values in Multivariate Data Analysis. *Journal of Statistical Software* 2016; **70**: 1–31.
